# Towards multivalent CD1d ligands: synthesis and biological activity of homodimeric α-galactosyl ceramide analogues

**DOI:** 10.1016/j.carres.2012.02.034

**Published:** 2012-07-15

**Authors:** Peter J. Jervis, Marie Moulis, John-Paul Jukes, Hemza Ghadbane, Liam R. Cox, Vincenzo Cerundolo, Gurdyal S. Besra

**Affiliations:** a School of Biosciences, University of Birmingham, Edgbaston, Birmingham B15 2TT, UK; b School of Chemistry, University of Birmingham, Edgbaston, Birmingham B15 2TT, UK; c Medical Research Council Human Immunology Unit, Nuffield Department of Medicine, Weatherall Institute of Molecular Medicine, University of Oxford, Oxford OX3 9DS, UK

**Keywords:** α-GalCer, Dimer, Multivalency, CD1d, *i*NKT cells, Click chemistry

## Abstract

A library of dimeric CD1d ligands, containing two α-galactosyl ceramide (α-GalCer) units linked by spacers of varying lengths has been synthesised. The key dimerisation reactions were carried out via copper-catalysed click reactions between a 6″-azido-6″-deoxy-α-galactosyl ceramide derivative and various diynes. Each α-GalCer dimer was tested for its ability to stimulate *i*NKT cells.

## Graphical abstract

**Figure:**
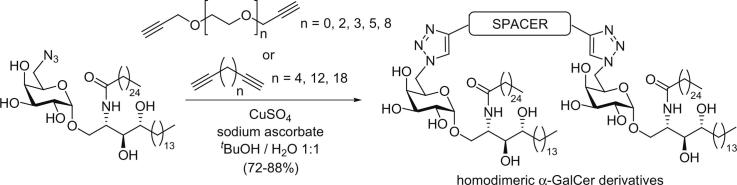

## 1 Introduction

**Figure 1:**
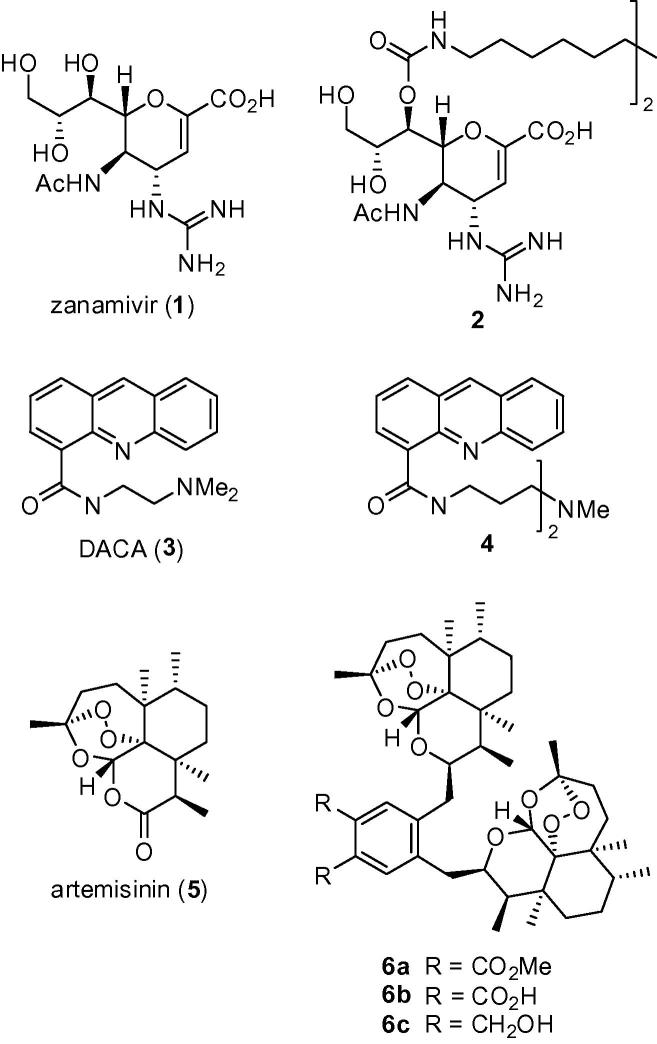
Selected examples of synthetic homodimers which possess increased, or different, biological activity compared with their natural monomeric counterparts.

A multimeric version of a monomeric ligand can achieve higher affinity and specificity for a target receptor.^1^, ^2^, ^3^ As a result, incorporating multiple copies of an active pharmacophore within a single molecule can increase biological activity by several orders of magnitude. These observations have opened up the development of so-called multivalent drug candidates as an active area of research.^4^, ^5^ There are now numerous examples of synthetic homodimers, which represent the simplest form of a multivalent compound, exhibiting significantly improved potency over their monomeric counterparts.^6^, ^7^, ^8^, ^9^, ^10^, ^11^ For example, dimeric derivative **2** of zanamivir (**1**), an influenza virus neuraminidase inhibitor, was found to be 100-fold more potent an inhibitor of influenza virus replication, both in vitro and in vivo, than its monomer analogue.^6^ In a second example, certain dimeric versions (including **4**) of the DNA intercalator *N*-[(2-dimethylamino)ethyl]acridine (DACA, **3**) displayed five times the cytotoxic potency of the parent monomer.^12^, ^13^ In other cases, the dimeric molecule exhibits different properties entirely from the corresponding monomer. For example, artemisinin (**5**) is an antimalarial agent, whilst its homodimeric analogues **6a**–**c** exhibit potent anti-tumour activity (Fig. 1).^14^

**Figure 2:**
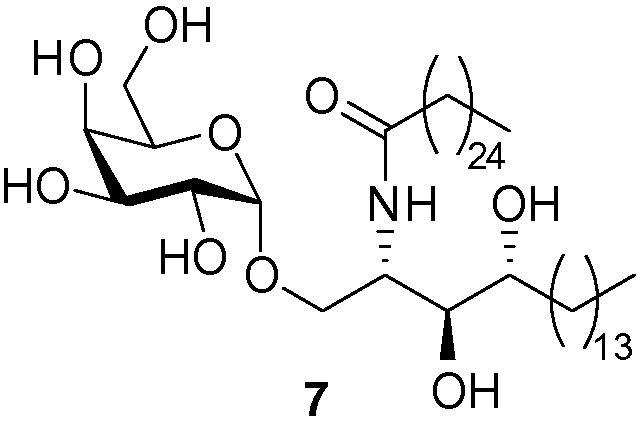
The prototypical *i*NKT cell agonist KRN7000 (α-GalCer) **7**.

As part of a wider research programme into the development of novel CD1d ligands,^15^, ^16^, ^17^, ^18^ we targeted a series of dimeric analogues of the prototypical invariant natural killer T (*i*NKT) cell agonist, α-galactosyl ceramide (α-GalCer, KRN7000 (**7**), Figure 2). α-GalCer^19^ **7** is a synthetic glycolipid, which binds to the non-polymorphic MHC-class-I-like molecule, CD1d. The resulting glycolipid–CD1d complex is recognised by T cell receptors (TCRs) located on the surface of *i*NKT cells. Following recognition of the α-GalCer–CD1d complex, *i*NKT cells rapidly release a diverse array of both pro-inflammatory (Th1) and regulatory (Th2) cytokines and initiate a potent immune response. This method of activating the immune system is currently being explored as a mechanism for ‘boosting’ current vaccination strategies.^20^, ^21^, ^22^, ^23^, ^24^, ^25^, ^26^, ^27^, ^28^, ^29^, ^30^, ^31^, ^32^

Localised clustering of CD1d molecules has been shown to play an important role in antigen presentation. Park et al. have shown that lipid rafts are essential for efficient TCR recognition of CD1d,^33^ whilst Im et al. have reported that α-GalCer **7** requires intracellular loading on to CD1d molecules, which leads to the organised transport of α-GalCer–CD1d complexes into cholesterol-rich lipid rafts.^34^ This localisation of CD1d molecules in membrane rafts serves to concentrate CD1d–lipid complexes in the plasma membrane.^35^ We hypothesised that bivalent ligands of the α-GalCer pharmacophore might serve to stabilise these lipid rafts by forming a cross-linked matrix within the raft structure. This, in turn, might result in more stable CD1d–glycolipid complexes as a result of a chelating effect.^36^, ^37^, ^38^ With an appropriate linker separating the two α-GalCer units, the dimer might be able to bind two CD1d molecules whilst still allowing space for presentation to *i*NKT TCRs.^39^, ^40^ The length of the linking unit in such ligands would be of key importance for optimal divalent binding; too long and it might provide too large a containment volume for the second pharmacophore and would result in the two α-GalCer moieties effectively behaving as unconnected entities; too short,^41^, ^42^ and steric hindrance might block the approach of a second CD1d molecule, although in this case, enhanced binding might still be observed as the result of a statistical re-binding effect.^10^ In the event that a dimer with a shorter linker is able to bind two CD1d molecules, the presence of a second α-GalCer–CD1d complex in close proximity might still serve to block the approach of the TCR.^39^, ^40^ In this way, a dimer could function as an antagonist and yet has signal transduction applications.^11^, ^43^ We now report the synthesis and initial biological results of a range of homodimers of α-GalCer, which vary in both the length and type of linker unit.

## 2 Design and synthesis

Our first consideration in the design of bivalent CD1d ligands was the selection of an appropriate position from which to append a linker to the α-GalCer molecule. The crystal structure of the CD1d–glycolipid–*i*NKT TCR complex^39^, ^40^ reveals that the 2-, 3- and 4-hydroxyl groups of the sugar head group are all involved in hydrogen bonding to either the CD1d molecule or *i*NKT TCR; modifying these positions leads to reduced, or even complete loss, of activity.^44^, ^45^, ^46^ In contrast, it has been shown that the *i*NKT TCR–glycolipid–CD1d interaction can tolerate derivatisation at C6 of the galactose unit and there are a number of biologically active CD1d agonists where the 6-hydroxyl group of the sugar has been replaced with another functional group.^47^, ^48^, ^49^, ^50^ These results are supported by the crystal structure of the CD1d–α-GalCer complex^39^ and a crystal structure of a TCR–α-GalCer–CD1d complex,^40^ which reveal that the 6-OH of the α-GalCer sugar head group is not directly involved in hydrogen bonding to either the CD1d molecule or the *i*NKT TCR. For these reasons, we selected the 6-position of the sugar head group as the most suitable site through which to link together our α-GalCer monomer units.

**Scheme 1:**
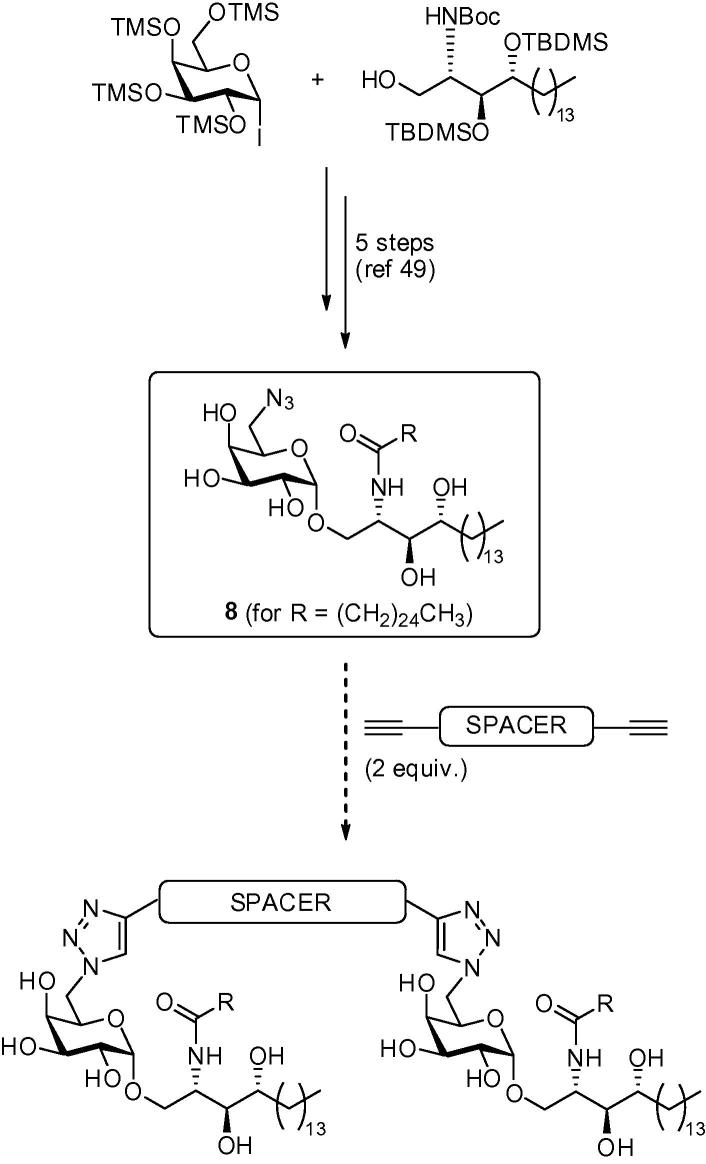
Proposed synthesis of α-GalCer homodimers.

We have recently developed a facile route to 6″-azido-6″-deoxy-α-galactosyl ceramide **8**,^51^ which we considered a potential advanced intermediate for building α-GalCer dimers. We have shown that the azido moiety of **8** can serve as a masked amine, allowing further elaboration to amides, carbamates and ureas; however more directly, the 6″-azido group is also primed for click chemistry. We therefore postulated that a series of 1,2,3-triazole derivatives would be readily available through a simple Huisgen reaction with an appropriate alkyne.^52^, ^53^, ^54^ We envisaged that α-GalCer dimers could be accessed in a single step via a ‘double click’ reaction between 2 equiv of azide **8** and an appropriate diyne (Scheme 1). This approach would also deliver symmetrical homodimers, which would simplify characterisation and analysis.

Poly(ethylene glycol) spacers were chosen to link the α-GalCer units together. These units offer compatibility with biological environments^55^ as well as increased solubility of the amphiphilic glycolipid units in both aqueous and organic solvents.^56^, ^57^ We also chose to investigate alkylene linkers as a hydrophobic comparison. Alkyne-terminated versions of these two types of linkers are readily accessible and would allow the systematic variation of linker lengths.

**Scheme 2:**
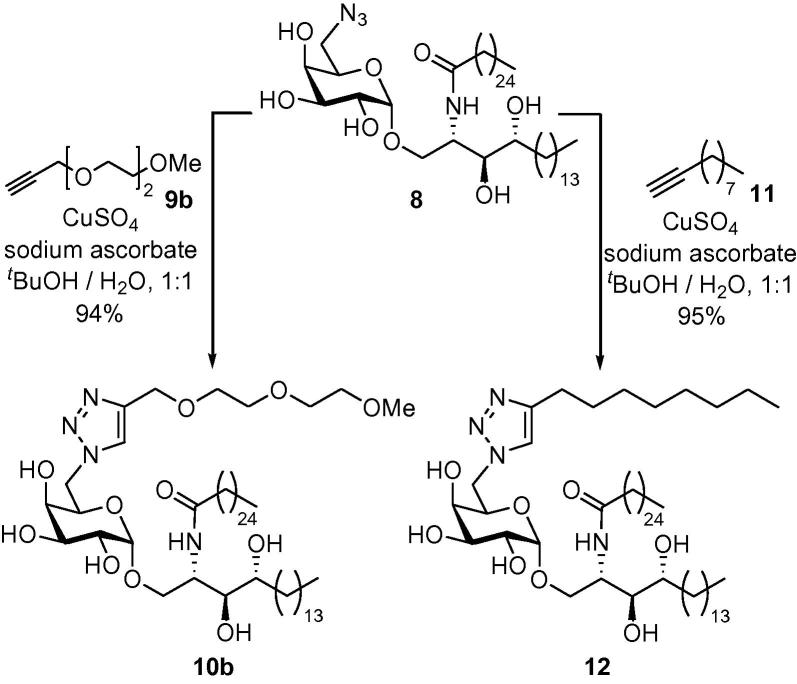
Synthesis of triazole-containing α-GalCer monomers.

In order to test the tolerance of the CD1d–glycolipid–*i*NKT TCR complex to these types of linker units, monomeric α-GalCer analogues **10b** and **12** were first synthesised from azide **8** from alkynes **9b** and **11**, respectively (Scheme 2), and incubated with splenocytes from wildtype C57 BL/6 mice. The presence of IFNγ was then determined by ELISA as previously described by Reddy et al.^58^ Pleasingly, triazole **10b** was found to stimulate *i*NKT cells in vitro at similar levels to α-GalCer **7**, whilst triazole **12** stimulated *i*NKT cells, albeit to a lesser extent (Fig. 5, vide infra).

**Figure 3:**
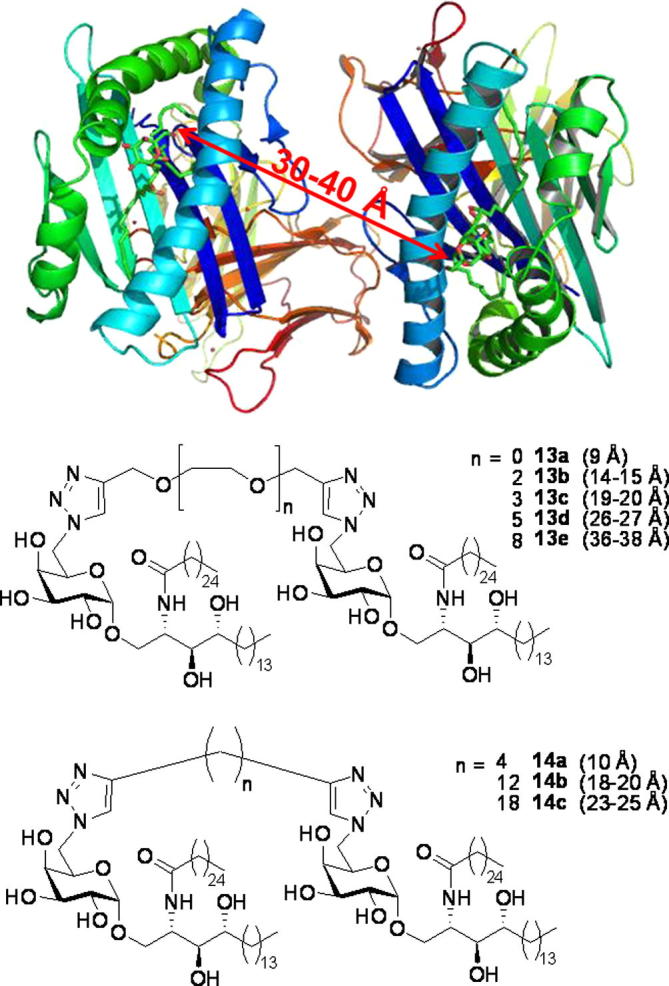
Distances between pharmacophores of target α-GalCer dimers **13a**–**e** and **14a**–**c**.

With the knowledge that the CD1d–glycolipid–*i*NKT TCR interaction tolerated this type of C6 derivatisation, our next consideration was the length of the spacer units to be employed in our dimer syntheses. Major histocompatibility complex (MHC) molecules, which present peptide fragments to T-cells, are structurally similar to CD1d molecules, and in a related study, Cebecauer et al. have studied MHC–peptide dimers.^59^ Dimers containing short linkers, 10–30 Å in length,† efficiently triggered intracellular calcium mobilisation and phosphorylation in cloned cytotoxic T lymphocytes, whereas dimers with longer linkers did not. The fact that MHC–peptide dimers containing linkers as short as 10 Å could still interact with the TCR raised the question of how the two MHC molecules align themselves. The authors describe a dimeric binding model in which two TCRs engage with their α3 domains in an anti-parallel manner, two MHC–peptide complexes facing each other. Models of adjacent MHC molecules revealed distances varying from 12 Å to 46 Å between the α3 domains.^59^ Applying similar orientations to CD1d models (Fig. 3, top), we proposed a range of α-GalCer dimers where the lengths of the spacer units separating the glycolipid pharmacophores could be systematically varied across a similar range. More specifically, we targeted α-GalCer dimers **13a**–**e** and **14a**–**c**, in which the fully extended linker lengths range from 9 Å (for **13a**) to 38 Å (for **13e**), as estimated from models using Pro-DRG server (Fig. 3, bottom).

**Scheme 3:**
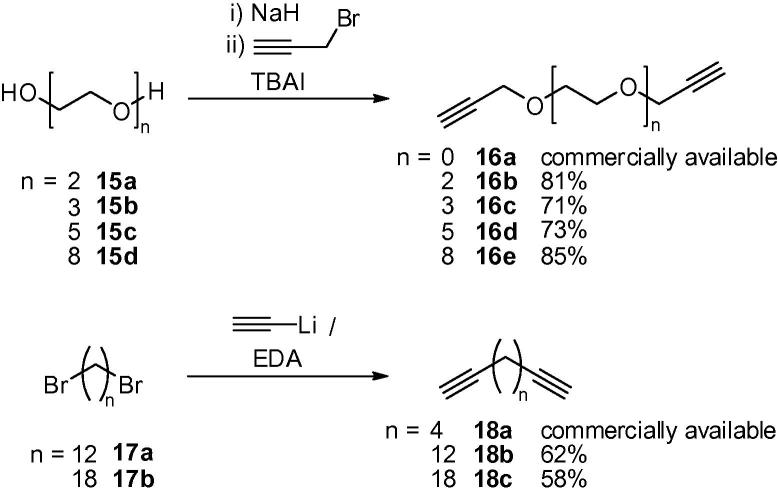
Synthesis of diynes **16b**,**c** and **18b**,**c**.

We first required our series of diynes. Dipropargyl ether **16a** is available commercially, whilst bis-propargylated di-, tri-, penta- and octa(ethylene glycol)s **16b**–**e**, respectively, were prepared in good yield by double deprotonation of the appropriate diol **15a**–**d** with an excess of sodium hydride, followed by addition of an excess of propargyl bromide in the presence of tetrabutylammonium iodide (TBAI). For the alkylene diyne precursors, 1,7-octadiyne (**18a**) is commercially available, whilst 1,15-hexadecadiyne (**18b**) and 1,21-docosadiyne (**18c**) were synthesised in moderate yields by treating 1,12-dibromododecane (**17a**) and 1,18-dibromooctadecane (**17b**), respectively, with 2 equiv of lithium acetylide–ethylenediamine (EDA) complex (Scheme 3).

**Scheme 4:**
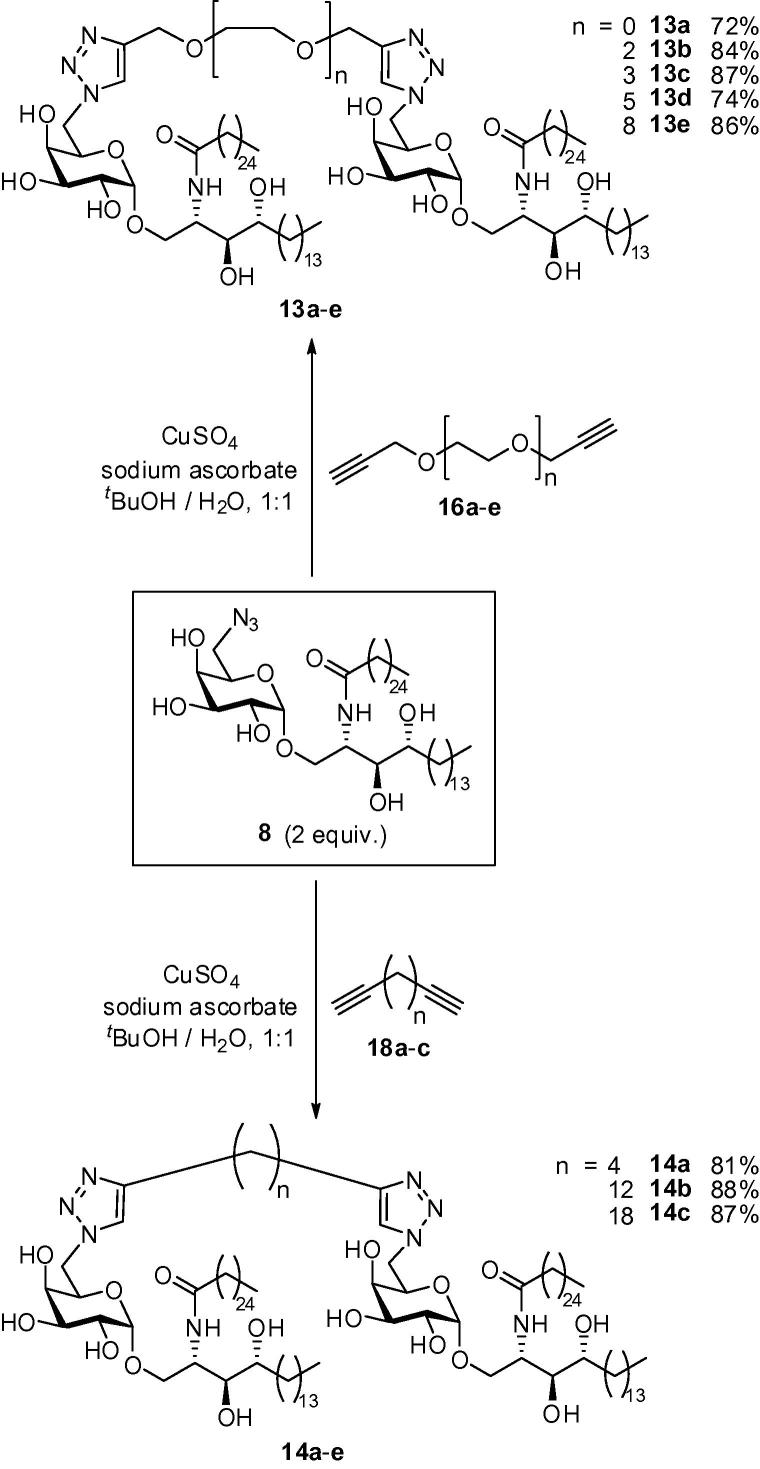
Synthesis of α-GalCer dimers **13a**–**e** and **14a**–**c**.

Diynes **16a**–**e** and **18a**–**c** were next employed in click reactions with azide **8**. Thus, two equivalents of azide **8** and one equivalent of diyne **16a**–**e** or **18a**–**c** were heated in the presence of copper(II) sulfate and sodium ascorbate, to provide bis-triazole dimers **13a**–**e** and **14a**–**c**, respectively, in excellent yields (Scheme 4). These compounds were then tested for their ability to stimulate *i*NKT cells.

## 3 Biological results

**Figure 4:**
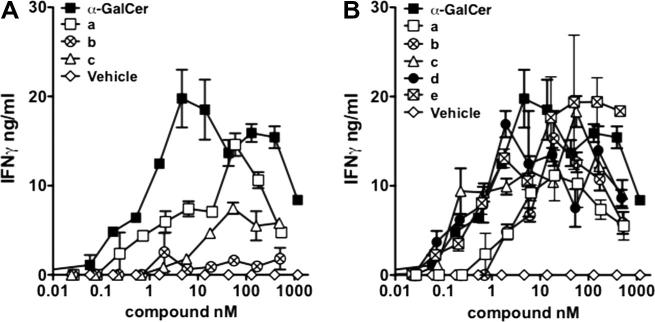
In vitro activation of *i*NKT cells by dimers **14a**–**c** and **13a**–**e**. Splenocytes from C57 BL/6 mice were cultured in the presence of various concentrations of lipids (**14a**–**c**, panel A; **13a**–**e**, panel B). The supernatants were analysed for the presence of IFNγ by ELISA following 48 h of culture.^58^

All of the α-GalCer dimers were found to be active following incubation with splenocytes from wildtype C57 BL/6 mice (Fig. 4);^58^ PEG-linked dimers **13a**–**e** stimulated *i*NKT cells in vitro at similar levels to α-GalCer **7** at high concentrations (>10 nM), although there was no obvious sensitivity to linker length. At lower concentrations (<1 nM), dimers **13a** and **13b**, both of which possess short linkers between the α-GalCer residues, were less active than dimers **13c**–**e**, which contain longer linkers. Alkylene-linked dimers **14a**–**c** also stimulated *i*NKT cells in vitro, but to a lesser extent than α-GalCer **7**. No detectable IFNγ was observed when compounds **13a**–**e** and **14a**–**c** were incubated with splenocytes from CD1d^−/−^ (*i*NKT cell-deficient) mice (data not shown), indicating that the observed *i*NKT cell stimulation by our α-GalCer dimers is CD1d-dependent.

**Figure 5:**
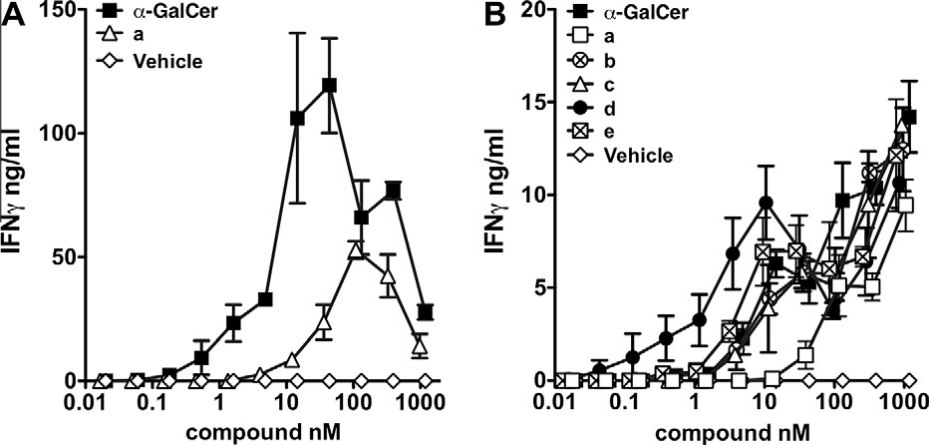
In vitro activation of *i*NKT cells by monomers **12** and **10a**–**e**. Splenocytes from C57 BL/6 mice were cultured in the presence of various concentrations of lipids (**12**, panel A; **10a**–**e**, panel B). The supernatants were analysed for the presence of IFNγ by ELISA following 48 h of culture.^58^

**Figure 6:**
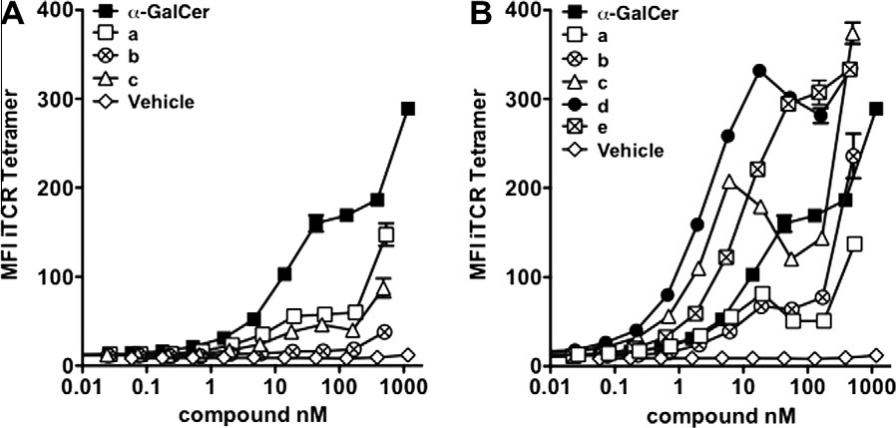
*i*NKT cell recognition of **14a**–**c** and **13a**–**e**. The recognition of **14a**–**c** (panel A) and **13a**–**e** (panel B) by human *i*NKT TCR tetramer was assessed by flow cytometry following co-incubation of fluorescent human *i*NKT cell TCR and hCD1d C1R cells loaded with the indicated lipids.^60^ MFI = Median Fluorescent Intensity.

In order to test the ability of the *i*NKT TCR to recognise the CD1d–glycolipid complex, in vitro binding experiments were performed (Fig. 6).^60^ Dimers **13c**–**e** were comparable to α-GalCer **7** at high concentrations (>10 nM), whilst dimers **13a**,**b** again proved to be considerably weaker than α-GalCer **7**. At lower concentrations (<5 nM), dimers **13c**–**e**, which contain the longer linker units, displayed greater *i*NKT-cell recognition than α-GalCer **7**. Dimer **13d**, which contains a penta(ethylene glycol) linker (∼26–27 Å fully extended length), proved optimal. Alkylene-linked dimers **14a**–**c** showed weaker CD1d binding than α-GalCer **7** (Fig. 6) and we tentatively postulate that the lower activity of this series of dimers might be a result of the hydrophobic alkylene linker coiling to minimise contact with the aqueous medium. This coiling will serve to decrease the effective length of the linker and may account for the observed activity of these dimers more closely resembling that observed for the ethylene glycol dimers containing shorter linkers (i.e., **13a**,**b**).

**Scheme 5:**
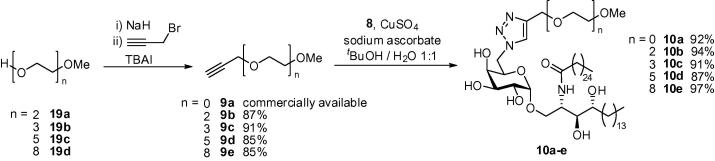
Synthesis of monomers **10a**–**e**.

In order to investigate whether or not the trends observed with dimers **13a**–**e** were a consequence of the presence of the second pharmacophore or due to the presence of the linker unit alone, we targeted a series of monomers **10a**–**e** each containing a PEG tail length which corresponded to the same linker lengths separating the α-GalCer residues in dimers **13a**–**e**. Monomers **10a**–**e** were readily accessed via click reactions between azide **8** and alkynes **9a**–**e**. Alkyne **9a** was available commercially, whilst alkynes **9b**–**e** were readily synthesised from alcohols **19a**–**d** (Scheme 5).

Each of the monomers **10a**–**e** was tested for its ability to stimulate *i*NKT cells (Fig. 5). α-GalCer **7** served as a reference compound, enabling us to compare the data for monomers **10a**–**e** with the data for the corresponding dimers **13a**–**e**. At high concentrations (>10 nM), the level of *i*NKT-cell stimulation for all of the monomers **10a**–**e** was not significantly different to that observed for the corresponding dimers (relative to α-GalCer **7** reference). Surprisingly, at lower concentrations (<1 nM), monomer **10d** proved to be a more potent activator of *i*NKT cells than its dimeric equivalent (**13d**), relative to α-GalCer **7**, whilst **10a**, the control for **13a**, was again inactive at low concentrations in vitro. The fact that dimers **13a**–**e** do not show any enhanced biological activity over their corresponding monomers **10a**–**e** suggests that a bivalent effect is not taking place in vitro. A comparison of the data for monomer **12**, containing an octyl chain, and the alkylene-linked dimers **14a**–**c** also provides little evidence for a bivalent effect.

**Figure 7:**
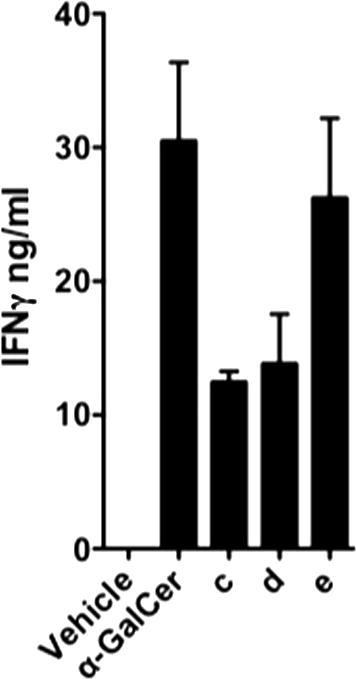
Wildtype C57 BL/6 (n = 3/group) mice were injected intravenously with 1 μg of analogue or vehicle. IFNγ levels in blood serum were determined by ELISA at 18 h *post* injection.^62^

The effect of lipid rafts and clustering effects on *i*NKT-cell activation might be expected to be more apparent in vivo,^61^ and the in vivo results were indeed more interesting. PEG-linked dimers **13c**–**e**, which had proven to be the most active from the in vitro studies, were injected intravenously into C57 BL/6 WT mice. Blood serum was taken at 18 h and the presence of IFNγ determined by ELISA (Fig. 7).^62^ *i*NKT-cell stimulation was found to increase with linker length, up to the point where the dimer with the longest octa(ethylene glycol) linker, **13e**, had almost the same activity as α-GalCer **7**. As before, CD1d^−/−^ (*i*NKT cell-deficient) mice did not have any detectable IFNγ (data not shown), indicating that these results are again CD1d-dependent. These in vivo results raise the question of how this trend of *i*NKT-cell stimulation versus linker length would extrapolate with even longer linkers.

In summary, we have synthesised two series of biologically active dimeric α-GalCer analogues (**13a**–**e** and **14a**–**c**). Initial in vitro experiments showed that dimers based on poly(ethylene glycol) linkers (**13a**–**e**) stimulated *i*NKT cells to a similar extent as α-GalCer **7** for longer linker lengths (**13c**–**e**), whilst those with shorter lengths (**13a**,**b**) were less-effective CD1d agonists. We considered the possibility that this trend might be due to the linking poly(ethylene glycol) unit itself rather than as a result of a bivalent effect and therefore synthesised and tested a series of monomers (**10a**–**e**) containing a poly(ethylene glycol) tail length corresponding to each dimer linker length. These monomers showed a similar trend to the corresponding dimers, with **10a**,**b** being the least active. Surprisingly, monomer **10d** was more active than its corresponding dimer, **13d**. Dimers based on alkylene linkers proved to be less active than α-GalCer **7** in vitro, and showed no obvious sensitivity to linker length. Overall, the in vitro results show little evidence for a bivalent effect. Preliminary in vivo experiments show an increase in activity with increasing linker length, with the octa(ethylene glycol)-linked dimer **13e** being the most potent activator of *i*NKT cells, having a similar activity to α-GalCer **7**. Future work will involve producing dimers and multimers with longer linker lengths and further probing the multivalent effects using different techniques.

## 4 Experimental

### 4.1 General methods

Infra-red spectra were recorded neat as thin films between sodium chloride discs, or as potassium bromide discs. The intensity of each band is described as s (strong), m (medium) or w (weak) and with the prefix v (very) and suffix br (broad) where appropriate. The poor solubility of all α-GalCer dimers at rt prevented us from obtaining reliable optical rotation data. ^1^H NMR and ^13^C NMR chemical shifts are reported as *δ* values (ppm) referenced to the following solvent signals: CHCl_3_, *δ*_H_ 7.26; CDCl_3_, *δ*_C_ 77.0; MeOH, *δ*_H_ 3.31; CD_3_OD, *δ*_C_ 49.0. The term ‘stack’ is used to describe a region where resonances arising from non-equivalent nuclei are coincident, and multiplet, m, to describe a resonance arising from a single nucleus (or equivalent nuclei) but where coupling constants cannot be readily assigned. Mass spectra were recorded on a LCT spectrometer utilising electrospray ionisation with a methanol mobile phase, or electron impact ionisation, and are reported as (*m*/*z* (%)). HRMS were recorded on a LCT spectrometer using a lock mass incorporated into the mobile phase. Melting points were determined using open capillaries and are uncorrected.

### 4.2 Chemicals

All reagents were obtained from commercial sources and used without further purification unless specified otherwise. Tetrahydrofuran was freshly distilled under nitrogen from sodium benzophenone ketyl. All solutions are aqueous and saturated unless specified otherwise.

### 4.3 General procedures

#### 4.3.1 General procedure for bis-propargylation of diols **15a**–**d** and mono-propargylation of alcohols **19a**–**d** (synthesis of diynes **16b**–**e** and alkynes **9b**–**e**)

NaH (60% w/w in mineral oil, 3 equiv (for bis-propargylation) or 1.5 equiv (for mono-propargylation)) was added to a solution of appropriate diol **15ba**–**d** or alcohol **19a**–**d** (1 equiv) in anhydrous THF (0.2 M) at 0 °C under an N_2_ atmosphere. A catalytic amount (spatula tip) of Bu_4_NI (0.05 equiv) and propargyl bromide (3 equiv (for bis-propargylation) or 1.5 equiv (for mono-propargylation)) was added sequentially. The mixture was stirred at rt overnight, before being concentrated under reduced pressure. Purification of the residue by flash column chromatography afforded diynes **16b**–**e** and alkynes **9b**–**e** as pale yellow oils.

#### 4.3.2 General procedure for lithium acetylide addition to dibromoalkanes **17a** and **17b** (synthesis of diynes **18b** and **18c**)

Lithium acetylide–ethylenediamine complex (2 equiv) was added to a solution of dibromoalkane **17a** or **17b** (1 equiv) in DMSO (0.2 M) at 0 °C. The mixture was allowed to warm to rt and stirred overnight. H_2_O (5 × volume) and hexane (5 × volume) were added and the phases were separated. The aqueous phase was extracted with hexane (5 × volume). The combined organic extracts were washed with brine (5 × volume), dried (MgSO_4_), filtered and concentrated under reduced pressure. Purification of the residue by flash column chromatography (hexane) afforded diyne **18b** or **18c** both as white solids.

#### 4.3.3 General procedure for click chemistry (synthesis of dimers **13a**–**e** and **14a**–**c** and monomers **10a**–**e** and **12**)

CuSO_4_ solution (5 μL of 0.5 M solution, 2.5 μmol) and sodium ascorbate solution (18 μL of a 1.0 M solution, 18 μmol) were added to a solution of azide **8** (20 mg, 0.023 mmol) and diyne **14a**–**e** or **16a**–**c** (0.011 mmol) or alkyne **9a**–**e** or **11** (0.023 mmol) in ^*t*^BuOH/H_2_O (1 mL, 1:1) at rt. The reaction mixture was heated for 10 h at 50 °C and then diluted with CHCl_3_ (10 mL), and washed with brine (3 mL). The phases were separated and the aqueous layer was extracted with CHCl_3_ (2 × 5 mL). The combined organic layers were dried over MgSO_4_, filtered and concentrated under reduced pressure. Purification of the residue by flash column chromatography afforded dimers **13a**–**e** and **14a**–**c** and monomers **10a**–**e** and **12**.

### 4.4 Characterisation

#### 4.4.1 Bis-*O*-propargyl-di(ethylene glycol) (**16b**)

Diyne **16b** was prepared from di(ethylene glycol) **15a** (1.00 g, 9.43 mmol) and propargyl bromide (2.54 mL of a 80% w/w solution in toluene, 29.5 mmol) according to Section 4.3.1. After stirring overnight, removal of the solvent and purification of the residue by flash column chromatography (25% EtOAc in hexane) afforded diyne **16b** as a yellow oil (1.38 g, 81%): *R*_f_ = 0.28 (25% EtOAc in hexane); IR (film) *ν* 3252m (≡C–H), 2866m, 2114w (C≡C), 1443m, 1349m, 1288w, 1136s, 1094s, 1033m, 918m, 877w, 843m; ^1^H NMR (300 MHz, CDCl_3_) *δ* 2.42 (t, 2H, *J* 2.4 Hz), 3.65–3.73 (stack, 8H, OC*H*_2_C*H*_2_O), 4.19 (d, 4H, *J* 2.4 Hz); ^13^C NMR: (75 MHz, CDCl_3_) *δ* 58.1 (CH_2_), 68.9 (CH_2_), 70.2 (CH_2_), 74.4 (CH), 79.4 (quat. C); *m*/*z* (TOF ES^+^) 205.1 ([M+Na]^+^, 100%); HRMS *m*/*z* (TOF ES^+^) 205.0847. C_10_H_14_NaO_3_ requires 205.0841.

#### 4.4.2 Bis-*O*-propargyl-tri(ethylene glycol) (**16c**)

Diyne **16c** was prepared from tri(ethylene glycol) **15b** (1.42 g, 9.47 mmol) and propargyl bromide (2.54 mL of a 80% w/w solution in toluene, 29.5 mmol) according to Section 4.3.1. After stirring overnight, removal of the solvent and purification of the residue by flash column chromatography (40% EtOAc in hexane) afforded diyne **16c** as a yellow oil (1.52 g, 71%): *R*_f_ = 0.31 (40% EtOAc in hexane); IR (film) *ν* 3251m (≡C–H), 2868m, 2114w (C≡C), 1443m, 1349m, 1248m, 1093s, 1031s, 917s, 877s, 842s, 731s; ^1^H NMR (300 MHz, CDCl_3_) *δ* 2.43 (t, 2H, *J* 2.4 Hz), 3.65–3.78 (12H, stack), 4.21 (d, 4H, *J* 2.4 Hz); ^13^C NMR (75 MHz, CDCl_3_) *δ* 58.2 (CH_2_,), 68.9 (CH_2_), 70.2 (CH_2_), 70.4 (CH_2_), 74.4 (CH), 79.5 (quat. C); *m*/*z* (TOF ES^+^) 249.1 ([M+Na]^+^, 100%); HRMS *m*/*z* (TOF ES^+^) 249.1094. C_12_H_18_NaO_4_ requires 249.1103.

#### 4.4.3 Bis-*O*-propargyl-penta(ethylene glycol) (**16d**)

Diyne **16d** was prepared from penta(ethylene glycol) **15c** (2.25 g, 9.45 mmol) and propargyl bromide (2.54 mL of 80% w/w solution in toluene, 29.5 mmol) according to Section 4.3.1. After stirring overnight, removal of the solvent and purification of the residue by flash column chromatography (60% EtOAc in hexane) afforded diyne **16d** as a yellow oil (2.17 g, 73%): *R*_f_ = 0.26 (60% EtOAc in hexane); IR (film) *ν* 3247m (≡C–H), 2867s, 2113w (C≡C), 1458m, 1349m, 1289m, 1248m, 1093s, 1032s, 948m, 919m, 842m, 669s; ^1^H NMR (300 MHz, CDCl_3_) *δ* 2.43 (t, 2H, *J* 2.4 Hz), 3.64–3.73 (stack, 20H), 4.20 (d, 4H, *J* 2.4 Hz); ^13^C NMR (75 MHz, CDCl_3_) *δ* 58.3 (CH_2_), 69.1 (CH_2_), 70.3 (CH_2_), 70.5 (CH_2_), 74.5 (CH), 79.6 (quat. C), some overlapping ethylene glycol resonances; *m*/*z* (TOF ES^+^) 337.2 ([M+Na]^+^, 100%); HRMS *m*/*z* (TOF ES^+^) 337.1615. C_16_H_26_NaO_6_ requires 337.1627.

#### 4.4.4 Bis-*O*-propargyl-octa(ethylene glycol) (**16e**)

Diyne **16e** was prepared from octa(ethylene glycol) **15d** (1.00 g, 2.70 mmol) and propargyl bromide (0.73 mL of a 80% w/w solution in toluene, 8.20 mmol) according to Section 4.3.1. After stirring overnight, removal of the solvent and purification of the residue by flash column chromatography (6% MeOH in CHCl_3_) afforded diyne **16e** as a yellow oil (1.03 g, 85%): *R*_f_ = 0.29 (6% MeOH in CHCl_3_); IR (film) *ν* 3247m (≡C–H), 2919m, 2864m, 2112w (C≡C), 1956w, 1458m, 1349m, 1291m, 1248m, 1093s, 1032m, 946m, 840m, 703m, 688m, 675m, 660m; ^1^H NMR (300 MHz, CDCl_3_) *δ* 2.43 (t, 2H, *J* 2.4 Hz), 3.64–3.73 (stack, 32H), 4.20 (d, 4H, *J* 2.4 Hz); ^13^C NMR (75 MHz, CDCl_3_) *δ* 58.2 (CH_2_), 68.9 (CH_2_), 70.1 (CH_2_), 70.3 (CH_2_), 74.4 (CH), 79.5 (quat. C), some overlapping ethylene glycol resonances; *m*/*z* (TOF ES^+^) 469.2 ([M+Na]^+^, 100%); HRMS *m*/*z* (TOF ES^+^) 469.2406. C_22_H_38_NaO_9_ requires 469.2414.

#### 4.4.5 Hexadeca-1,15-diyne (**18b**)

Lithium acetylide–ethylenediamine complex (693 mg, 7.50 mmol) and dibromide **17a** (1.15 g, 3.51 mmol) were reacted according to Section 4.3.2. After stirring overnight, work-up and purification of the residue by flash column chromatography (hexane) afforded diyne **18b** as a white solid (62%): mp 43–45 °C, lit.^63^ 43–44 °C; *R*_f_ = 0.65 (hexane); IR (KBr) *ν* 3286m (≡C–H), 2917m, 2849m, 2115w (C≡C), 1472m, 1462m, 1419w, 1339w, 1280w, 732m, 720m, 666s; ^1^H NMR (300 MHz, CDCl_3_) *δ* 1.13–1.44 (stack, 16H), 1.46–1.57 (m, 4H), 1.93 (t, 2H, *J* 2.6 Hz), 2.17 (td, 4H, *J* 6.9, 2.6 Hz); ^13^C NMR (75 MHz, CDCl_3_) *δ* [18.4, 28.5, 28.8, 29.1, 29.5, 29.6 (CH_2_, overlapping alkyl chain resonances)], 68.0 (CH, C≡*C*H), 84.8 (quat. C, *C*≡CH); *m*/*z* (TOF ES^+^) 241.2 ([M+Na]^+^, 100%); HRMS *m*/*z* (TOF ES^+^) 241.1941. C_16_H_26_Na requires 241.1932.

#### 4.4.6 Docosa-1,21-diyne (**18c**)

Lithium acetylide–ethylenediamine complex (36 mg, 0.39 mmol) and dibromide **17b** (75 mg, 0.18 mmol) were reacted according to Section 4.3.2. After stirring overnight, work-up and purification of the residue by flash column chromatography (hexane) afforded diyne **18c** as a white solid (58%): mp 64–65 °C; *R*_f_ = 0.73 (hexane); IR (KBr) *ν* 3906m (≡C–H), 2917m, 2849m, 2115w (C≡C), 1472m, 1462m, 1419w, 1340w, 1280w, 733m, 720m, 665s; ^1^H NMR (300 MHz, CDCl_3_) *δ* 1.17–1.45 (stack, 28H), 1.46–1.57 (m, 4H), 1.93 (t, 2H, *J* 2.6 Hz), 2.18 (td, 4H, *J* 7.1, 2.6 Hz); ^13^C NMR (75 MHz, CDCl_3_) *δ* [18.4, 28.5, 28.8, 29.1, 29.5, 29.7 (CH_2_, overlapping alkyl chain resonances)], 68.0 (CH), 84.8 (quat. C); *m*/*z* (TOF ES^+^) 325.3 ([M+Na]^+^, 100%); HRMS *m*/*z* (TOF ES^+^) 325.2867. C_22_H_38_Na requires 325.2871.

#### 4.4.7 Bis-1,2,3-triazole **13a** (PEG-0 link)

Azide **8** (20 mg, 0.023 mmol) and diyne **16a** (1.0 mg, 0.011 mmol) were reacted according to Section 4.3.3. After 10 h, work-up and purification of the residue by flash column chromatography (15% MeOH in CHCl_3_) afforded bis-1,2,3-triazole **13a** as a colourless paste (15 mg, 72%): *R*_f_ = 0.22 (15% MeOH in CHCl_3_); IR (KBr) *ν* 3353br s (O–H), 2917s, 2850s, 1638m (C=O), 1544w, 1468m, 1343w, 1230w, 1148m, 1066s, 1035s, 784w, 720s; ^1^H NMR (300 MHz, CDCl_3_/CD_3_OD, 2:1) *δ* 0.85 (app. t, 12H, *J* 6.6 Hz), 1.14–1.36 (stack, 136H), 1.42–1.63 (stack, 8H), 2.14 (app. t, 4H, *J* 6.7 Hz), 3.36–3.40 (m, 2H), 3.45–3.52 (stack, 6H), 3.71 (dd, 2H, *J* 9.9, 3.0 Hz), 3.75–3.83 (stack, 4H), 4.03–4.12 (m, 2H), 4.16 (app. t, 2H, *J* 5.7 Hz), 4.53–4.62 (stack, 8H), 4.86 (d, 2H, *J* 3.6 Hz), 7.89 (br s, 2H), exchangeable protons not observed; ^13^C NMR (75 MHz, CDCl_3_/CD_3_OD, 2:1) *δ* 13.5 (CH_3_), [20.6, 22.2, 25.4, 28.79. 28.85, 28.92, 29.1, 29.2, 31.4, 31.9, 35.9 (CH_2_, overlapping alkyl chain resonances)], 49.6 (CH), 50.5 (CH_2_), 62.6 (CH_2_), 66.6 (CH), 68.1 (CH), 68.9 (2 × CH, resonance overlap), 69.4 (CH_2_), 71.4 (CH), 74.0 (CH), 99.1 (CH), 124.5 (CH), 144.9 (quat. C), 174.0 (quat. C); *m*/*z* (TOF ES^−^) 1859.3 ([M−H]^−^, 100%), 1691.0 (43), 1521.7 (63).

#### 4.4.8 Bis-1,2,3-triazole **13b** (PEG-2 link)

Azide **8** (20 mg, 0.023 mmol) and diyne **16b** (2.0 mg, 0.011 mmol) were reacted according to Section 4.3.3. After 10 h, work-up and purification of the residue by flash column chromatography (15% MeOH in CHCl_3_) afforded bis-1,2,3-triazole **13b** as a colourless paste (18 mg, 84%): *R*_f_ = 0.23 (15% MeOH in CHCl_3_); IR (KBr) *ν* 3351m br (O–H), 2917s, 2850s, 1631m (C=O), 1547w, 1467m, 1349w, 1230w, 1141m, 1064s, 1034s, 785w, 720s; ^1^H NMR (300 MHz, CDCl_3_/CD_3_OD, 2:1) *δ* 0.84 (app. t, 12H, *J* 6.6), 1.14–1.32 (stack, 136H), 1.46–1.61 (stack, 8H), 2.14 (app. t, 4H, *J* 6.7 Hz), 3.32–3.40 (m, 2H), 3.41–3.50 (stack, 6H), 3.61–3.73 (stack, 10H), 3.75–3.83 (stack, 4H), 4.04–4.12 (m, 2H), 4.18 (dt, 2H, *J* 3.3, 3.0 Hz), 4.52–4.59 (stack, 4H), 4.60–4.66 (stack, 4H), 4.85 (d, 2H, *J* 3.6 Hz), 7.84 (br d, 2H, *J* 3.0 Hz), exchangeable protons not observed; ^13^C NMR (75 MHz, CDCl_3_/CD_3_OD, 2:1) *δ* 13.5 (CH_3_), [20.6, 22.2, 25.5, 28.9, 29.0, 29.1, 29.2, 29.3, 29.3, 29.4, 29.8, 31.5, 32.2, 33.8, 36.1 (CH_2_, overlapping alkyl chain resonances)], 49.7 (CH), 50.4 (CH_2_), 63.8 (CH_2_), 66.9 (CH_2_), 68.3 (CH), 68.9 (CH), 69.0 (CH), 69.2 (CH_2_), 69.5 (CH), 70.0 (CH_2_), 71.6 (CH), 74.2 (CH), 99.2 (CH), 124.4 (CH), 144.8 (quat. C), 174.0 (quat. C); *m*/*z* (TOF ES^+^) 1947.9 ([M+H]^+^, 80%), 1107.9 (42), 1025.9 (100).

#### 4.4.9 Bis-1,2,3-triazole **13c** (PEG-3 link)

Azide **8** (20 mg, 0.023 mmol) and diyne **16c** (2.5 mg, 0.011 mmol) were reacted according to Section 4.3.3. After 10 h, work-up and purification of the residue by flash column chromatography (15% MeOH in CHCl_3_) afforded bis-1,2,3-triazole **13c** as a colourless paste (19 mg, 87%): *R*_f_ = 0.23 (15% MeOH in CHCl_3_); IR (KBr) *ν* 3368m br (O–H), 2918s, 2850s, 1634m (C=O), 1552w, 1467s, 1349w, 1231w, 1147m, 1071s, 1037s, 785w, 719m; ^1^H NMR (400 MHz, CDCl_3_/CD_3_OD, 2:1) *δ* 0.85 (app. t, 12H, *J* 6.6 Hz), 1.15–1.33 (stack, 136H), 1.45–1.62 (stack, 8H), 2.14 (app. t, 4H, *J* 7.2 Hz), 3.36–3.43 (m, 2H), 3.43–4.52 (stack, 6H), 4.53–3.60 (m, 2H), 3.60–3.73 (stack, 14H), 3.76–3.81 (m, 2H), 4.06–4.11 (m, 2H), 4.18 (app. t, 2H, *J* 6.0 Hz), 4.55–4.59 (stack, 4H), 4.60–4.64 (stack, 4H), 4.85 (d, 2H, *J* 3.6 Hz), 7.84 (br s, 2H), exchangeable protons not observed; ^13^C NMR (100 MHz, CDCl_3_/CD_3_OD, 2:1) *δ* 13.3 (CH_3_), [22.1, 25.4, 28.7, 28.82, 28.88, 28.93, 29.1, 29.3, 31.4, 31.9, 35.9 (CH_2_, overlapping alkyl chain resonances)], 49.6 (CH), 50.4 (CH_2_), 63.6 (CH_2_), 66.6 (CH_2_), 68.2 (CH), 68.9 (CH), 69.0 (CH), 69.1 (CH_2_), 69.4 (CH), 69.8 (CH_2_), 69.9 (CH_2_), 71.4 (CH), 74.0 (CH), 99.1 (CH), 124.3 (CH), 144.3 (quat. C), 173.9 (quat. C); *m*/*z* (TOF ES^+^) 1992.0 ([M+H]^+^, 30%), 1070.0 (100).

#### 4.4.10 Bis-1,2,3-triazole **13d** (PEG-5 link)

Azide **8** (20 mg, 0.023 mmol) and diyne **16d** (3.5 mg, 0.011 mmol) were reacted according to Section 4.3.3. After 10 h, work-up and purification of the residue by flash column chromatography (20% MeOH in CHCl_3_) afforded bis-1,2,3-triazole **13d** as a colourless paste (18 mg, 79%): *R*_f_ = 0.23 (20% MeOH in CHCl_3_); IR (KBr) *ν* 3369m br (O–H), 2918s, 2850s, 1634m (C=O), 1552w, 1467s, 1349w, 1231w, 1147m, 1070s, 1035s, 785w, 719m; ^1^H NMR (300 MHz, CDCl_3_/CD_3_OD, 2:1) *δ* 0.85 (app. t, 12H, *J* 6.8 Hz), 1.14–1.39 (stack, 136H), 1.47–1.65 (stack, 8H), 2.14 (app. t, 4H, *J* 7.5 Hz), 3.36–3.43 (m, 2H), 3.44–3.53 (stack, 6H), 3.60–3.67 (stack, 20H), 3.71 (dd, 2H, *J* 9.9, 3.3 Hz), 3.76–3.83 (stack, 4H,), 4.03–4.13 (m, 2H), 4.17 (app. t, 2H, *J* 6.5 Hz), 4.53–4.63 (stack, 8H), 4.86 (d, 2H, *J* 3.6 Hz), 7.42 (d, 2H, *J* 8.7 Hz), 7.84 (s, 2H), alcoholic protons not observed; ^13^C NMR (100 MHz, CDCl_3_/CD_3_OD, 2:1) *δ* 13.2 (CH_3_), [22.1, 25.3, 28.78, 28.83, 28.9, 29.1, 29.09, 29.13, 31.4, 31.8, 35.8 (CH_2_, overlapping alkyl chain resonances)], 49.6 (CH), 50.4 (CH_2_), 63.5 (CH_2_), 66.6 (CH_2_), 68.1 (CH), 68.9 (CH), 69.0 (CH), 69.4 (CH), 69.8 (CH_2_, overlapping ethylene glycol resonances), 71.4 (CH), 73.9 (CH), 99.1 (CH), 124.3 (CH), 144.1 (quat. C), 173.9 (quat. C); *m*/*z* (TOF ES^+^) 2079.2 ([M+Na]^+^, 100%), 694.5 (70, [ceramide]^+^).

#### 4.4.11 Bis-1,2,3-triazole **13e** (PEG-8 link)

Azide **8** (20 mg, 0.023 mmol) and diyne **16e** (4.9 mg, 0.011 mmol) were reacted according to Section 4.3.3. After 10 h, work-up and purification of the residue by flash column chromatography (20% MeOH in CHCl_3_) afforded bis-1,2,3-triazole **13e** as a colourless paste (21 mg, 86%): *R*_f_ = 0.25 (20% MeOH in CHCl_3_); IR (KBr) *ν* 3340m br (O–H), 2917s, 2850s, 1632m (C=O), 1549w, 1467m, 1349m, 1300w, 1232w, 1138m, 1082s, 1039s, 948m, 786m, 719s; ^1^H NMR (300 MHz, CDCl_3_/CD_3_OD, 2:1) *δ* 0.85 (app. t, 12H, *J* 6.9 Hz), 1.14–1.49 (stack, 136H), 1.43–1.67 (stack, 8H), 2.14 (app. t, 4H, *J* 7.2 Hz), 3.36–3.52 (stack, 8H), 3.60–3.68 (stack, 32H), 3.68–3.75 (m, 2H), 3.76–3.83 (stack, 4H), 4.01–4.09 (m, 2H), 4.17 (app. t, 2H, *J* 6.2 Hz), 4.52–4.63 (stack, 8H), 4.85 (d, 2H, *J* 2.4 Hz), 7.51 (d, 2H, *J* 8.1 Hz), 7.83 (s, 2H), alcoholic protons not observed; ^13^C NMR (100 MHz, CDCl_3_/CD_3_OD, 2:1) *δ* 13.3 (CH_3_), [22.1, 25.4, 28.8, 28.89, 28.94, 29.06, 29.13, 29.2, 29.3, 31.4, 31.9, 35.9 (CH_2_, overlapping alkyl chain resonances)], 49.7 (CH), 50.5 (CH_2_), 63.6 (CH_2_), 66.6 (CH_2_), 68.2 (CH), 68.9 (CH), 69.0 (CH), 69.4 (CH), [69.6, 69.7 (CH_2_, overlapping ethylene glycol resonances)] 71.4 (CH), 73.9 (CH), 99.1 (CH), 124.3 (CH), 144.8 (quat. C), 173.9 (quat. C); *m*/*z* (TOF ES^+^) 2211.6 ([M+H]^+^, 45%), 694.6 (35, [ceramide]^+^), 450.2 (100).

#### 4.4.12 Bis-1,2,3-triazole **14a** (–(CH_2_)_4−_ link)

Azide **8** (20 mg, 0.023 mmol) and diyne **18a** (1.2 mg, 0.011 mmol) were reacted according to Section 4.3.3. After 10 h, work-up and purification of the residue by flash column chromatography (15% MeOH in CHCl_3_) afforded bis-1,2,3-triazole **14a** as a colourless paste (16 mg, 81%): *R*_f_ = 0.24 (15% MeOH in CHCl_3_); IR (KBr) *ν* 3359m br (O–H), 2921s, 2849s, 1636m (C=O), 1555m, 1468s, 1351w, 1222w, 1150m, 1057s, 904w, 787m, 724s; ^1^H NMR (300 MHz, CDCl_3_/CD_3_OD, 2:1) *δ* 0.84 (app. t, 12H, *J* 6.7 Hz), 1.14–1.38 (stack, 136H), 1.45–1.72 (stack, 12H), 2.14 (app. t, 4H, *J* 7.5 Hz), 2.63–2.74 (m, 4H, *J* 7.2 Hz), 3.38 (dd, 2H, *J* 10.6, 4.17 Hz), 3.43–3.58 (stack, 6H), 3.66–3.72 (m, 2H), 3.73–3.82 (stack, 4H), 4.07–4.20 (stack, 4H), 4.48–4.57 (stack, 4H), 4.85 (d, 2H, *J* 3.6 Hz), 7.41 (d, 2H, *J* 8.8 Hz), 7.56 (s, 2H), alcoholic protons not observed; ^13^C NMR (75 MHz, CDCl_3_/CD_3_OD, 2:1) *δ* 13.4 (CH_3_), [22.2, 24.5, 25.3, 25.4, 25.5, 28.2, 28.9, 28.96, 29.02, 29.2, 29.3, 29.4, 31.5, 32.2, 36.0 (CH_2_, overlapping alkyl chain resonances)], 49.7 (CH), 50.2 (CH_2_), 66.8 (CH_2_), 68.2 (CH), 68.8 (CH), 69.0 (CH), 69.5 (CH), 71.5 (CH), 74.3 (CH), 99.2 (CH), 122.6 (CH), 147.5 (quat. C), 174.0 (quat. C); *m*/*z* (TOF ES^+^) 1894.5 ([M+Na]^+^, 100%).

#### 4.4.13 Bis-1,2,3-triazole **14b** (–(CH_2_)_12−_ link)

Azide **8** (20 mg, 0.023 mmol) and diyne **18b** (2.1 mg, 0.011 mmol) were reacted according to Section 4.3.3. After 10 h, work-up and purification of the residue by flash column chromatography (15% MeOH in CHCl_3_) afforded bis-1,2,3-triazole **14b** as a colourless paste (19 mg, 88%): *R*_f_ = 0.26 (15% MeOH in CHCl_3_); IR (KBr) *ν* 3351m br (O–H), 2917s, 2850s, 1635m (C=O), 1550m, 1467s, 1344w, 1221w, 1150m, 1058s, 906w, 784m, 720m; ^1^H NMR (300 MHz, CDCl_3_/CD_3_OD, 2:1) *δ* 0.85 (app. t, 12H, *J* 6.6 Hz), 1.15–1.49 (stack, 152H), 1.47–1.68 (stack, 12H), 2.14 (app. t, 4H, *J* 7.5 Hz), 2.65 (app. t, 4H, *J* 7.2 Hz), 3.36–3.43 (m, 2H), 3.44–3.55 (stack, 6H), 3.67–3.74 (m, 2H), 3.76–3.83 (stack, 4H), 4.07–4.21 (stack, 4H), 4.47–4.60 (stack, 4H), 4.86 (d, 2H, *J* 3.3 Hz), 7.56 (s, 2H), exchangeable protons not observed; ^13^C NMR (100 MHz, CDCl_3_/CD_3_OD, 2:1) *δ* 13.1 (CH_3_), [22.0, 24.8, 25.2, 25.3, 28.7, 28.76, 28.83, 29.0, 29.1, 29.2, 31.3, 35.8 (CH_2_, overlapping alkyl chain resonances)], 49.5 (CH), 50.2 (CH_2_), 66.4 (CH_2_), 68.1 (CH), 68.9 (CH), 69.0 (CH), 69.4 (CH), 71.3 (CH), 74.0 (CH), 99.0 (CH), 122.3 (CH), 147.6 (quat. C), 174.0 (quat. C); *m*/*z* (TOF ES^−^) 1983.1 ([M−H]^−^ 100%).

#### 4.4.14 Bis-1,2,3-triazole **14c** (–(CH_2_)_18−_ link)

Azide **8** (20 mg, 0.023 mmol) and diyne **18c** (3.3 mg, 0.011 mmol) were reacted according to Section 4.3.3. After 10 h, work-up and purification of the residue by flash column chromatography (15% MeOH in CHCl_3_) afforded bis-1,2,3-triazole **14c** as a colourless paste (20 mg, 87%): *R*_f_ = 0.27 (15% MeOH in CHCl_3_); IR (film) *ν* 3386m br (O–H), 2918s, 2851s, 1637m (C=O), 1537w, 1467s, 1438w, 1345w, 1204m, 1151s, 1065s, 1036s, 801m, 720s; ^1^H NMR (300 MHz, CDCl_3_/CD_3_OD, 2:1) *δ* 0.85 (app. t, 12H, *J* 6.6 Hz), 1.14–1.49 (stack, 164H), 1.47–1.68 (stack, 12H), 2.14 (app. t, 4H, *J* 7.7 Hz), 2.65 (app. t, 4H, *J* 7.5 Hz), 3.38 (dd, 2H, *J* 10.2, 4.2 Hz), 3.43–3.55 (stack, 6H), 3.69 (dd, 2H, *J* 10.2, 3.0 Hz), 3.76–3.82 (stack, 4H), 4.07–4.20 (stack, 4H), 4.45–4.60 (stack, 4H), 4.87 (d, 2H, *J* 3.6 Hz), 7.54 (br s, 2H), exchangeable protons not observed; ^13^C NMR (100 MHz, CDCl_3_/CD_3_OD, 2:1) *δ* 13.2 (CH_3_), [22.1, 24.8, 25.28, 25.32, 28.77, 28.82, 28.87, 28.92, 29.00, 29.09, 29.13, 29.3, 31.4, 32.0, 35.8 (CH_2_, overlapping alkyl resonances)], 49.6 (CH), 50.2 (CH_2_), 66.4 (CH_2_), 68.1 (CH), 68.9 (CH), 69.0 (CH), 69.4 (CH), 71.3 (CH), 74.1 (CH), 99.1 (CH), 122.2 (CH), 147.7 (quat. C), 173.8 (quat. C); *m*/*z* (TOF ES^+^) 2067.7 ([M+Na]^+^, 30%), 1885.3 (30), 694.6 (100, [ceramide]^+^).

#### 4.4.15 *O*-Propargyl-*O*′-methyl-di(ethylene glycol) (**9b**)

Alkyne **9b** was prepared from di(ethylene glycol) monomethyl ether (**19a**) (0.50 g, 4.16 mmol) and propargyl bromide (0.53 mL of a 80% w/w solution in toluene, 6.24 mmol) according to Section 4.3.1. After stirring overnight, removal of the solvent and purification of the residue by flash column chromatography (25% EtOAc in hexane) afforded alkyne **9b** as a yellow oil (0.57 g, 87%): *R*_f_ = 0.26 (25% EtOAc in hexane); IR (film) *ν* 3251m (≡C–H), 2866m, 2113w (C≡C), 1442m, 1350m, 1295w, 1288w, 1136s, 1094s, 1033m, 918m, 877w, 843m, 734m; ^1^H NMR (300 MHz, CDCl_3_) *δ* 2.41 (t, 1H, *J* 2.4 Hz), 3.35 (s, 3H), 3.50–3.58 (m, 2H), 3.59–3.72 (stack, 6H), 4.17 (d, 2H, *J* 2.4 Hz); ^13^C NMR (75 MHz, CDCl_3_) *δ* 58.3 (CH), 58.9 (CH_3_), 69.0 (CH_2_), 70.3 (CH_2_), 70.5 (CH_2_), 71.8 (CH_2_), 74.4 (CH), 79.6 (quat. C); *m*/*z* (TOF ES^+^) 181.1 ([M+Na]^+^, 100%); HRMS *m*/*z* (TOF ES^+^) 181.0837. C_10_H_18_NaO_3_ requires 181.0841.

#### 4.4.16 *O*-Propargyl-*O*′-methyl-triethylene glycol (**9c**)

Alkyne **9c** was prepared from tri(ethylene glycol) monomethyl ether (**19b**) (250 mg, 1.52 mmol) and propargyl bromide (190 μL of a 80% w/w solution in toluene, 2.28 mmol) according to Section 4.3.1. After stirring overnight, removal of the solvent and purification of the residue by flash column chromatography (40% EtOAc in hexane) afforded alkyne **9c** as a yellow oil (279 mg, 91%): *R*_f_ = 0.30 (40% EtOAc in hexane); IR (film) *ν* 3248m (≡C–H), 2865m, 2114w (C≡C), 1442m, 1348m, 1246m, 1092s, 1031s, 918s, 879s, 842s, 756m, 731s; ^1^H NMR (300 MHz, CDCl_3_) *δ* 2.41 (t, 1H, *J* 2.4 Hz), 3.35 (s, 3H), 3.50–3.59 (m, 2H), 3.59–3.74 (stack, 10H), 4.17 (d, 2H, *J* 2.4 Hz); ^13^C NMR (75 MHz, CDCl_3_) *δ* 58.3 (CH), 58.9 (CH_3_), 69.0 (CH_2_), 70.3 (CH_2_), 70.4 (CH_2_), 70.5 (CH_2_, overlapping ethylene glycol resonances), 71.8 (CH_2_), 74.4 (CH), 79.6 (quat. C); *m*/*z* (TOF ES^+^) 225.1 ([M+Na]^+^, 100%); HRMS *m*/*z* (TOF ES^+^) 225.1110. C_10_H_18_NaO_4_ requires 225.1103.

#### 4.4.17 *O*-Propargyl-*O*′-methyl-penta(ethylene glycol) (**9d**)

Alkyne **9d** was prepared from penta(ethylene glycol) monomethyl ether (**19c**) (250 mg, 0.99 mmol) and propargyl bromide (126 μL of a 80% w/w solution in toluene, 1.49 mmol) according to Section 4.3.1. After stirring overnight, removal of the solvent and purification of the residue by flash column chromatography (60% EtOAc in hexane) afforded alkyne **9d** as a yellow oil (244 mg, 85%): *R*_f_ = 0.24 (60% EtOAc in hexane); IR (film) *ν* 3253m (≡C–H), 2869m, 2113w (C≡C), 1447m, 1346m, 1247m, 1093s, 1029s, 917s, 879s, 842s, 748m, 731s; ^1^H NMR (300 MHz, CDCl_3_) *δ* 2.42 (t, 1H, *J* 2.4 Hz), 3.36 (s, 3H), 3.50–3.58 (m, 2H), 3.60–3.73 (stack, 18H), 4.18 (d, 2H, *J* 2.4 Hz); ^13^C NMR (75 MHz, CDCl_3_) *δ* 58.3 (CH), 59.0 (CH_3_), 69.0 (CH_2_), 70.3 (CH_2_), 70.45 (CH_2_), 70.51 (CH_2_, overlapping ethylene glycol resonances), 71.9 (CH_2_), 74.4 (CH), 79.6 (quat. C); *m*/*z* (TOF ES^+^) 313.3 ([M+Na]^+^, 100%); HRMS *m*/*z* (TOF ES^+^) 313.1624. C_14_H_26_NaO_6_ requires 313.1627.

#### 4.4.18 *O*-Propargyl-*O*′-methyl-octa(ethylene glycol) (**9e**)

Alkyne **9e** was prepared from octa(ethylene glycol) monomethyl ether (**19d**) (100 mg, 0.26 mmol) and propargyl bromide (32 μL of a 80% w/w solution in toluene, 0.39 mmol) according to Section 4.3.1. After stirring overnight, removal of the solvent and purification of the residue by flash column chromatography (5% MeOH in CHCl_3_) afforded alkyne **9e** as a yellow oil (244 mg, 85%): *R*_f_ = 0.23 (5% MeOH in CHCl_3_); IR (film) *ν* 3248m (≡C–H), 2916m, 2864m, 2114w (C≡C), 1956w, 1457m, 1346m, 1292m, 1246m, 1094s, 1030m, 946m, 839m, 764m, 703m; ^1^H NMR (300 MHz, CDCl_3_) *δ* 2.41 (t, 1H, *J* 2.4 Hz), 3.32 (s, 3H), 3.46–3.54 (m, 2H), 3.57–3.70 (stack, 30H), 4.15 (d, 2H, *J* 2.4 Hz); ^13^C NMR (75 MHz, CDCl_3_) *δ* 58.2 (CH), 58.8 (CH_3_), 68.9 (CH_2_), 70.2 (CH_2_), 70.4 (CH_2_, some overlapping ethylene glycol resonances), 71.8 (CH_2_), 74.4 (CH), 79.5 (quat. C); *m*/*z* (TOF ES^+^) 445.2 ([M+Na]^+^, 100%); HRMS *m*/*z* (TOF ES^+^) 445.2421. C_20_H_38_NaO_9_ requires 445.2414.

#### 4.4.19 1,2,3-triazole **10a** (PEG-0 tail)

Azide **8** (20 mg, 0.023 mmol) and alkyne **9a** (1.6 mg, 0.023 mmol) were reacted according to Section 4.3.3. After 10 h, work-up and purification of the residue by flash column chromatography (15% MeOH in CHCl_3_) afforded 1,2,3-triazole **10a** as a colourless paste (20 mg, 92%): *R*_f_ = 0.29 (15% MeOH in CHCl_3_); IR (KBr) *ν* 3352br s (O–H), 2914s, 2851s, 1638m (C=O), 1543w, 1467m, 1343w, 1229w, 1150m, 1067s, 1034s, 784w, 720s, 717w, 668m; ^1^H NMR (300 MHz, CDCl_3_/CD_3_OD, 2:1) *δ* 0.84 (app. t, 6H, *J* 6.6 Hz), 1.14–1.38 (stack, 68H), 1.43–1.67 (stack, 4H), 2.13 (app. t, 2H, *J* 7.5 Hz), 3.33–3.40 (stack including [3.37 (s, 3H)], 4H), 3.42–3.52 (stack, 3H), 3.69 (dd, 1H, *J* 9.8, 3.3 Hz), 3.74–3.83 (stack, 2H), 4.03–4.13 (m, 1H), 4.13–4.20 (m, 1H), 4.52 (s, 2H), 4.53–4.59 (m, 2H), 4.85 (d, 1H, *J* 3.7 Hz), 7.28 (d, 1H, *J* 8.79 Hz), 7.78 (s, 1H), alcoholic protons not observed; ^13^C NMR (75 MHz, CDCl_3_/CD_3_OD, 2:1) *δ* 14.2 (CH_3_), [23.0, 26.2, 29.7, 30.0, 32.3, 33.0, 36.8 (CH_2_, overlapping alkyl resonances)], 50.5 (CH), 51.3 (CH_2_), 58.4 (CH_3_), 65.8 (CH_2_), 67.5 (CH_2_), 69.0 (CH), 69.7 (CH), 69.8 (CH), 70.3 (CH), 72.3 (CH), 75.1 (CH), 100.0 (CH), 125.0 (CH), 144.8 (quat. C), 174.7 (quat. C); *m*/*z* (TOF ES^+^) 975.8 ([M+Na]^+^, 100%); HRMS *m*/*z* (TOF ES^+^) 975.7711. C_54_H_104_N_4_NaO_9_ requires 975.7701.

#### 4.4.20 1,2,3-triazole **10b** (PEG-2 tail)

Azide **8** (20 mg, 0.023 mmol) and alkyne **9b** (4.2 mg, 0.023 mmol) were reacted according to Section 4.3.3. After 10 h, work-up and purification of the residue by flash column chromatography (15% MeOH in CHCl_3_) afforded 1,2,3-triazole **10b** as a colourless paste (22 mg, 94%): *R*_f_ = 0.27 (15% MeOH in CHCl_3_); IR (KBr) *ν* 3347m br (O–H), 2917s, 2849s, 1632m (C=O), 1544w, 1469m, 1349w, 1347w, 1230w, 1141m, 1063s, 1036s, 785w, 732m, 720s; ^1^H NMR (300 MHz, CDCl_3_/CD_3_OD, 2:1) *δ* 0.84 (app. t, 6H, *J* 6.6 Hz), 1.13–1.38 (stack, 68H), 1.46–1.63 (stack, 4H), 2.13 (app. t, 2H, *J* 7.4 Hz), 3.33–3.39 (stack including [3.35 (s, 3H)]), 4H, 3.42–3.51 (stack, 3H), 3.51–3.55 (stack, 2H), 3.58–3.64 (stack, 6H), 3.69 (dd, 1H, *J* 9.6, 3.2 Hz), 3.76–3.80 (stack, 2H), 4.09 (dd, 1H, *J* 8.4, 4.8 Hz), 4.15–4.20 (m, 1H), 4.52–4.58 (m, 2H), 4.62 (s, 2H), 4.85 (d, 1H, *J* 4.0 Hz), 7.80 (s, 1H), exchangeable protons not observed; ^13^C NMR (75 MHz, CDCl_3_/CD_3_OD, 2:1) *δ* 14.2 (CH_3_), [23.0, 26.2, 29.7, 30.0, 32.3, 33.0, 36.8 (CH_2_, overlapping alkyl resonances)], 50.5 (CH), 51.3 (CH_2_), 59.1 (CH_3_), 64.5 (CH_2_), 67.5 (CH_2_), 69.0 (CH), 69.7 (CH), 69.8 (CH), 70.0 (CH_2_), 70.3 (CH), 70.67 (CH_2_), 70.73 (CH_2_), 72.2 (CH_2_), 72.3 (CH), 75.0 (CH), 100.0 (CH), 125.1 (CH), 145.0 (quat. C), 174.7 (quat. C); *m*/*z* (TOF ES^+^) 1064.0 ([M+Na]^+^, 100%); HRMS *m*/*z* (TOF ES^+^) 1063.8217. C_58_H_112_N_4_NaO_11_ requires 1063.8225.

#### 4.4.21 1,2,3-Triazole **10c** (PEG-3 tail)

Azide **8** (20 mg, 0.023 mmol) and alkyne **9c** (4.6 mg, 0.023 mmol) were reacted according to Section 4.3.3. After 10 h, work-up and purification of the residue by flash column chromatography (15% MeOH in CHCl_3_) afforded 1,2,3-triazole **10c** as a colourless paste (23 mg, 91%): *R*_f_ = 0.27 (15% MeOH in CHCl_3_); IR (KBr) *ν* 3368m br (O–H), 2916s, 2849s, 1630m (C=O), 1551w, 1467s, 1349w, 1232w, 1147m, 1071s, 1062w, 1035s, 786w, 720m; ^1^H NMR (300 MHz, CDCl_3_/CD_3_OD, 2:1) *δ* 0.86 (app. t, 6H, *J* 6.6 Hz), 1.12–1.39 (stack, 68H), 1.44–1.67 (stack, 4H), 2.13 (app. t, 2H, *J* 7.5 Hz), 3.33–3.41 (stack including [3.36 (s, 3H)]), 4H, 3.42–3.50 (stack, 3H), 3.50–3.56 (stack, 2H), 3.58–3.64 (stack, 10H), 3.69 (dd, 1H, *J* 9.6, 3.3 Hz), 3.75–3.81 (stack, 2H), 4.04–4.12 (m, 1H), 4.17 (app. t, 1H, *J* 6.9 Hz), 4.53–4.59 (m, 2H), 4.62 (s, 2H), 4.85 (d, 1H, *J* 3.9 Hz), 7.31 (d, 1H, *J* 9.0 Hz), 7.80 (s, 1H), alcoholic protons not observed; ^13^C NMR (75 MHz, CDCl_3_/CD_3_OD, 2:1) *δ* 14.2 (CH_3_), [23.0, 26.2, 29.7, 29.8, 30.1, 32.3, 33.0, 36.8 (CH_2_, some overlapping alkyl resonances)], 50.5 (CH), 51.3 (CH_2_), 59.1 (CH_3_), 64.6 (CH_2_), 67.5 (CH_2_), 69.0 (CH), 69.7 (CH), 69.8 (CH), 70.0 (CH_2_), 70.3 (CH), [70.67, 70.74, 70.8 (CH_2_, some overlapping ethylene glycol resonances)], 72.2 (CH_2_), 72.3 (CH), 75.0 (CH), 100.0 (CH), 125.1 (CH), 145.0 (quat. C), 174.7 (quat. C); *m*/*z* (TOF ES^+^) 1107.9 ([M+Na]^+^, 100%); HRMS *m*/*z* (TOF ES^+^) 1107.8484. C_60_H_116_N_4_NaO_12_ requires 1107.8487.

#### 4.4.22 1,2,3-Triazole **10d** (PEG-5 tail)

Azide **8** (20 mg, 0.023 mmol) and alkyne **9d** (6.7 mg, 0.023 mmol) were reacted according to Section 4.3.3. After 10 h, work-up and purification of the residue by flash column chromatography (20% MeOH in CHCl_3_) afforded 1,2,3-triazole **10d** as a colourless paste (20 mg, 87%): *R*_f_ = 0.28 (20% MeOH in CHCl_3_); IR (KBr) *ν* 3368m br (O–H), 2917s, 2848s, 1635m (C=O), 1550w, 1467s, 1348w, 1231w, 1145m, 1070s, 1035s, 1001w, 785w, 719m, 685m; ^1^H NMR (300 MHz, CDCl_3_/CD_3_OD, 2:1) *δ* 0.84 (app. t, 6H, *J* 6.5 Hz), 1.13–1.39 (stack, 68H), 1.44–1.68 (stack, 4H), 2.13 (app. t, 2H, *J* 7.4 Hz), 3.33–3.41 (stack including [3.35 (s, 3H)], 4H), 3.42–3.50 (stack, 3H), 3.50–3.55 (stack, 2H), 3.58–3.65 (stack, 18H), 3.69 (dd, 1H, *J* 9.6, 3.2 Hz), 3.74–3.82 (stack, 2H), 4.04–4.12 (m, 1H), 4.17 (app. t, 1H, *J* 6.7 Hz), 4.52–4.58 (m, 2H), 4.62 (s, 2H), 4.85 (d, 1H, *J* 3.6 Hz), 7.80 (s, 1H), exchangeable protons not observed; ^13^C NMR (75 MHz, CDCl_3_/CD_3_OD, 2:1) *δ* 14.3 (CH_3_), [23.0, 26.2, 29.7, 29.8, 30.1, 32.3, 32.9, 36.8 (CH_2_, some overlapping alkyl resonances)], 50.5 (CH), 51.3 (CH_2_), 59.1 (CH_3_), 64.6 (CH_2_), 67.5 (CH_2_), 69.0 (CH), 69.7 (CH), 69.8 (CH), 70.0 (CH_2_), 70.3 (CH), [70.66, 70.79 (CH_2_, some overlapping ethylene glycol resonances)], 72.2 (CH_2_), 72.3 (CH), 75.0 (CH), 100.0 (CH), 125.1 (CH), 145.0 (quat. C), 174.7 (quat. C); *m*/*z* (TOF ES^+^) 1196.6 ([M+Na]^+^, 100%); HRMS *m*/*z* (TOF ES^+^) 1195.9045. C_64_H_124_N_4_NaO_14_ requires 1195.9045.

#### 4.4.23 1,2,3-Triazole **10e** (PEG-8 tail)

Azide **8** (20 mg, 0.023 mmol) and alkyne **9e** (9.7 mg, 0.023 mmol) were reacted according to Section 4.3.3. After 10 h, work-up and purification of the residue by flash column chromatography (20% MeOH in CHCl_3_) afforded 1,2,3-triazole **10e** as a colourless paste (29 mg, 97%): *R*_f_ = 0.27 (20% MeOH in CHCl_3_); IR (Kr) *ν* 3336m br (O–H), 2919s, 2849s, 1630m (C=O), 1549w, 1467m, 1350m, 1301w, 1231w, 1138m, 1082s, 1039s, 1012w, 948m, 786m, 719s; ^1^H NMR (300 MHz, CDCl_3_/CD_3_OD, 2:1) *δ* 0.84 (app. t, 6H, *J* 6.6 Hz), 1.12–1.39 (stack, 68H), 1.45–1.69 (stack, 4H), 2.13 (app. t, 2H, *J* 7.4 Hz), 3.33–3.41 (stack including [3.35 (s, 3H)], 4H), 3.41–3.50 (stack, 3H), 3.50–3.56 (stack, 2H), 3.58–3.65 (stack, 30H), 3.69 (dd, 1H, *J* 10.2, 3.0 Hz), 3.74–3.81 (stack, 2H), 4.05–4.13 (m, 1H), 4.18 (app. t, 1H, *J* 6.6 Hz), 4.52–4.58 (m, 2H), 4.62 (s, 2H), 4.85 (d, 1H, *J* 3.6 Hz), 7.28 (d, 1H, *J* 8.7 Hz), 7.80 (s, 1H), alcoholic protons not observed; ^13^C NMR (75 MHz, CDCl_3_/CD_3_OD, 2:1) *δ* 14.2 (CH_3_), [23.0, 26.2, 29.7, 30.0, 32.3, 33.0, 36.8 (CH_2_, some overlapping alkyl resonances)], 50.4 (CH), 51.2 (CH_2_), 59.1 (CH_3_), 64.5 (CH_2_), 67.5 (CH_2_), 69.0 (CH), 69.7 (CH), 69.8 (CH), 70.0 (CH_2_), 70.3 (CH), 70.8 (CH_2_, some overlapping ethylene glycol resonances), 72.2 (CH_2_), 72.3 (CH), 75.0 (CH), 100.0 (CH), 125.1 (CH), 145.0 (quat. C), 174.7 (quat. C); *m*/*z* (TOF ES^+^) 1328.8 ([M+Na]^+^, 100%); HRMS *m*/*z* (TOF ES^+^) 1327.9788. C_70_H_136_N_4_NaO_17_ requires 1327.9798.

#### 4.4.24 1,2,3-Triazole **12** (octyl tail)

Azide **8** (20 mg, 0.023 mmol) and 1-decyne (**11**) (3.3 mg, 0.023 mmol) were reacted according to Section 4.3.3. After 10 h, work-up and purification of the residue by flash column chromatography (15% MeOH in CHCl_3_) afforded 1,2,3-triazole **12** as a colourless paste (22 mg, 95%): *R*_f_ = 0.30 (15% MeOH in CHCl_3_); IR (film) *ν* 3385m br (O–H), 2916s, 2852s, 1635m (C=O), 1532w, 1468s, 1438w, 1345w, 1204m, 1151s, 1065s, 1036s, 801m, 732m, 725m; ^1^H NMR (300 MHz, CDCl_3_/CD_3_OD, 2:1) *δ* 0.84 (app. t, 9H, *J* 6.6 Hz), 1.12–1.43 (stack, 80H), 1.47–1.71 (stack, 6H), 2.13 (app. t, 2H, *J* 7.7 Hz), 2.64 (app. t, 2H, *J* 7.8 Hz), 3.37 (dd, 1H, *J* 10.5, 4.5 Hz), 3.42–3.55 (stack, 3H), 3.68 (dd, 1H, *J* 10.2, 3.0 Hz), 3.75–3.84 (stack, 2H), 4.07–4.21 (stack, 2H), 4.51–4.57 (m, 2H), 4.86 (d, 1H, *J* 3.6 Hz), 7.29 (d, 1H, *J* 8.7 Hz), 7.52 (s, 1H); ^13^C NMR (75 MHz, CDCl_3_/CD_3_OD, 2:1) *δ* 14.3 (CH_3_), [23.0, 25.8, 26.3, 29.7, 29.8, 30.1, 32.3, 33.0, 36.8 (CH_2_, some overlapping alkyl resonances)], 50.4 (CH), 51.1 (CH_2_), 67.4 (CH_2_), 69.1 (CH), 69.8 (CH), 69.9 (CH), 70.3 (CH), 72.3 (CH), 75.1 (CH), 100.0 (CH), 123.17 (CH), 148.6 (quat. C), 174.7 (quat. C); *m*/*z* (TOF ES^+^) 1043.9 ([M+Na]^+^, 100%); HRMS *m*/*z* (TOF ES^+^) 1043.8697. C_60_H_116_N_4_NaO_8_ requires 1043.8691.

### 4.5 Materials and methods for biology

#### 4.5.1 Mice and reagents

C57BL/6 and CD1d^−/−^ (*i*NKT cell-deficient) mice were used. Animal experiments were carried out under the authority of a UK Home Office Project License. Compounds were solubilised in 150 mM NaCl and 0.5% Tween 20 (vehicle).

#### 4.5.2 In vitro and in vivo activation of *i*NKT cells

For in vitro activation of *i*NKT cells, 5 × 10^5^ splenoctyes from C57BL/6 and CD1d^−/−^ (*i*NKT cell-deficient) mice were pulsed with various concentrations of α-GalCer **7**, **14a**–**c**, **13a**–**e**, **12**, **10a**–**e** or vehicle for 48 h. Supernatants were removed and the presence of IFNγ determined by ELISA.^29^ For in vivo activation of *i*NKT cells, C57 BL/6 WT or CD1d^−/−^ mice were injected intravenously (iv) with 1 μg lipids and blood serum taken at 18 h and the presence of IFNγ determined by ELISA.^33^

#### 4.5.3 Soluble human NKT TCR binding assay

Soluble *i*NKT TCR tetramers were prepared according to the protocol described by McCarthy et al.^60^ C1R-hCD1d cells were pulsed with lipids or vehicle at various concentrations overnight and following washes incubated with fluorescently labelled *i*NKT TCR tetramer. *i*NKT TCR–CD1d–lipid complexes were detected by flow cytometry on a FACScalibur device using CellQuest software.

## Supplementary data

Supplementary data Spectral data.

## Footnotes

† Quoted lengths refer to the linker in its fully extended conformation.
