# Supplementary material for: Towards multivalent CD1d ligands: synthesis and biological activity of homodimeric α-galactosyl ceramide analogues
Source: Carbohydr Res. 2012 Jul 15;356(9-10):152–62. doi: 10.1016/j.carres.2012.02.034 (PMC3413882; doi:10.1016/j.carres.2012.02.034)
Supplement: Supplementary data — Spectral data. [file mmc1.pdf]

Towards Multivalent CD1d Ligands: Synthesis and Biological Activity of Homodimeric α-Galactosyl Ceramide Analogues

Peter J. Jervis *et al.*

Scanned ^{1}H-NMR and ^{13}C-NMR Spectra

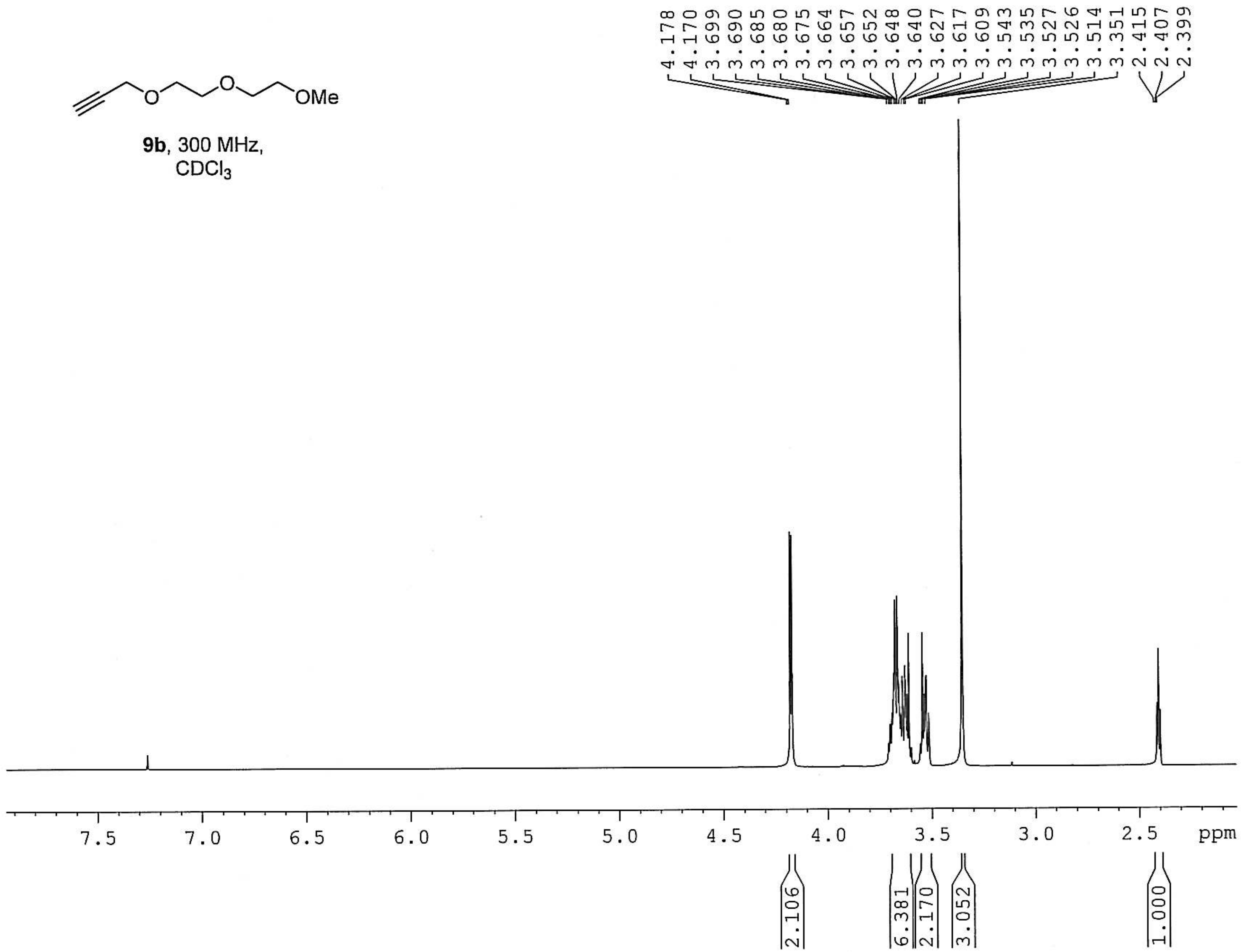

9b, 300 MHz, $CDCl_3$

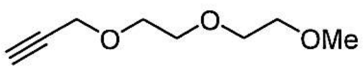

9b, 75 MHz, $CDCl_3$

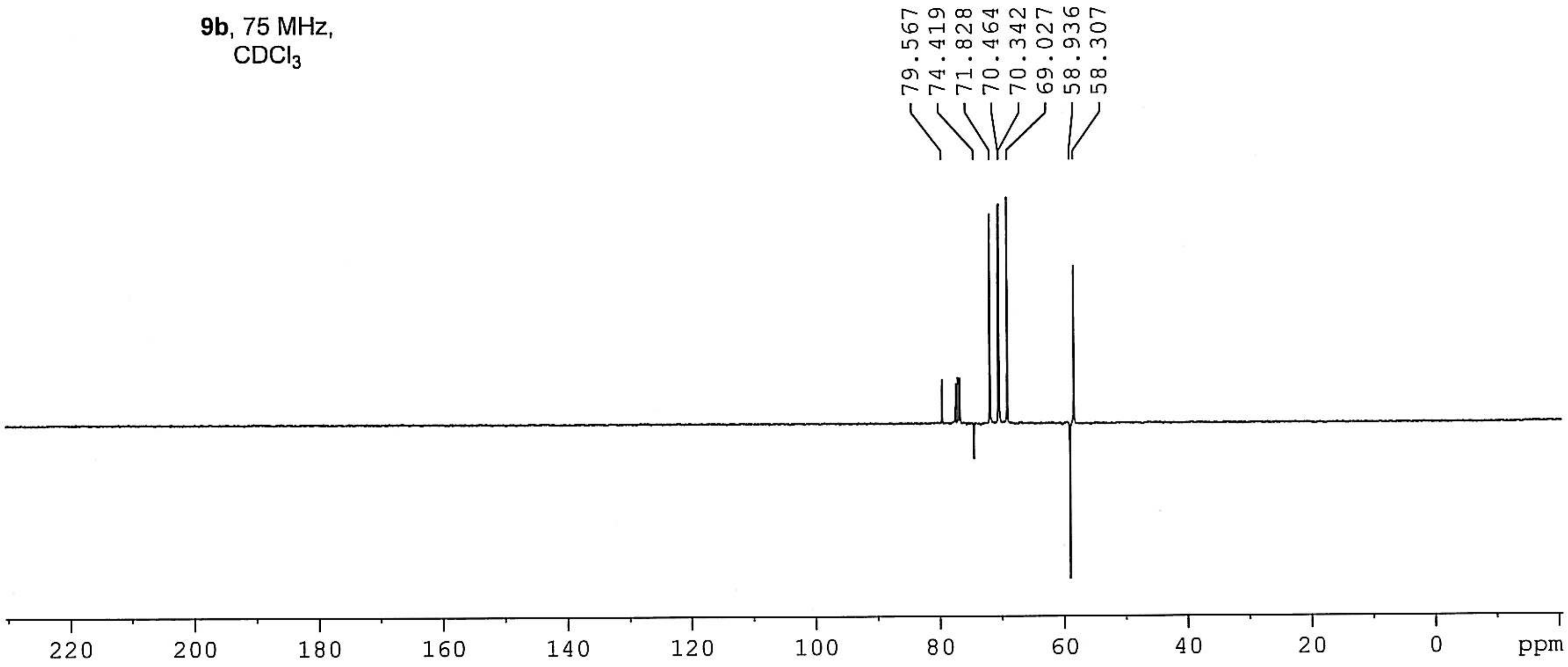

9c, 300 MHz, $CDCl_3$

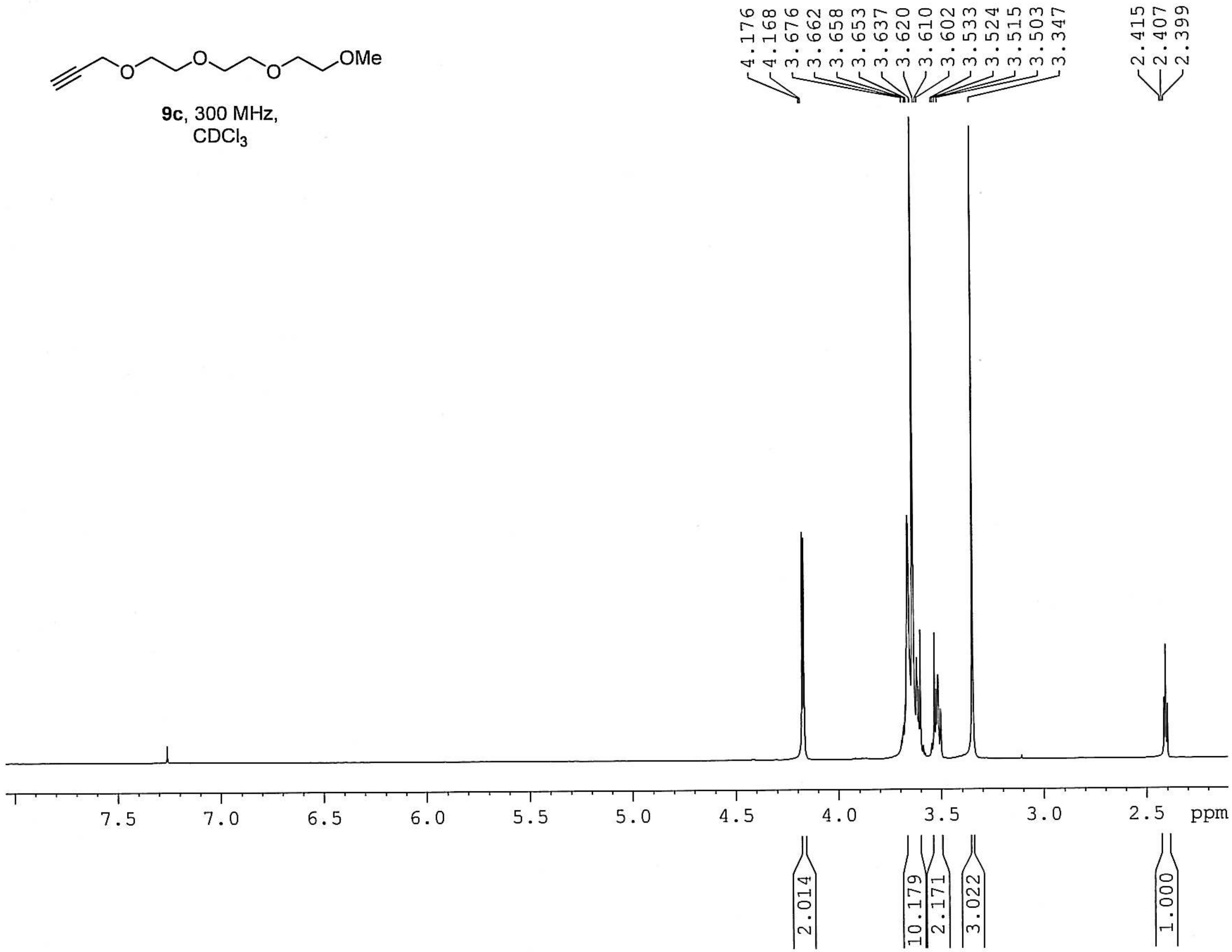

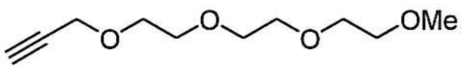

9c, 75 MHz, $CDCl_3$

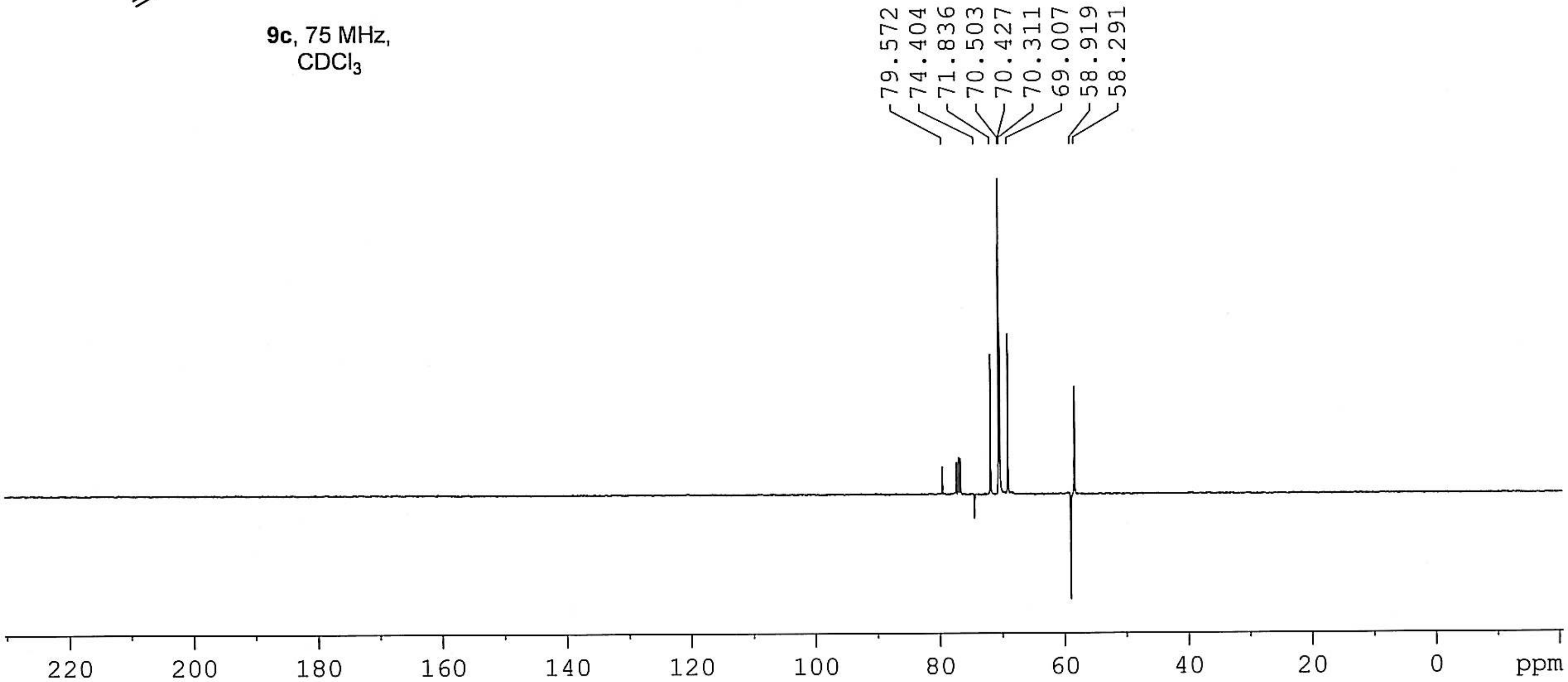

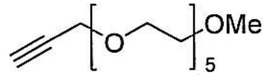

9d, 300 MHz, $CDCl_3$

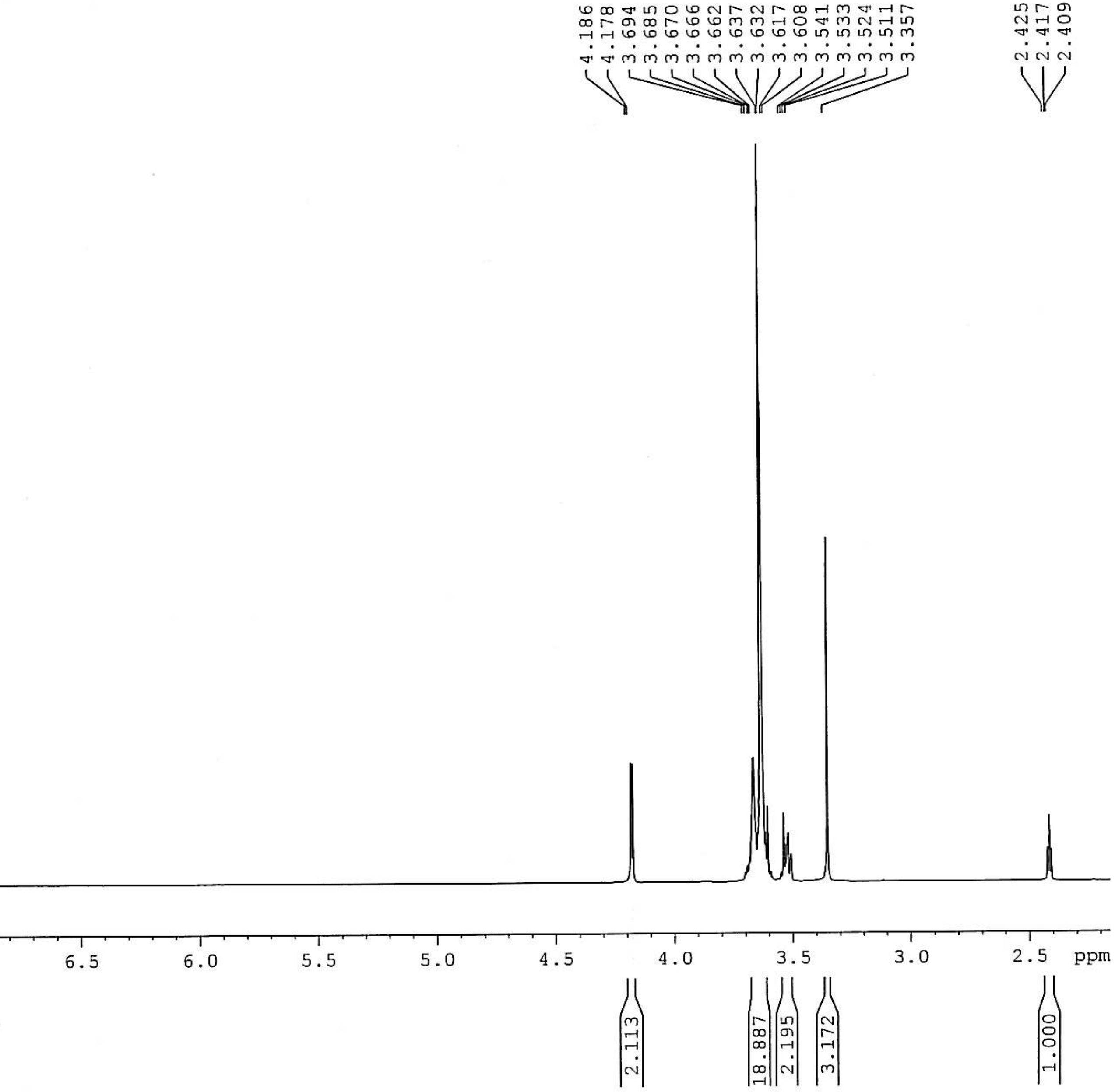

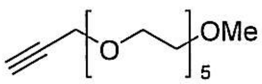

9d, 75 MHz,
$CDCl_3$

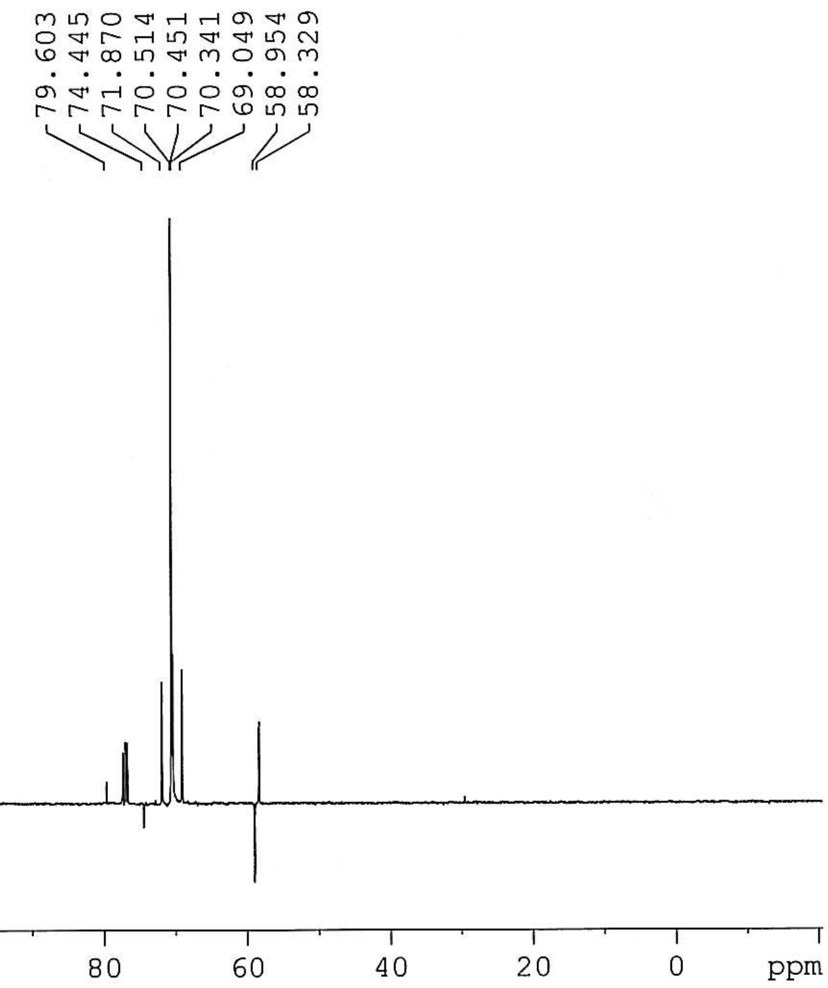

9e, 300 MHz, $CDCl_3$

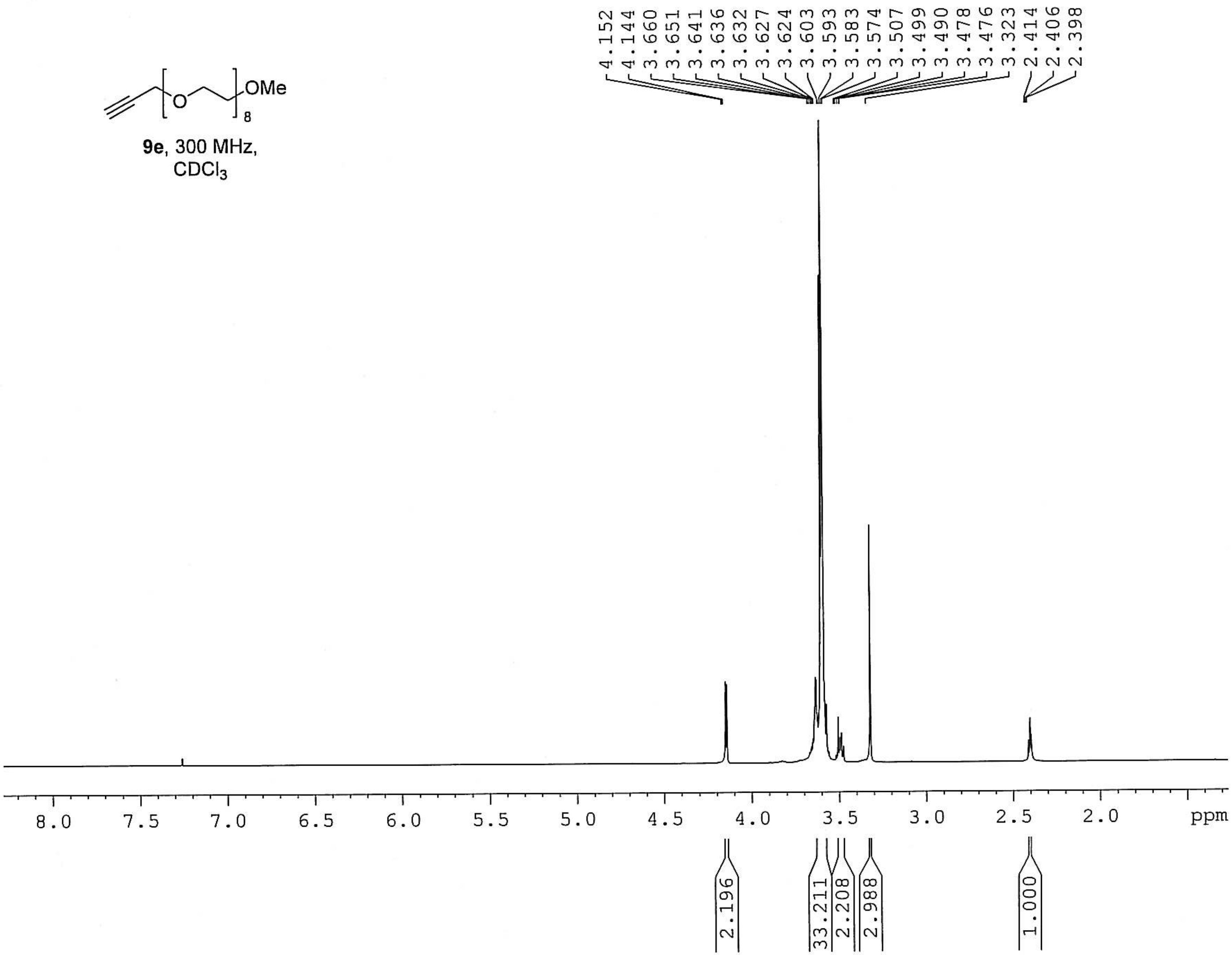

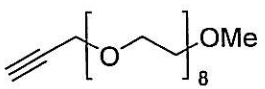

9e, 75 MHz, $CDCl_3$

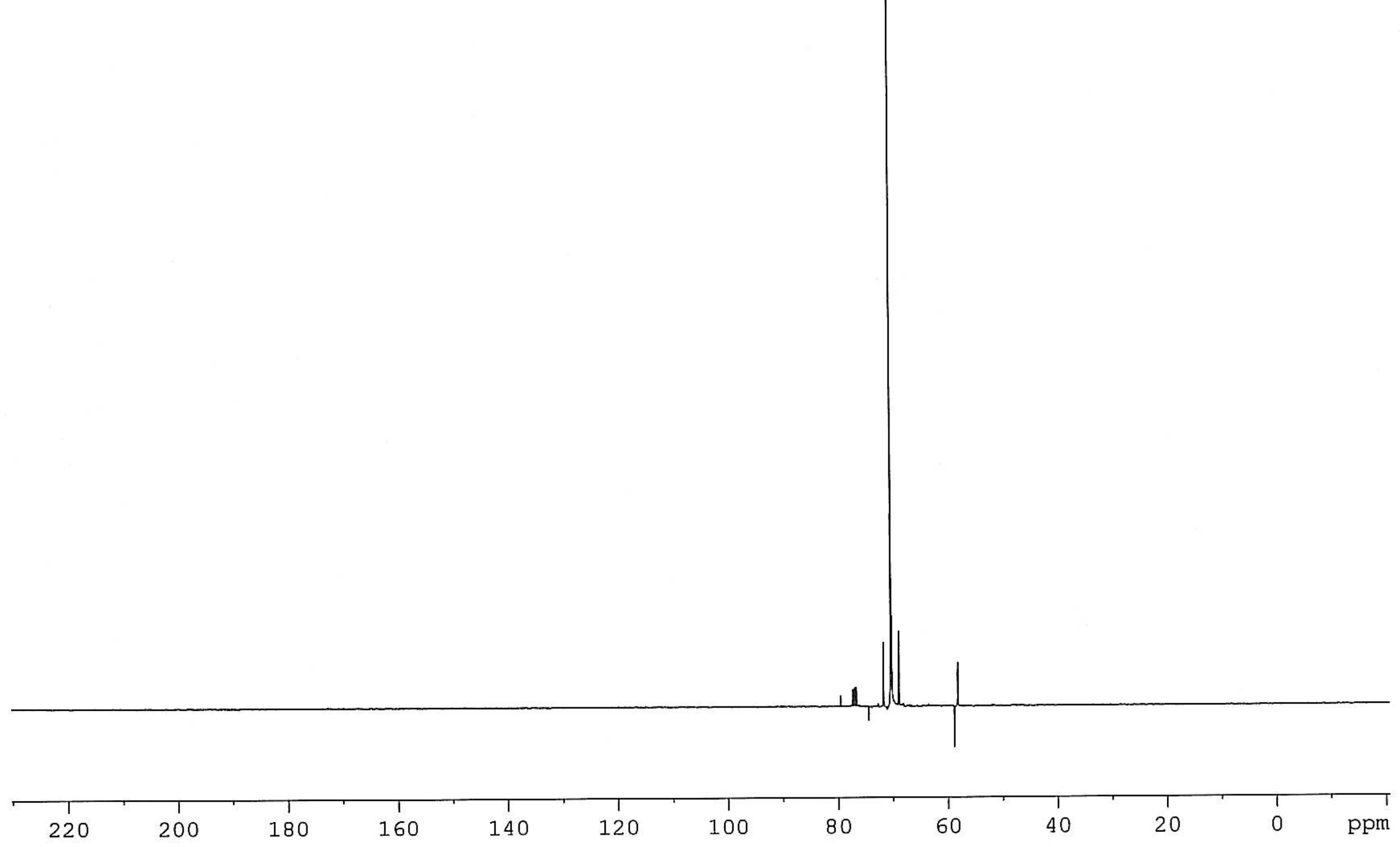

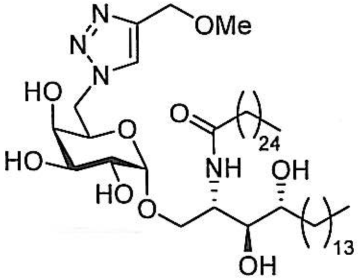

10a, 300 MHz, $CDCl_3/CD_3OD$ 2:1

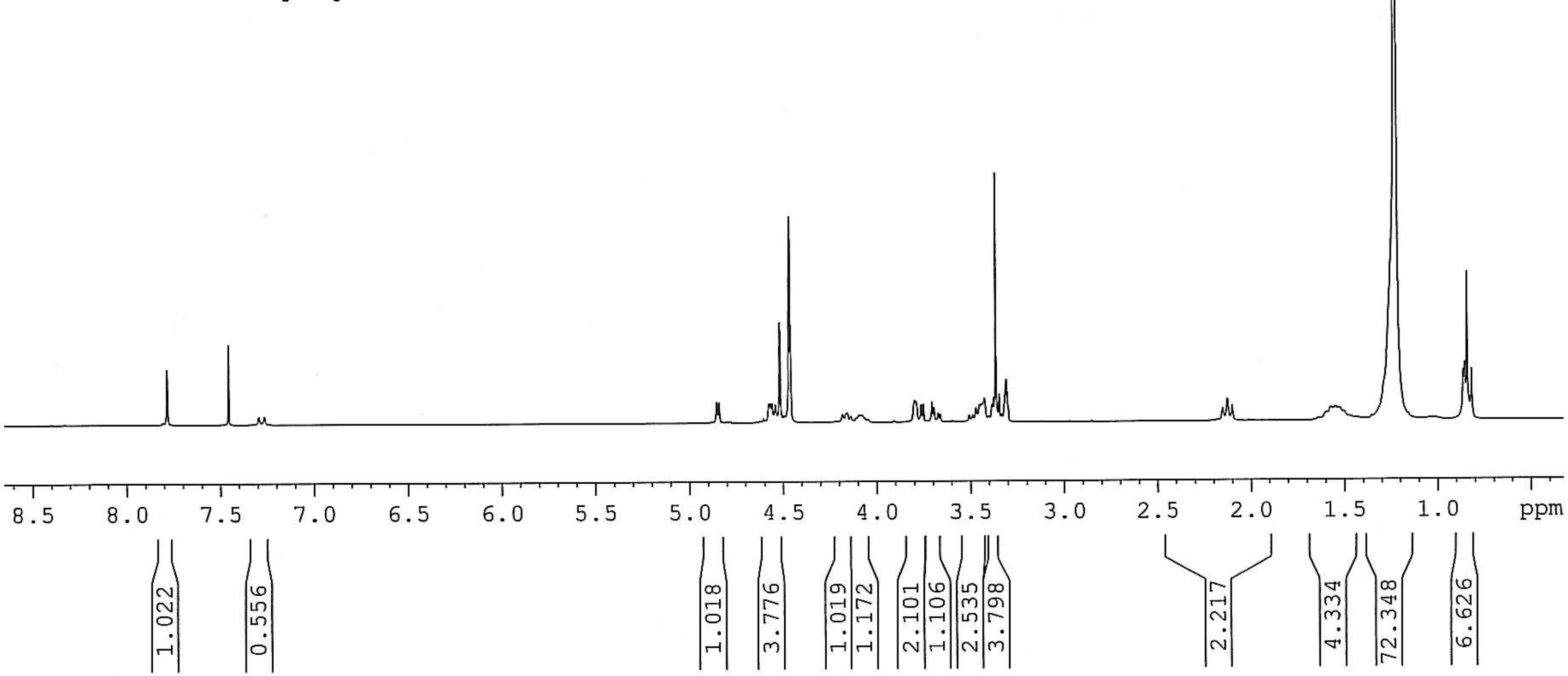

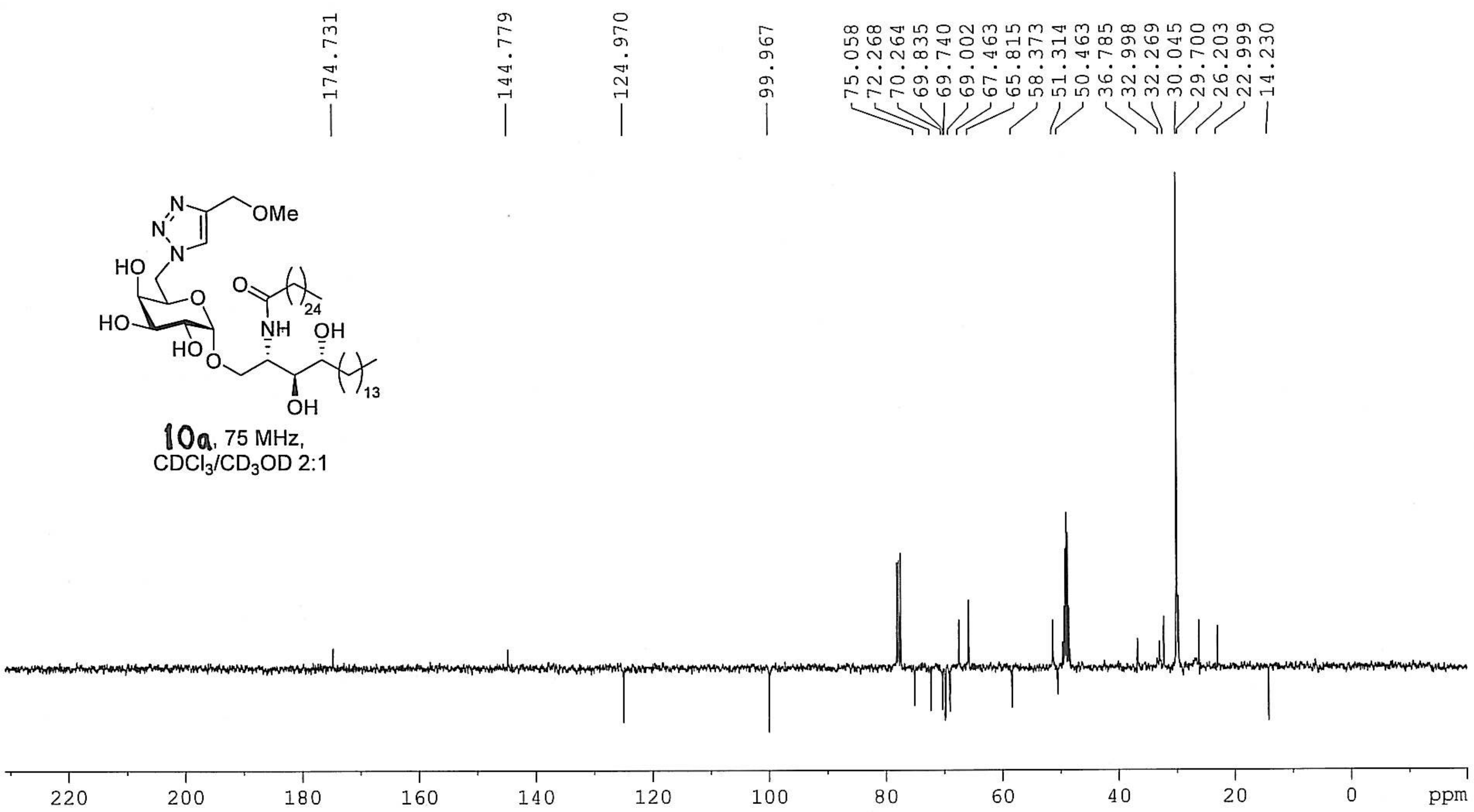

10a, 75 MHz, $CDCl_3/CD_3OD$ 2:1

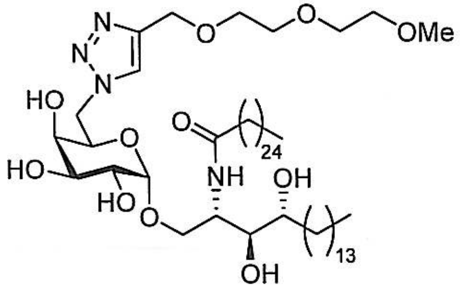

10b, 300 MHz, $CDCl_3/CD_3OD$ 2:1

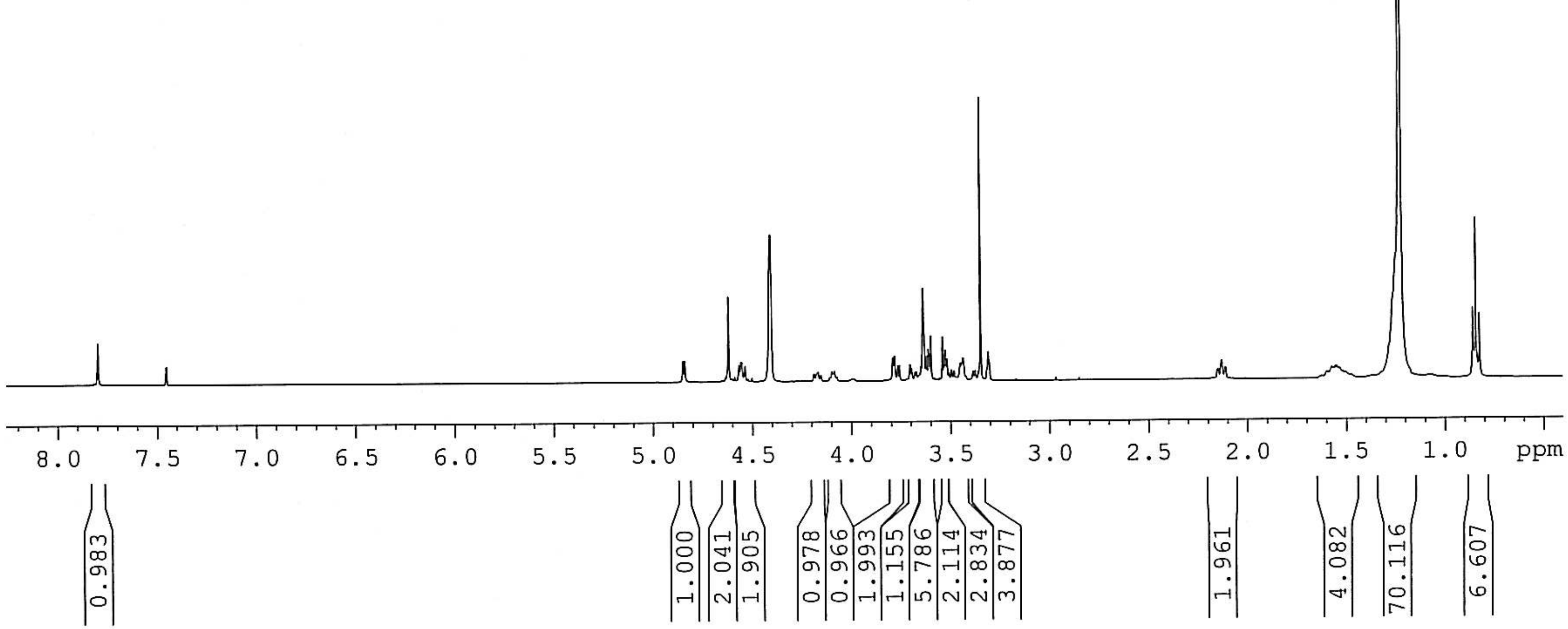

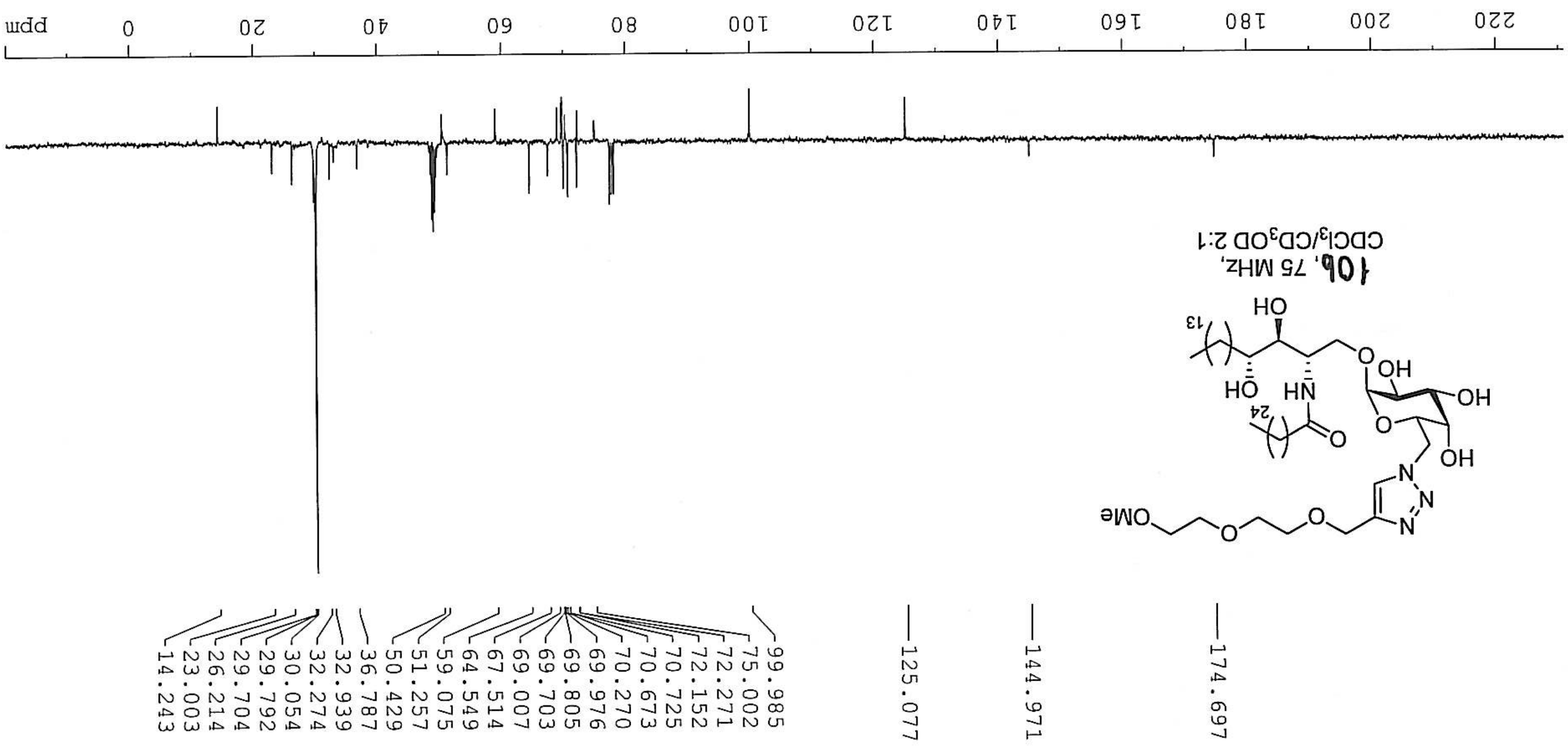

10b, 75 MHz, $CDCl_3/CD_3OD$ 2:1

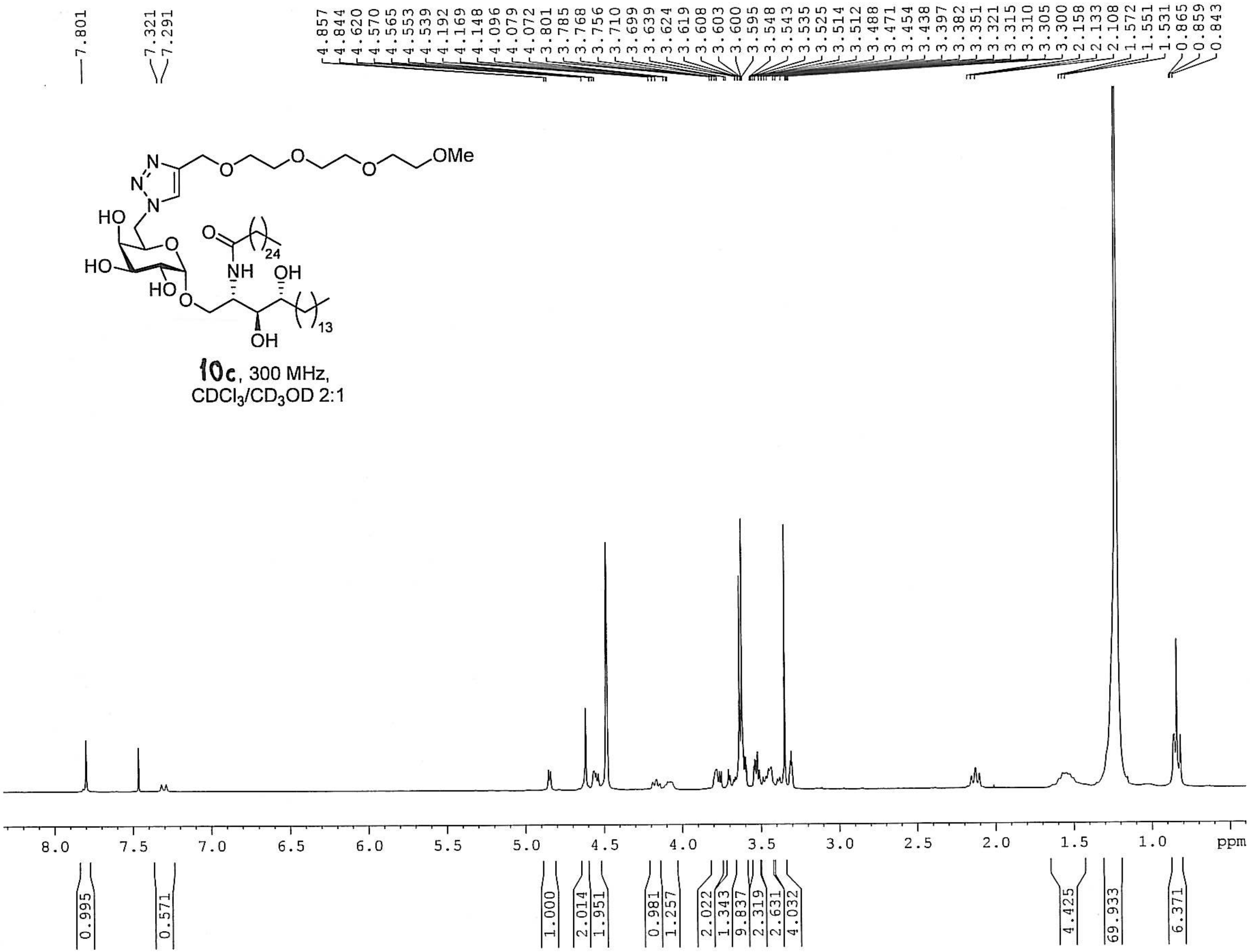

10c, 300 MHz, $CDCl_3/CD_3OD$ 2:1

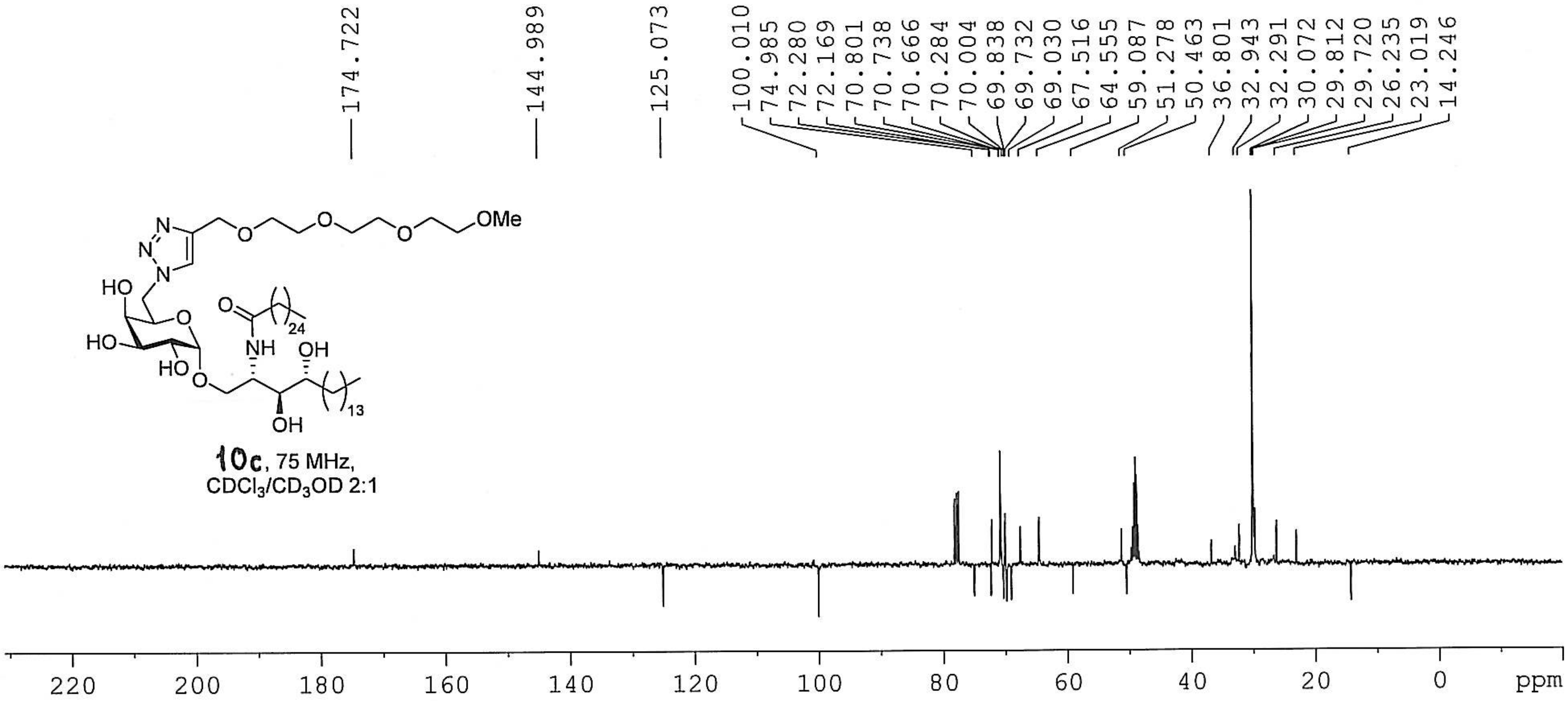

10c, 75 MHz, $CDCl_3/CD_3OD$ 2:1

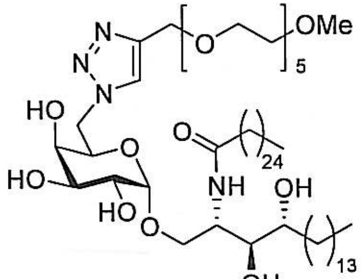

10d, 300 MHz, $CDCl_3/CD_3OD$ 2:1

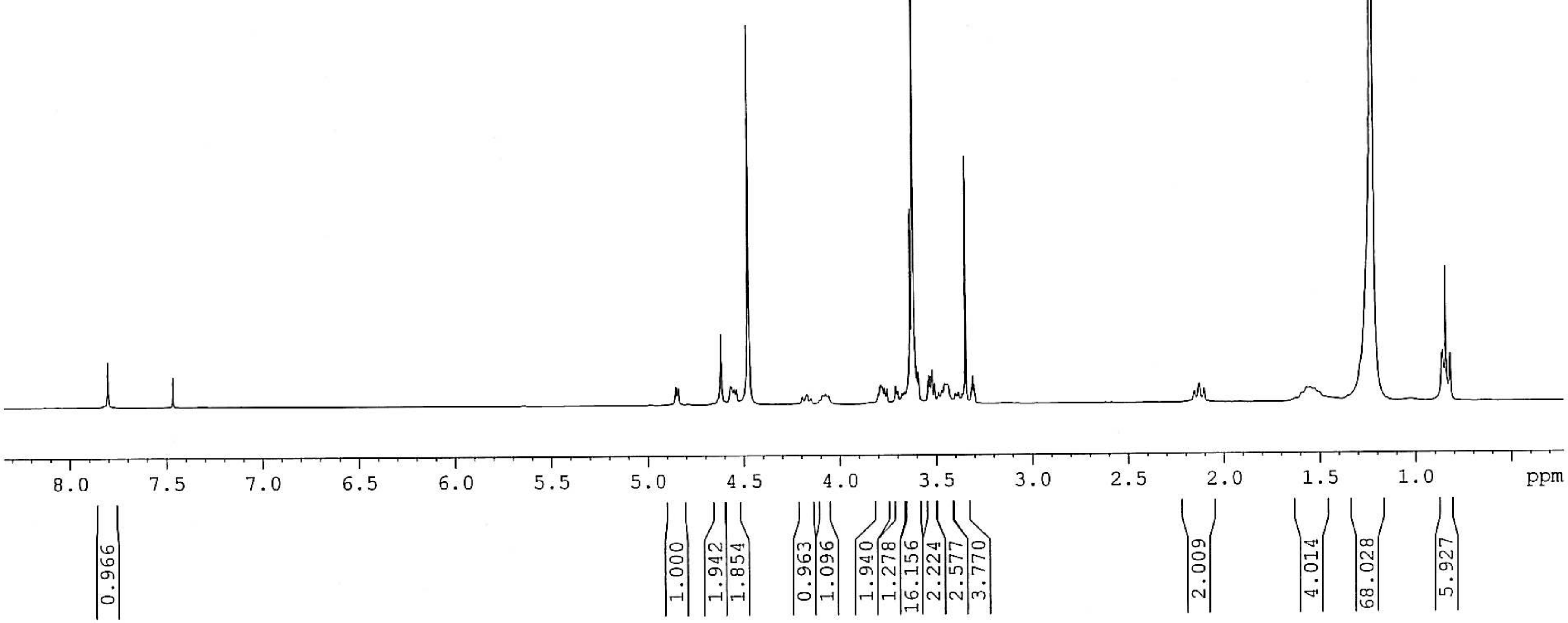

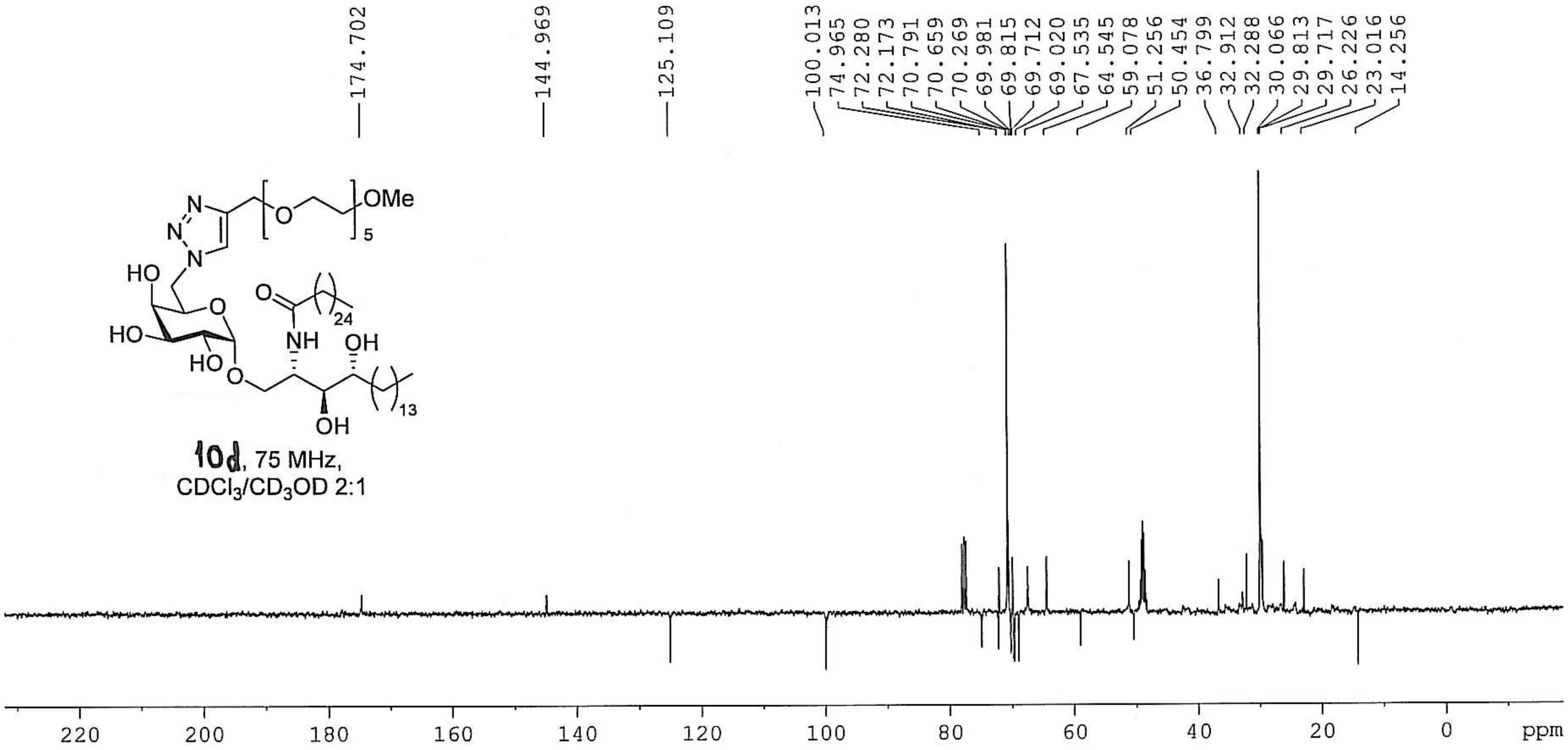

10d, 75 MHz, $CDCl_3/CD_3OD$ 2:1

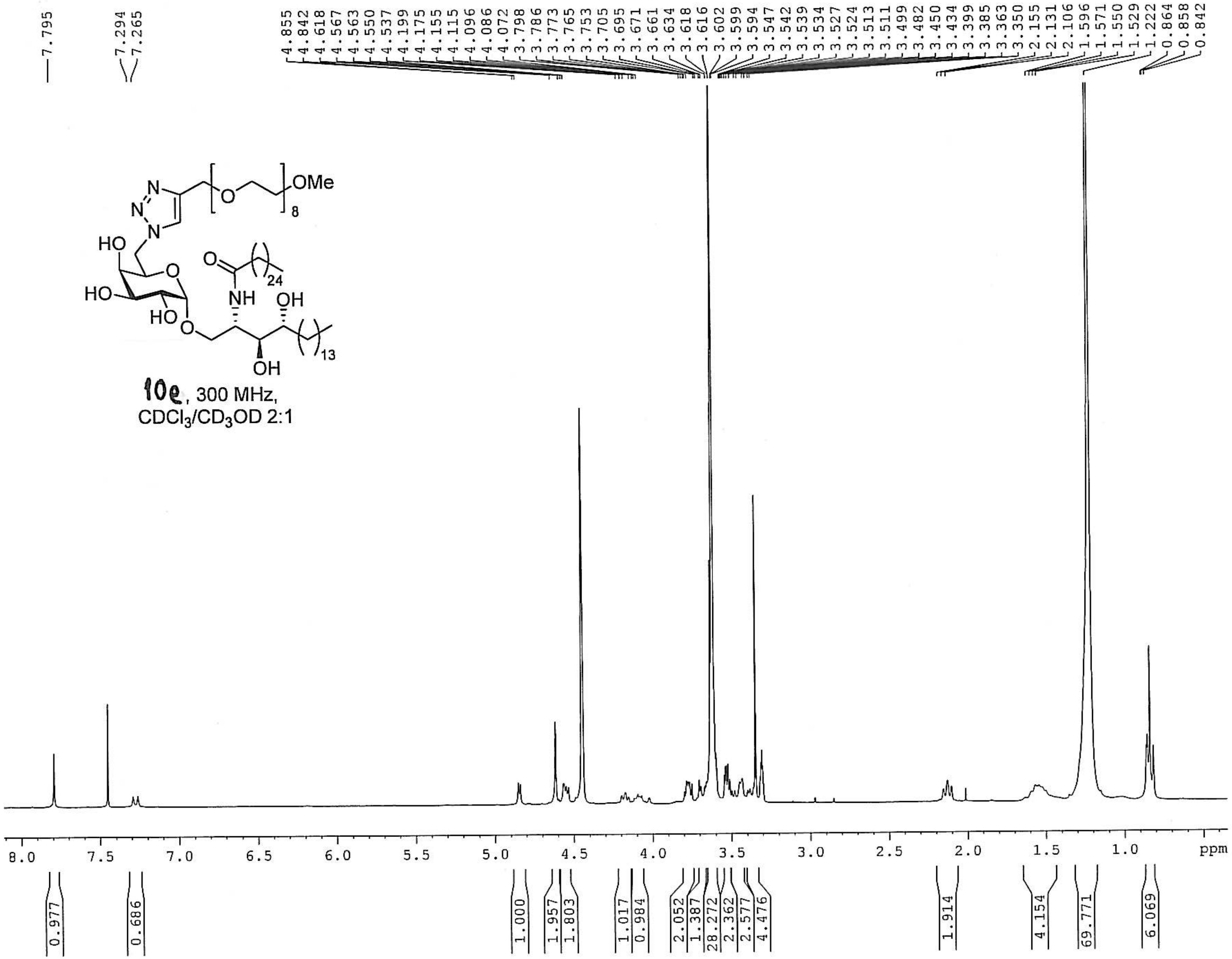

10e, 300 MHz, $CDCl_3/CD_3OD$ 2:1

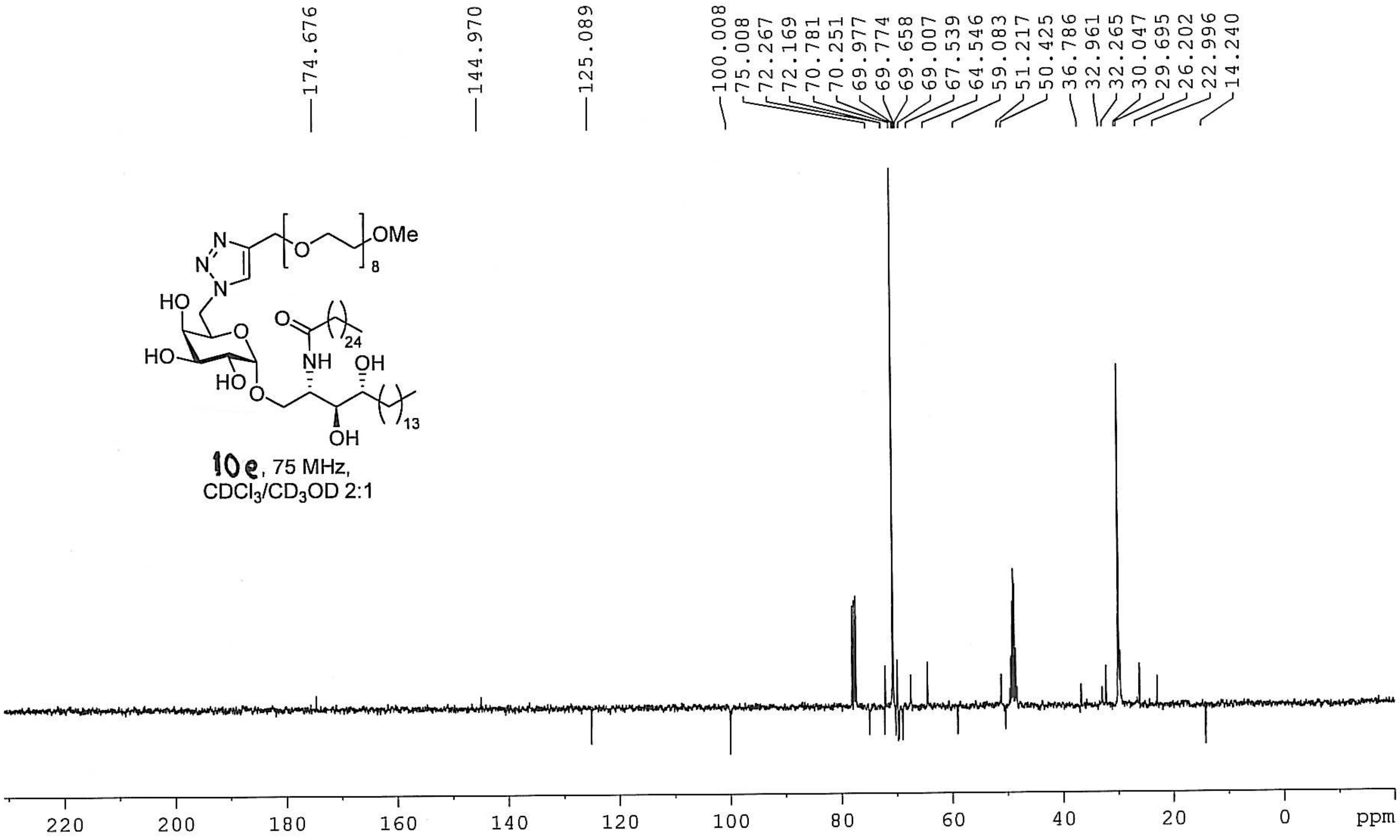

10e, 75 MHz, $CDCl_3/CD_3OD$ 2:1
174.676
144.970
125.089
100.008
75.008
72.267
72.169
70.781
70.251
69.977
69.774
69.658
69.007
67.539
64.546
59.083
51.217
50.425
36.786
32.961
32.265
30.047
29.695
26.202
22.996
14.240
220
200
180
160
140
120
100
80
60
40
20
0
ppm

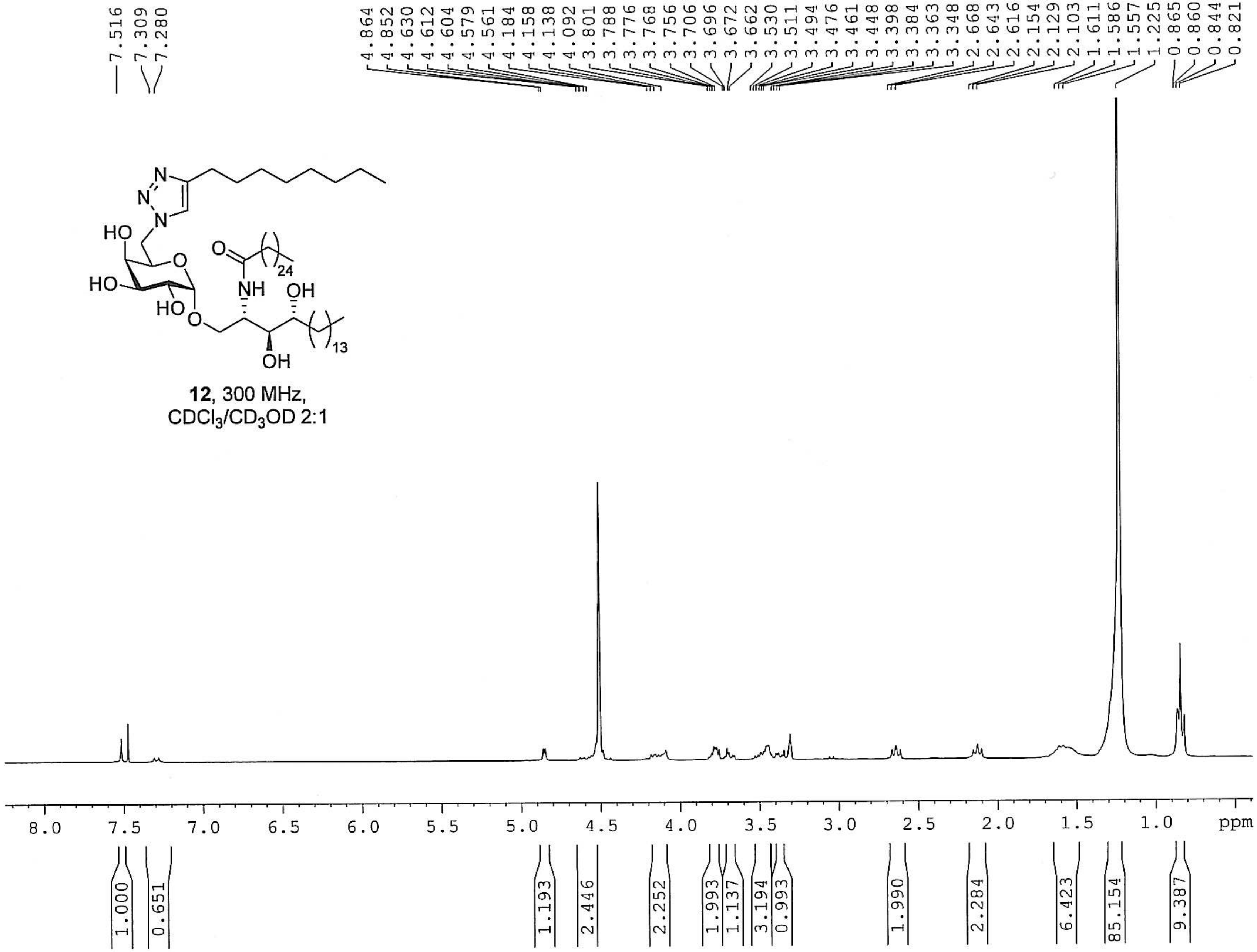

12, 300 MHz, $CDCl_3/CD_3OD$ 2:1

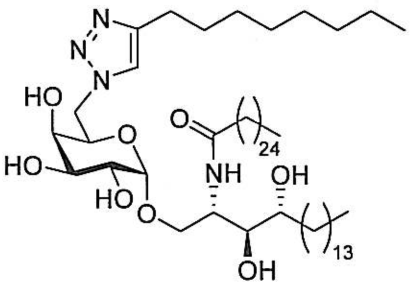

12, 75 MHz, $CDCl_3/CD_3OD$ 2:1

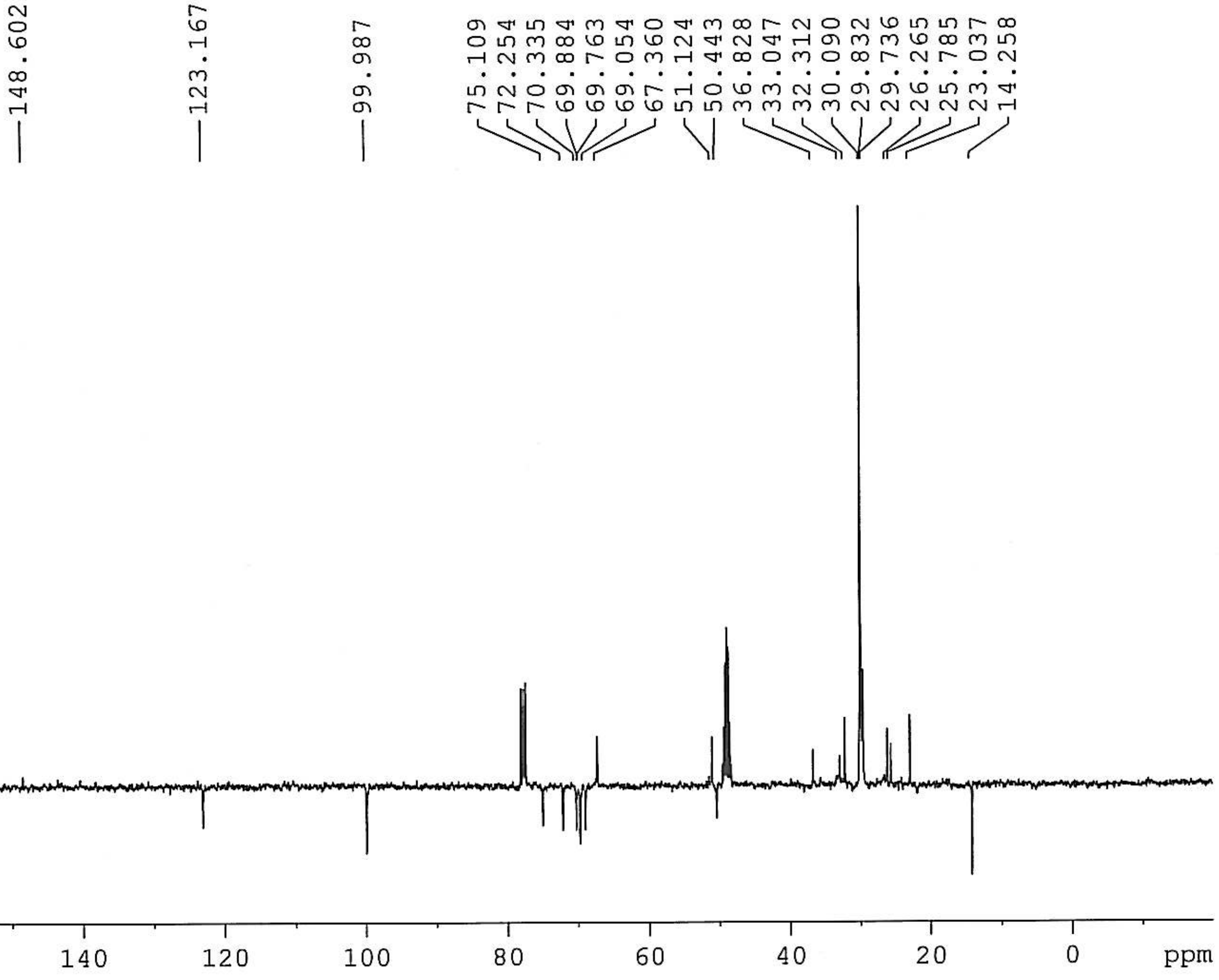

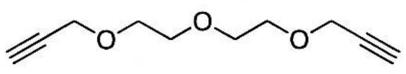

16b, 300 MHz, $CDCl_3$

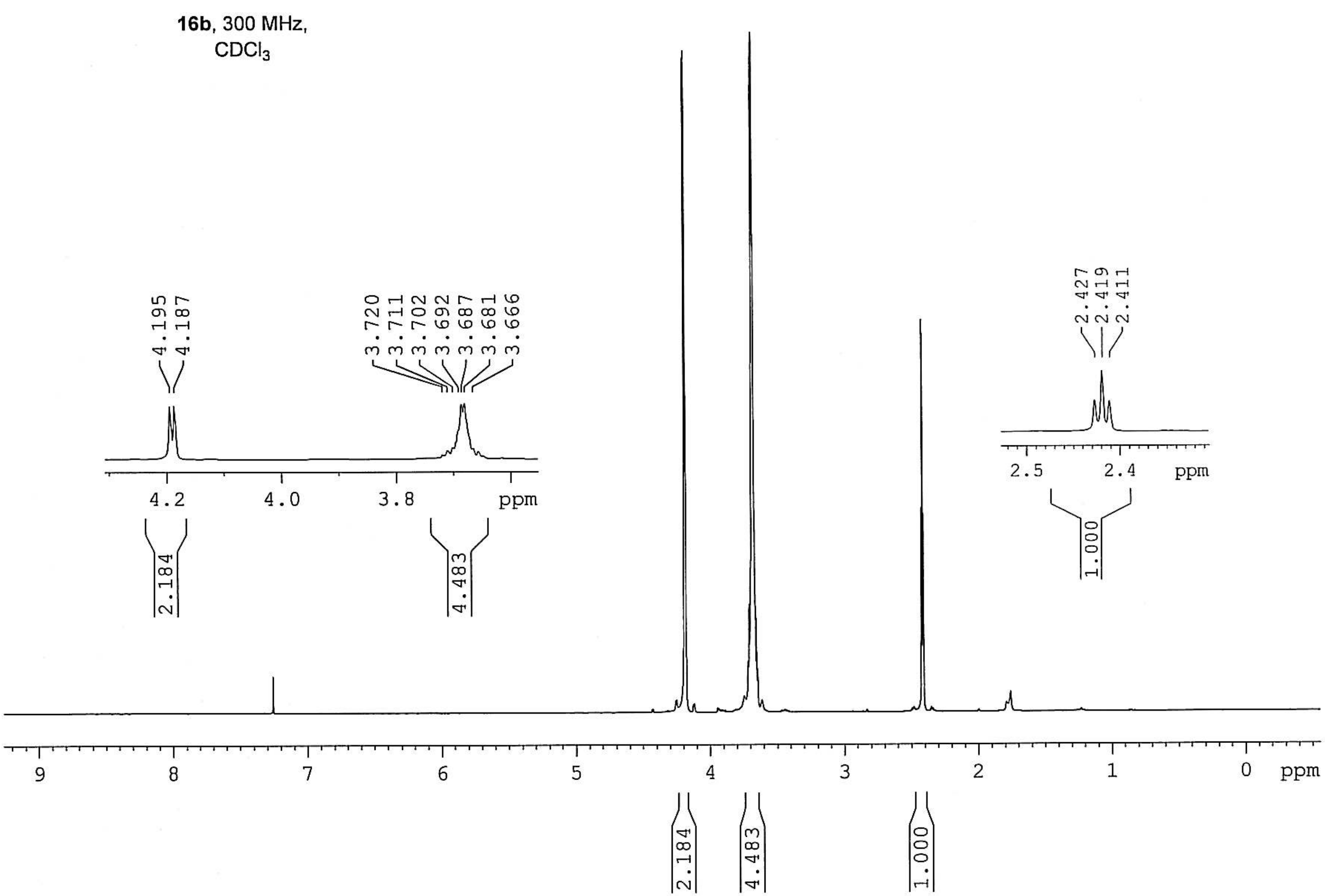

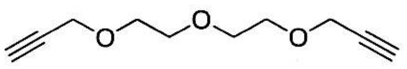

16b, 75 MHz, $CDCl_3$

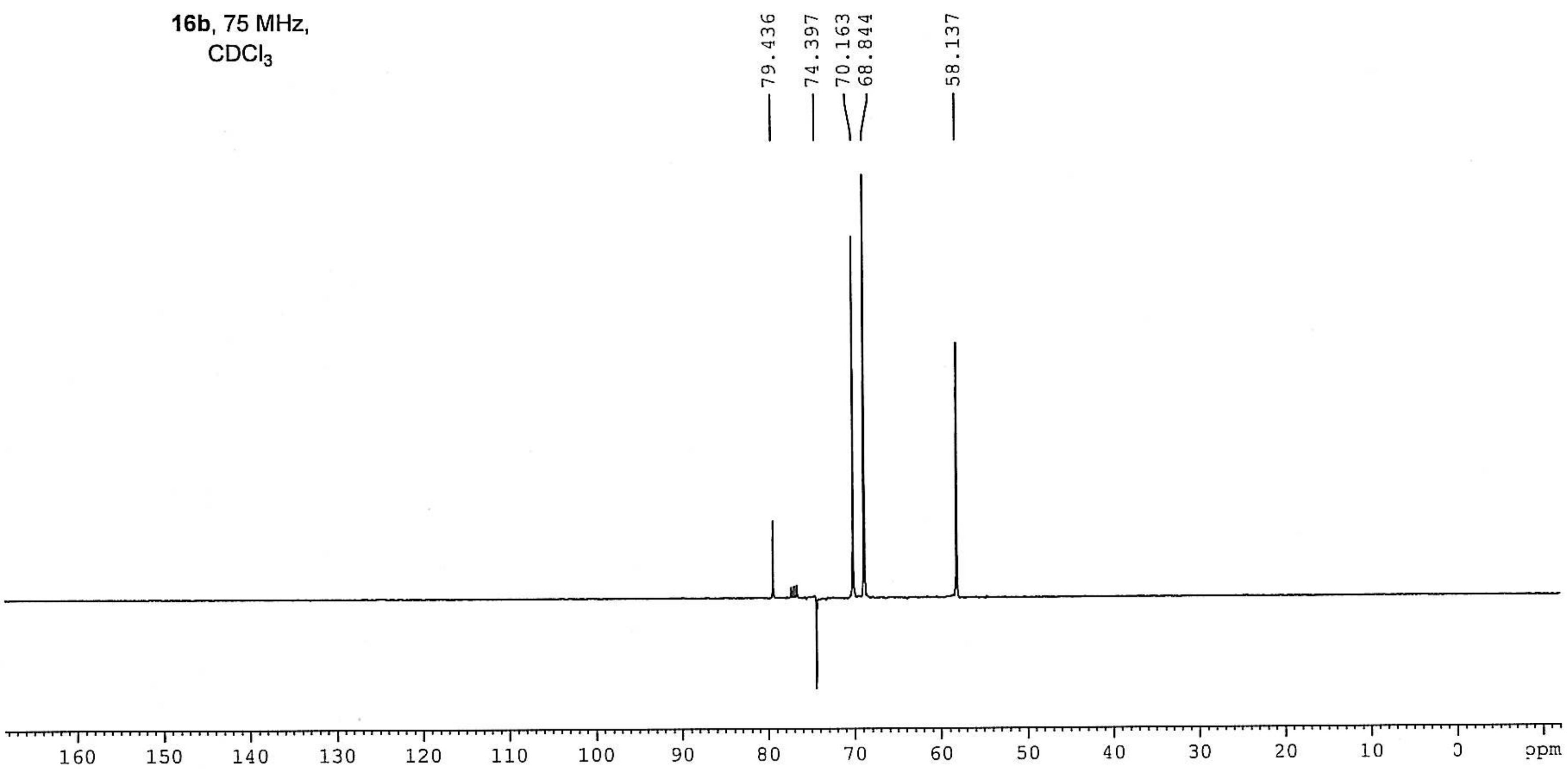

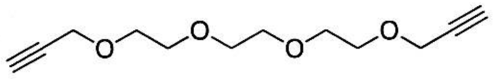

16c, 300 MHz, $CDCl_3$

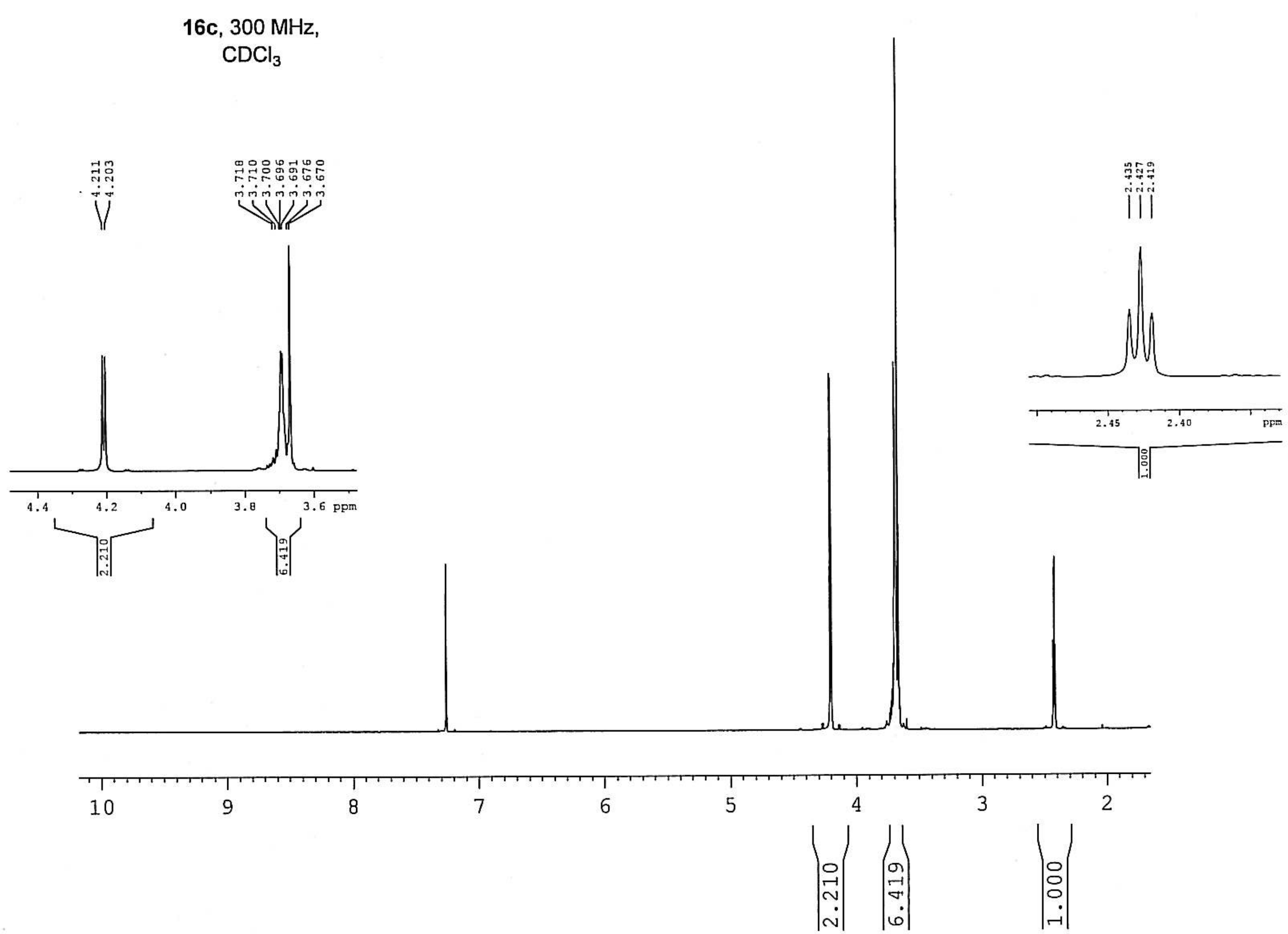

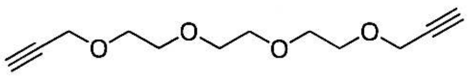

16c, 75 MHz, $CDCl_3$

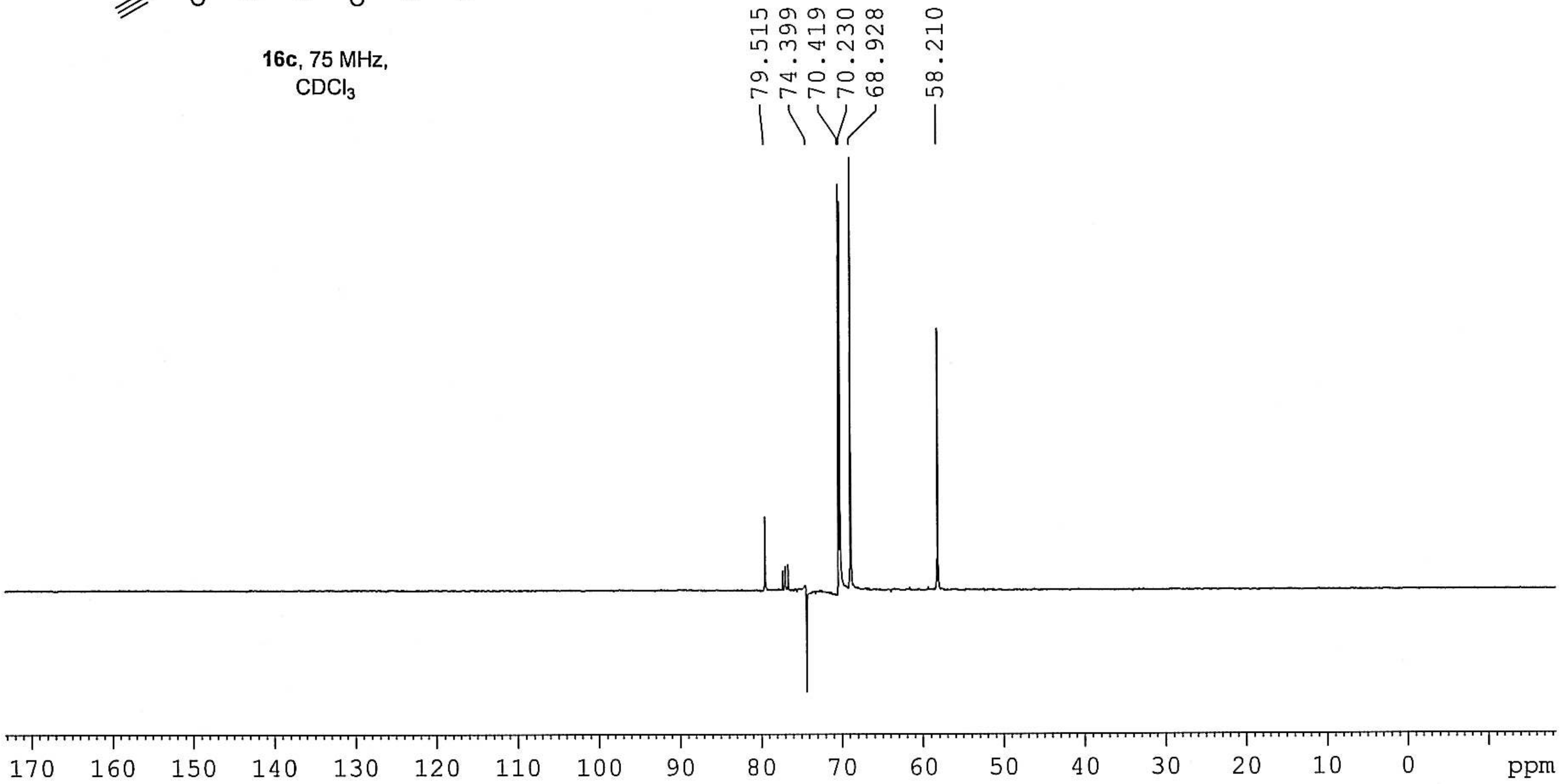

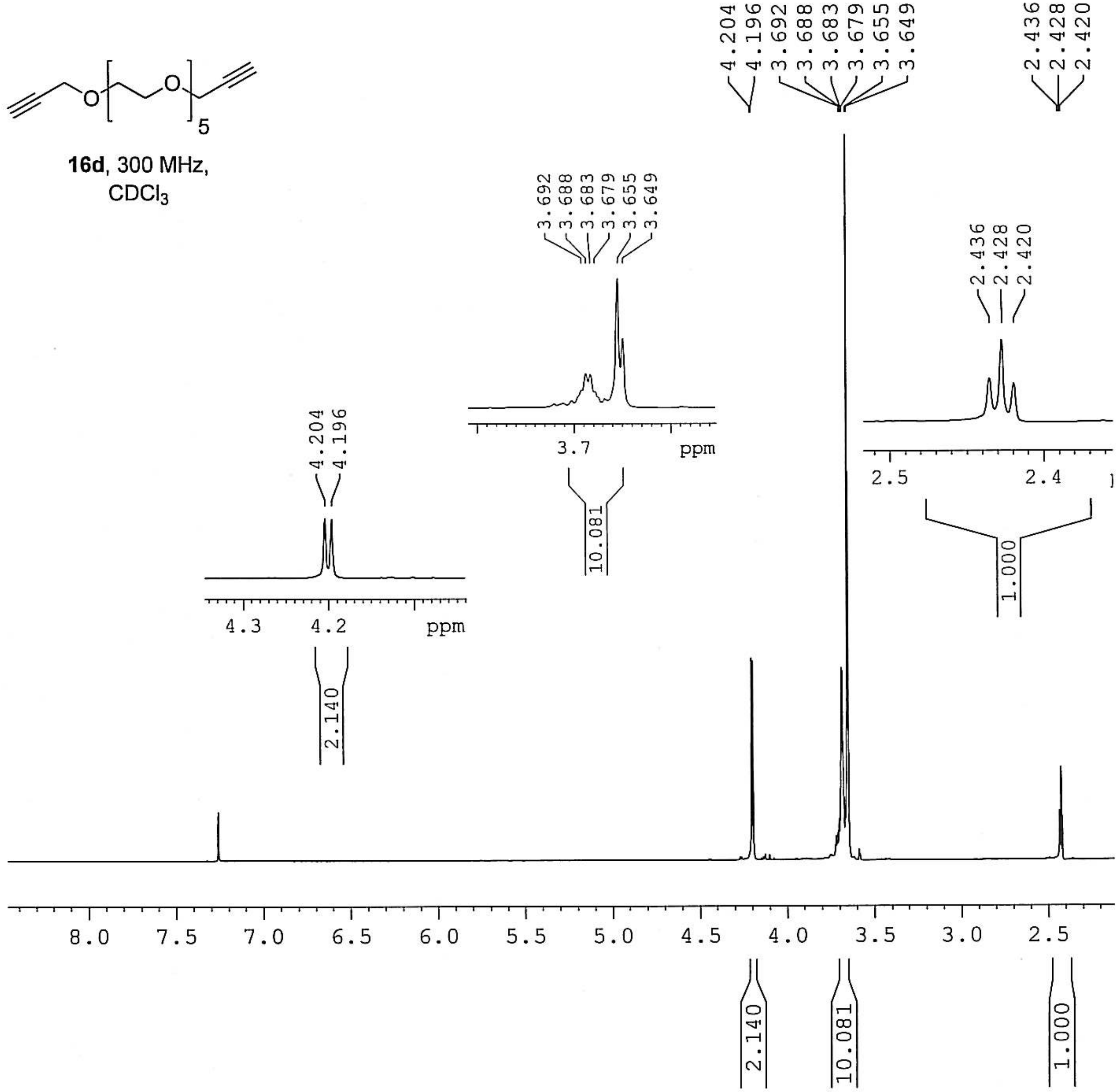

16d, 300 MHz, $CDCl_3$

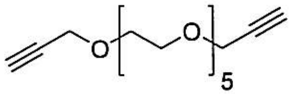

16d, 75 MHz,
$CDCl_3$

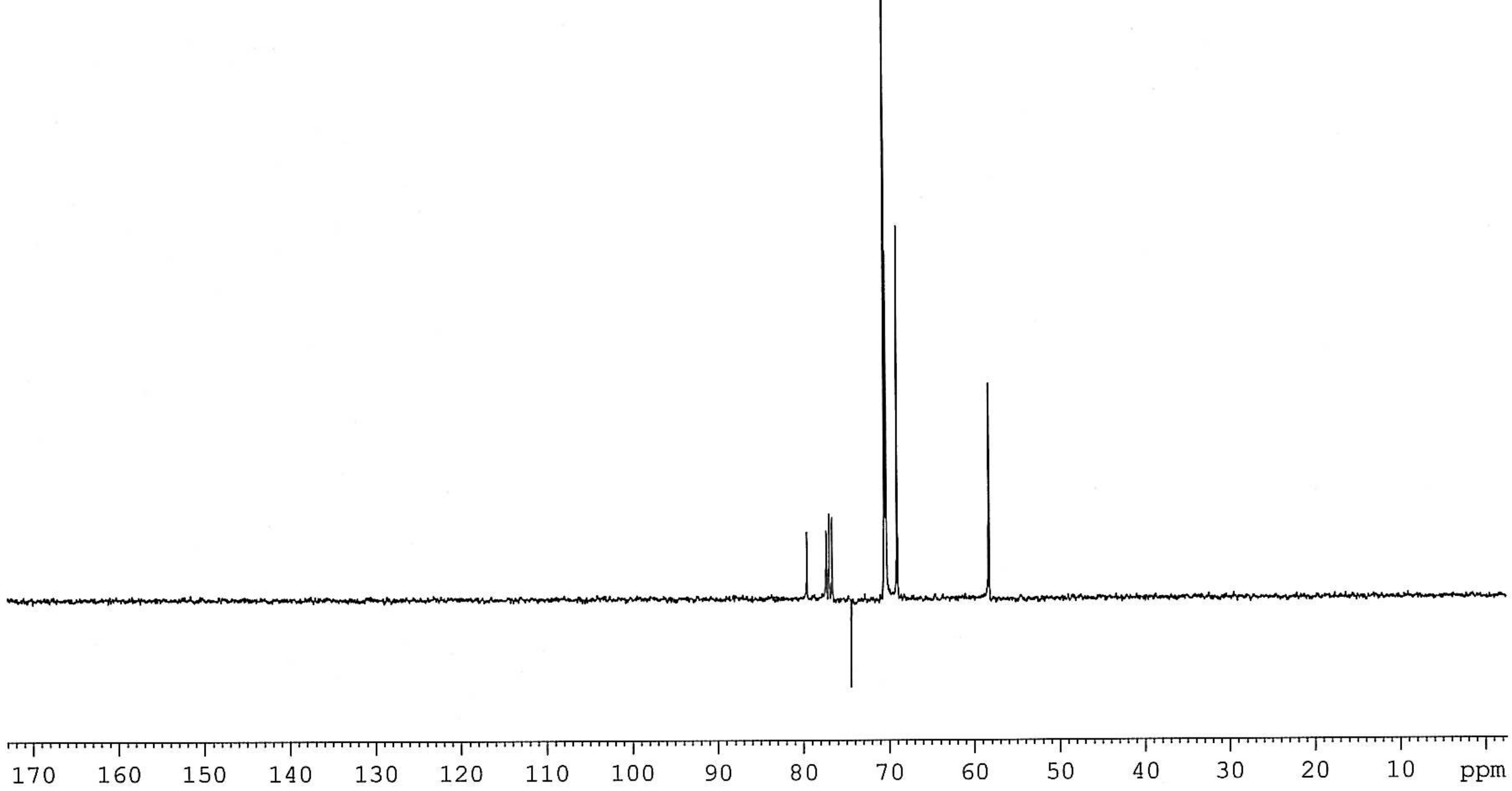

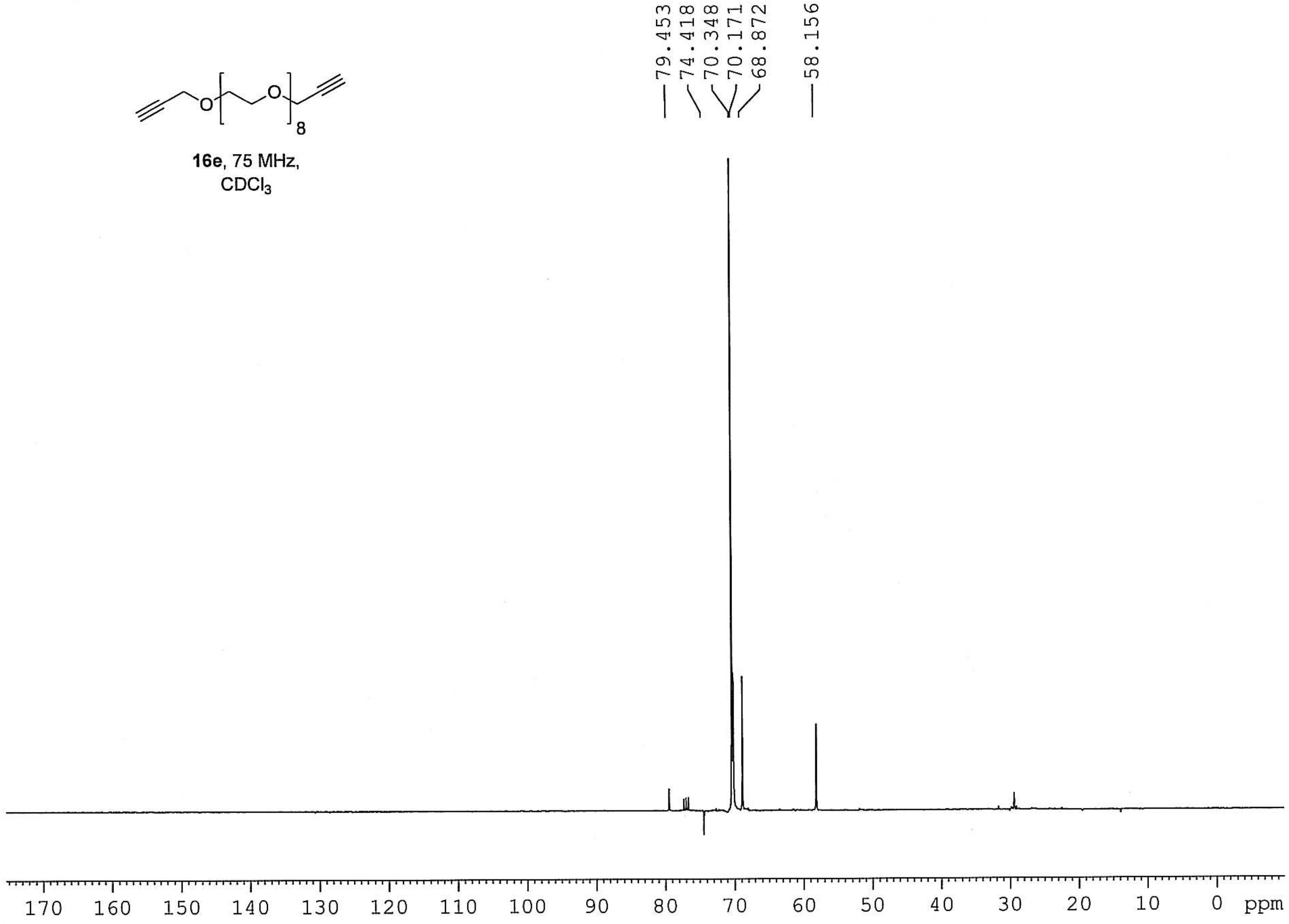

16e, 75 MHz, $CDCl_3$

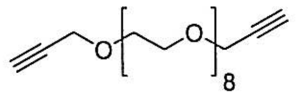

16e, 300 MHz, $CDCl_3$

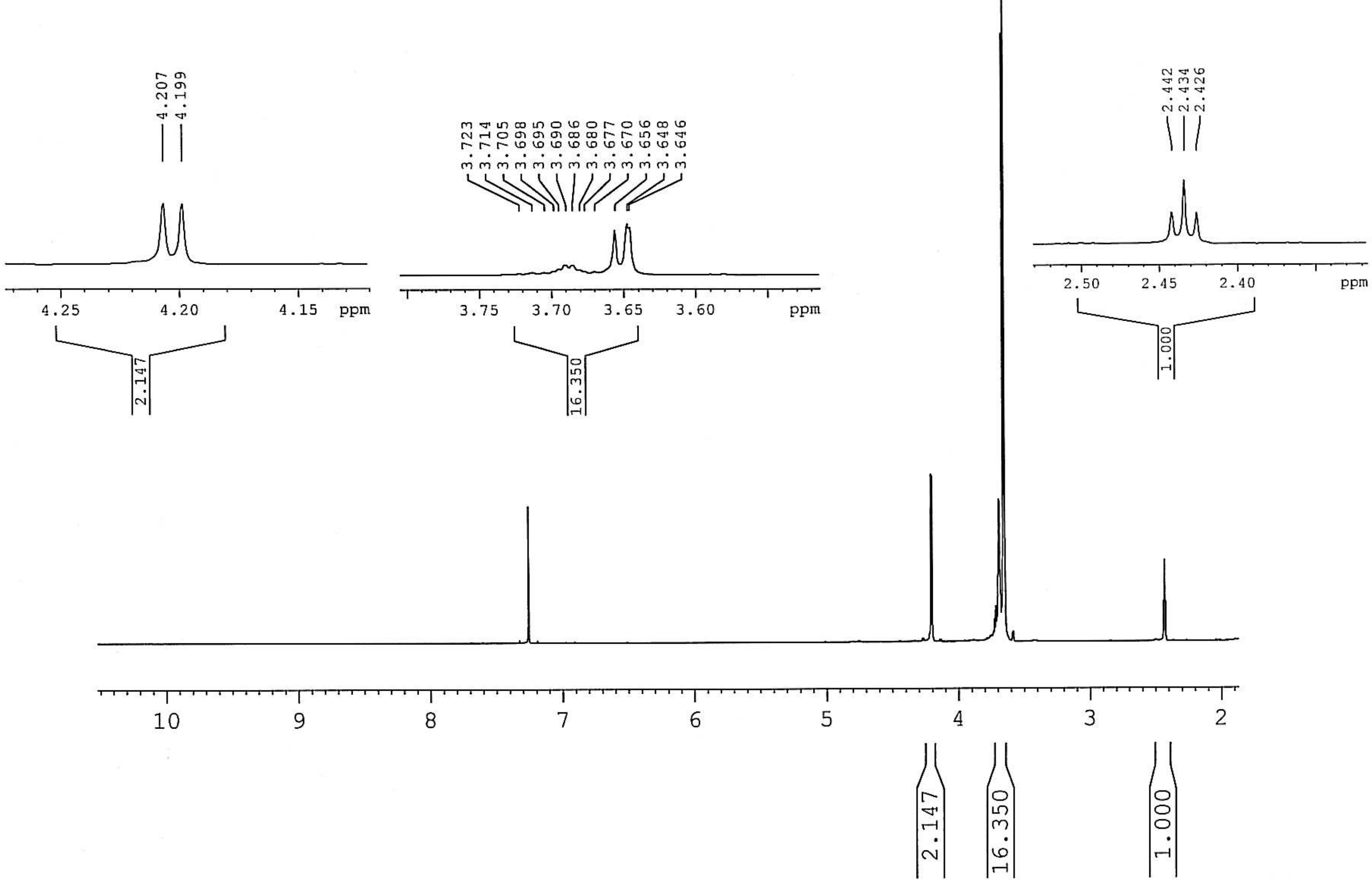

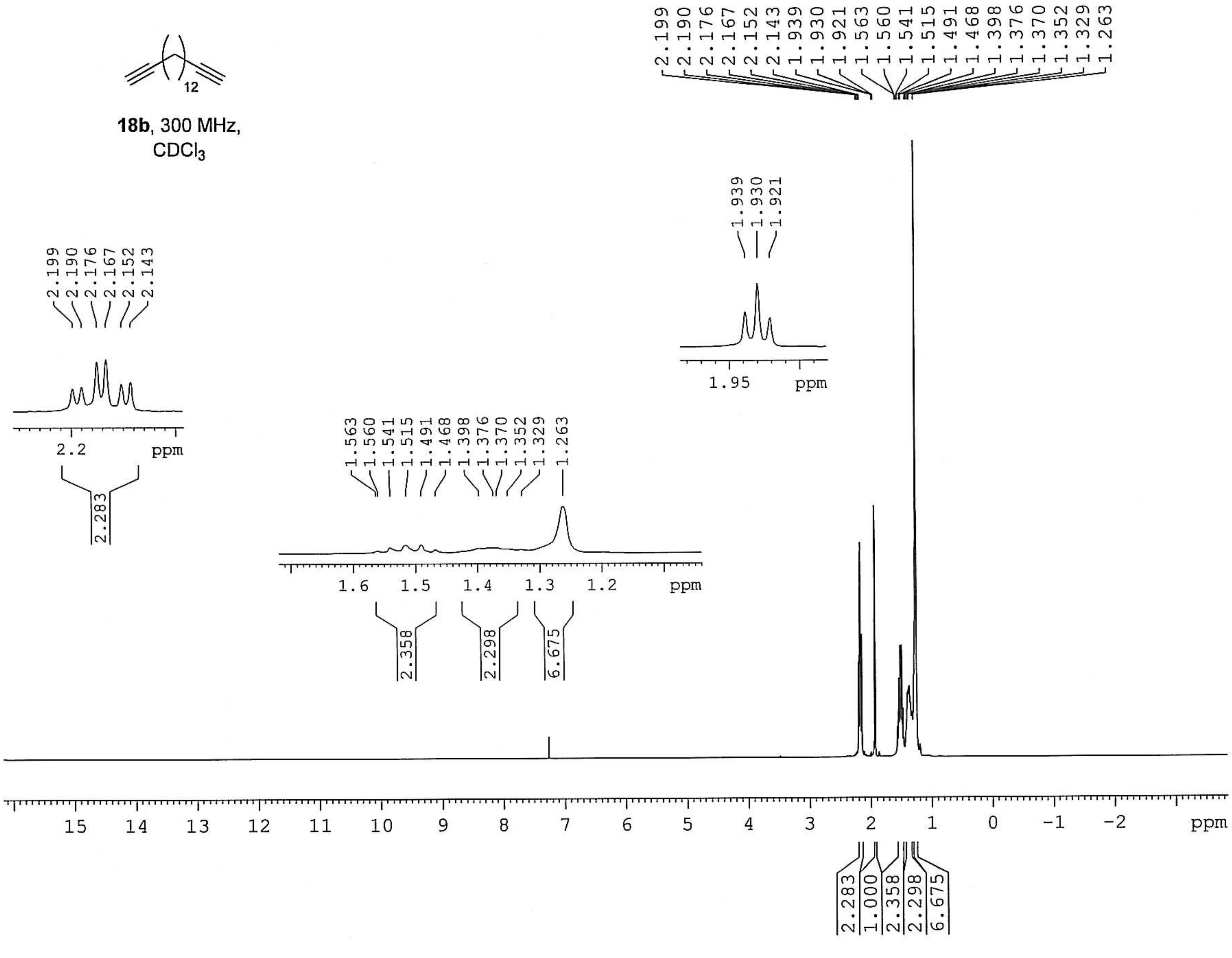

12
18b, 300 MHz,
CDCl3
2.199
2.190
2.176
2.167
2.152
2.143
1.939
1.930
1.921
1.563
1.560
1.541
1.515
1.491
1.468
1.398
1.376
1.370
1.352
1.329
1.263
2.2
ppm
2.283
1.95
ppm
1.6
1.5
1.4
1.3
1.2
ppm
2.358
2.298
6.675
15
14
13
12
11
10
9
8
7
6
5
4
3
2
1
0
-1
-2
ppm
2.283
1.000
2.358
2.298
6.675

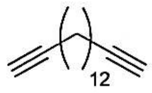

18b, 75 MHz, $CDCl_3$

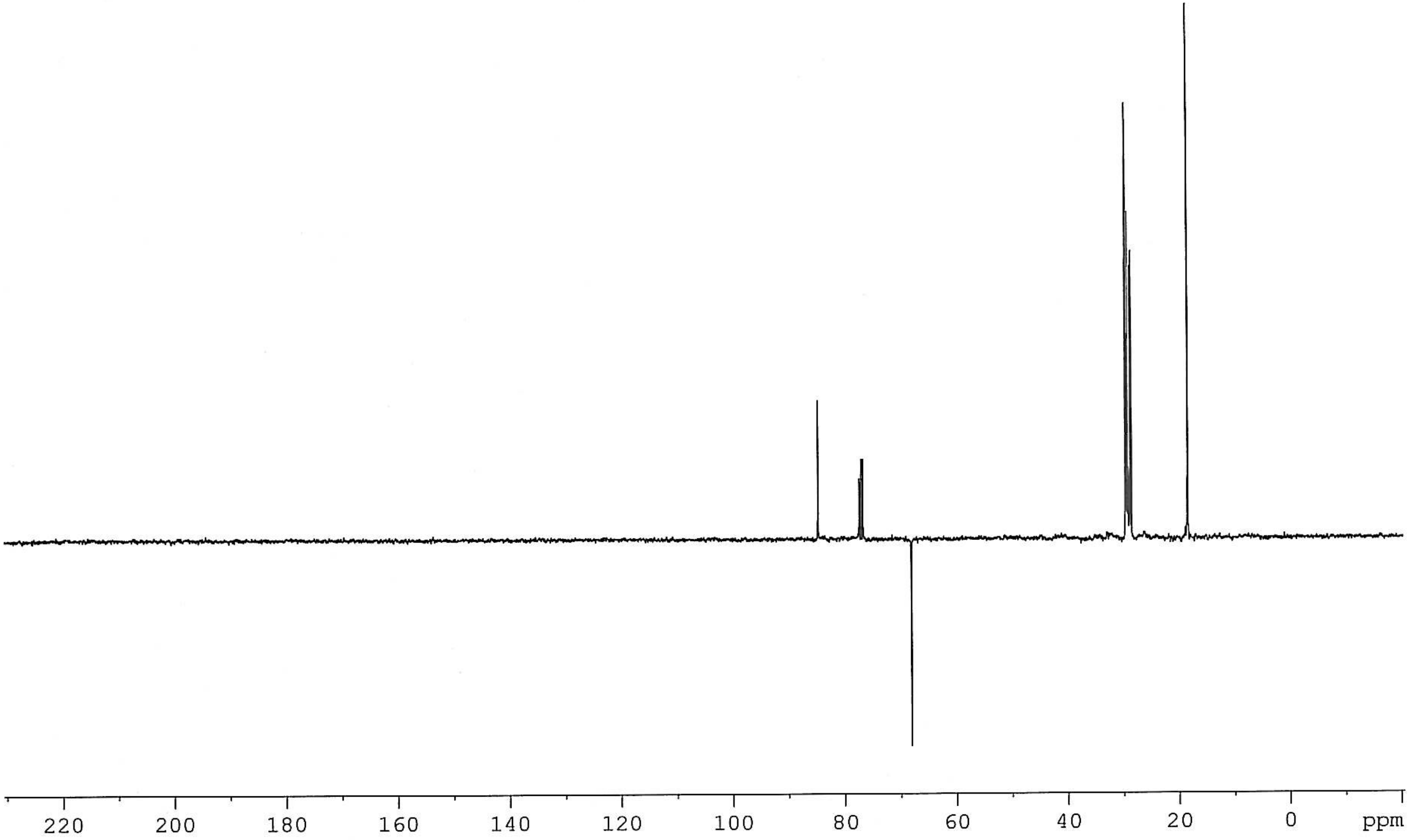

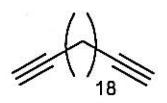

18c, 300 MHz,
$CDCl_3$

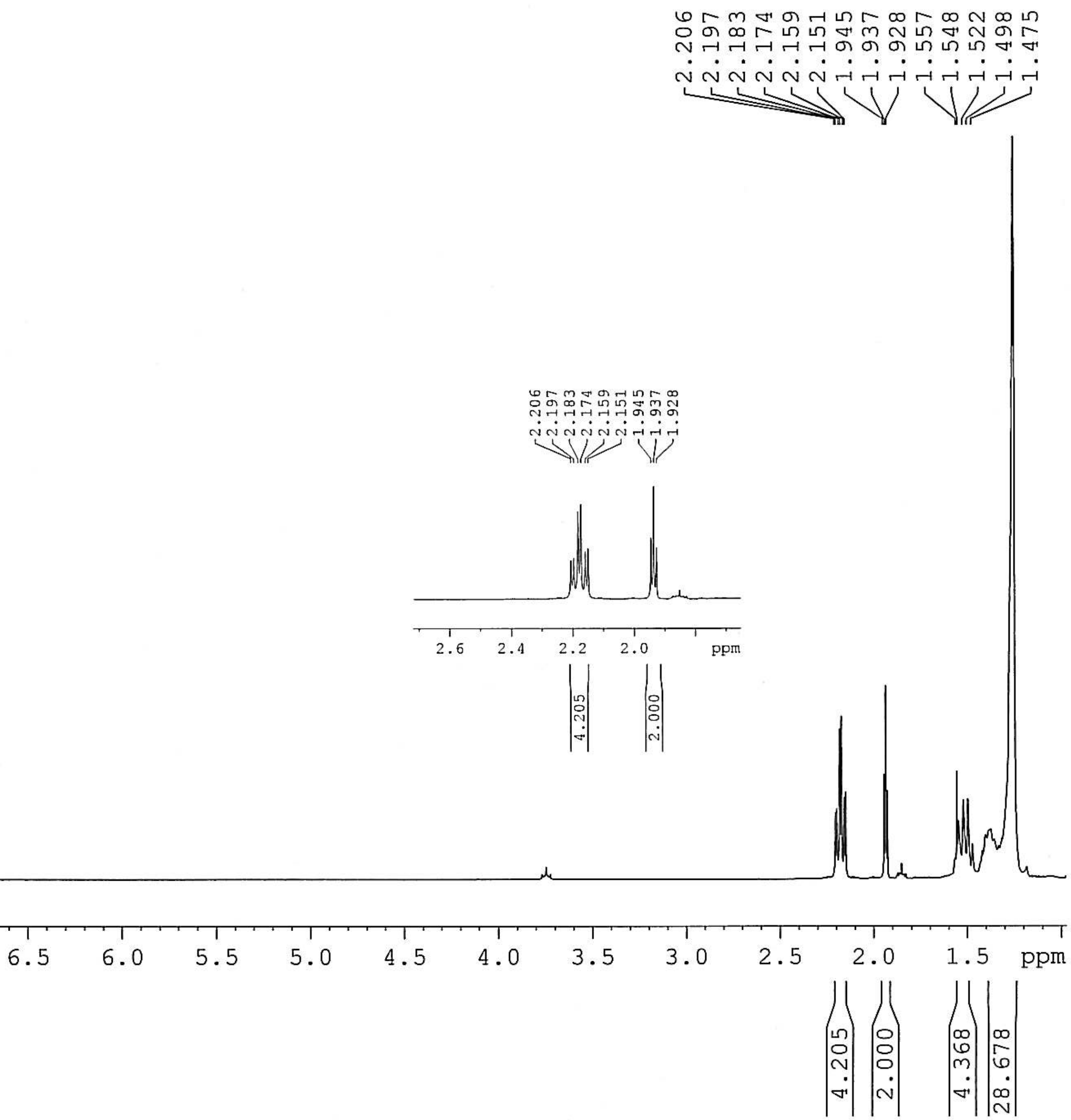

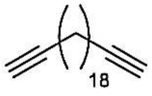

18c, 75 MHz, $CDCl_3$

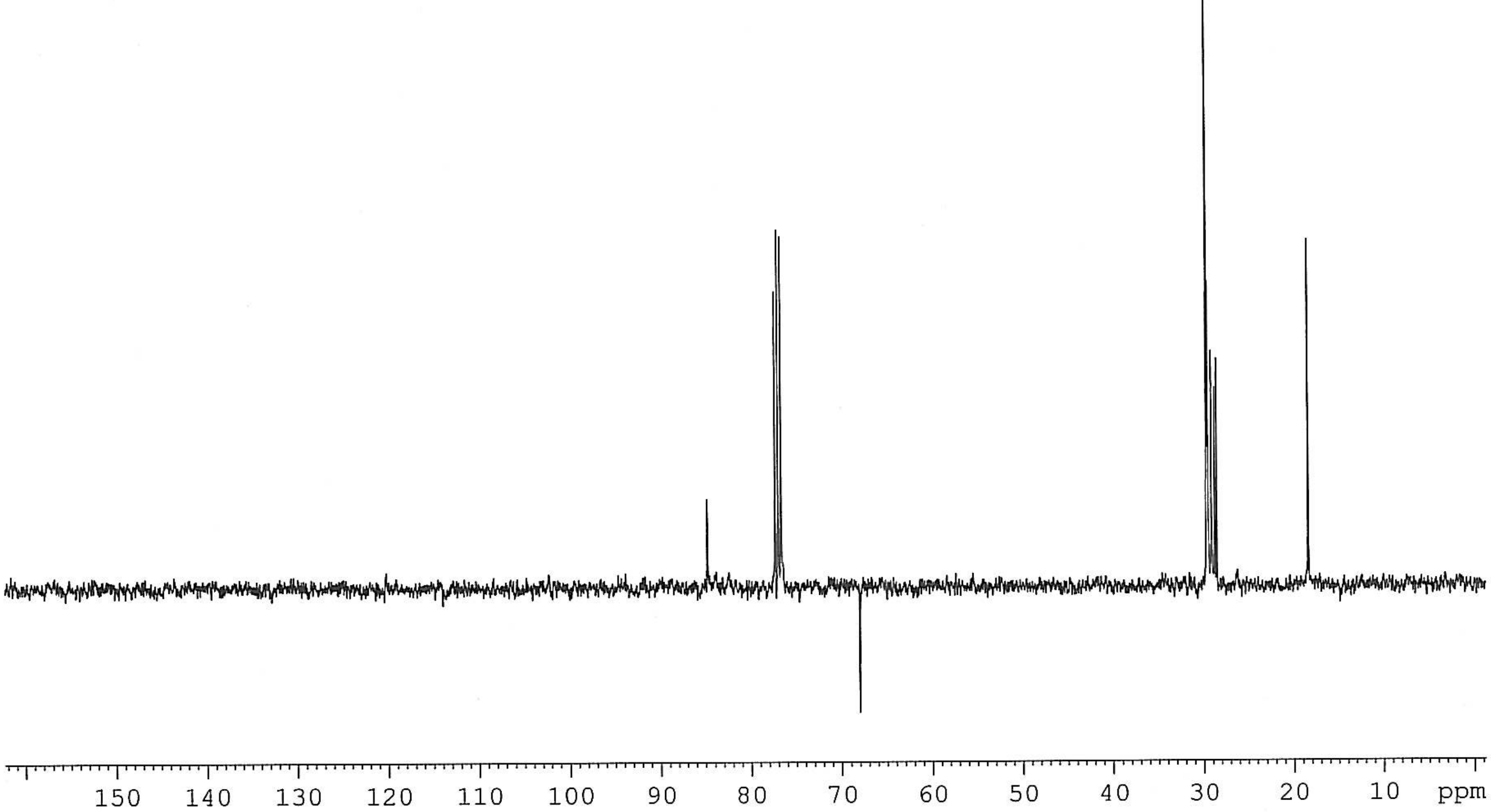

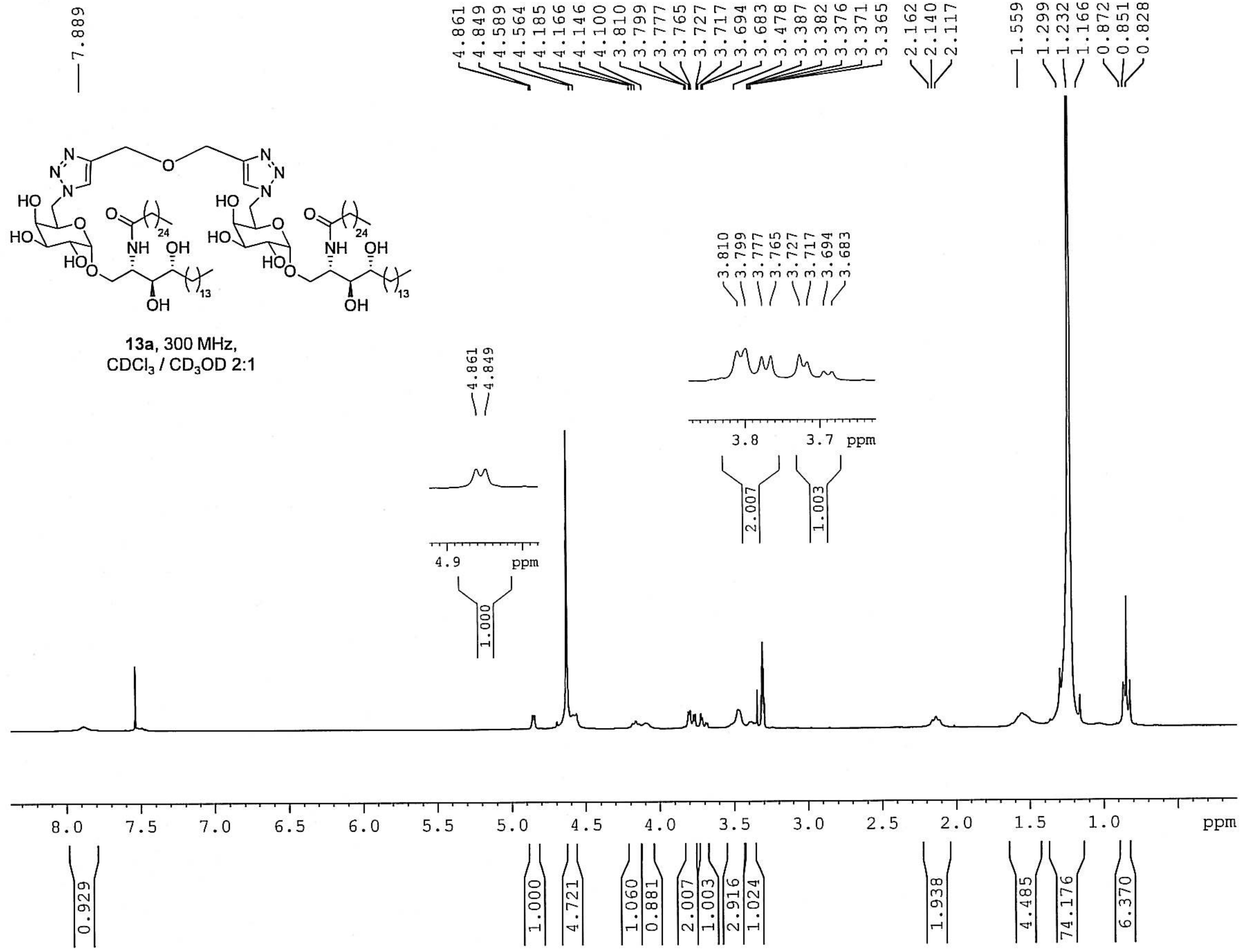

13a, 300 MHz,
$CDCl_3$ / CD_3OD 2:1

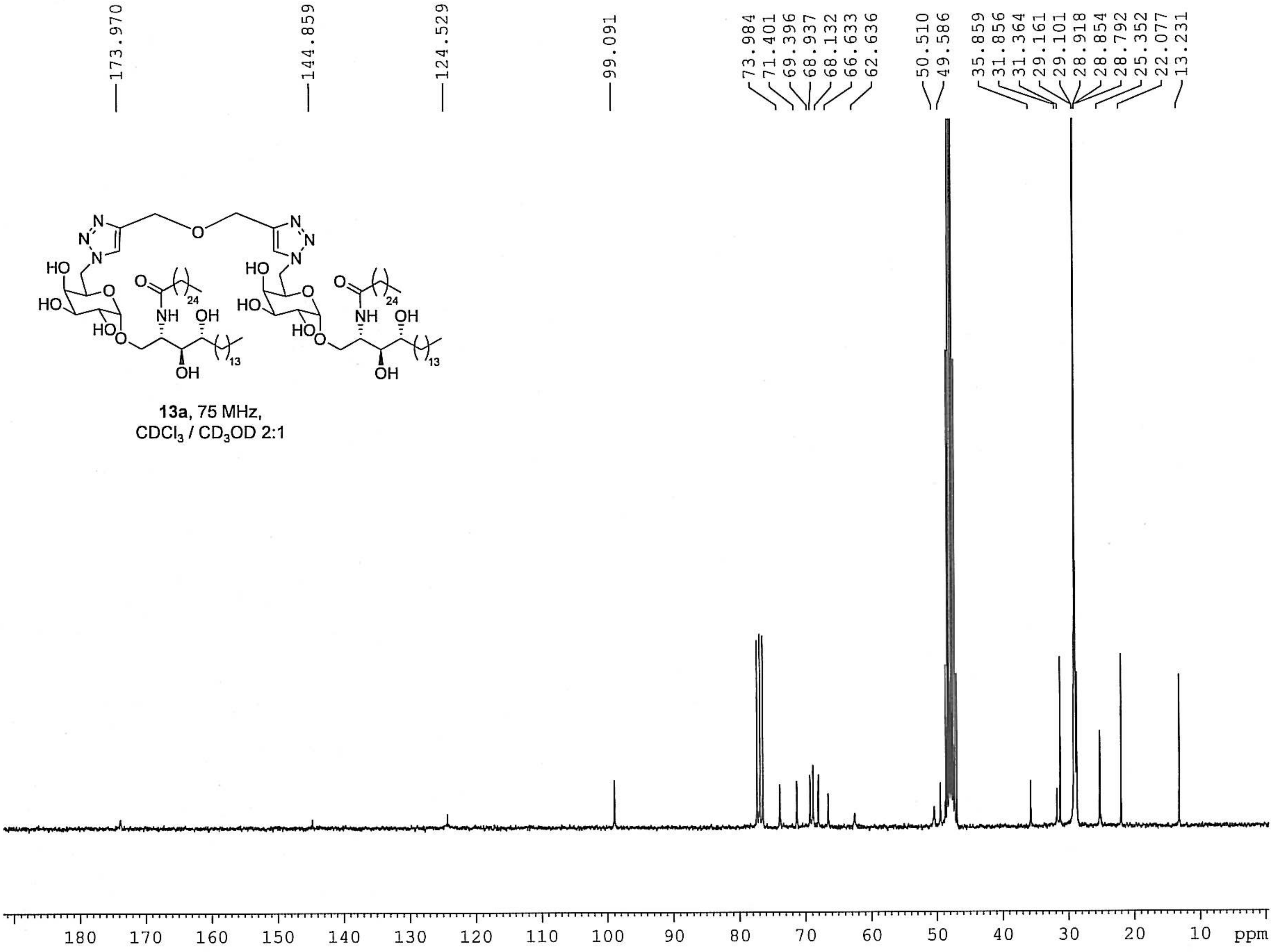

13a, 75 MHz,
$CDCl_3$ / CD_3OD 2:1

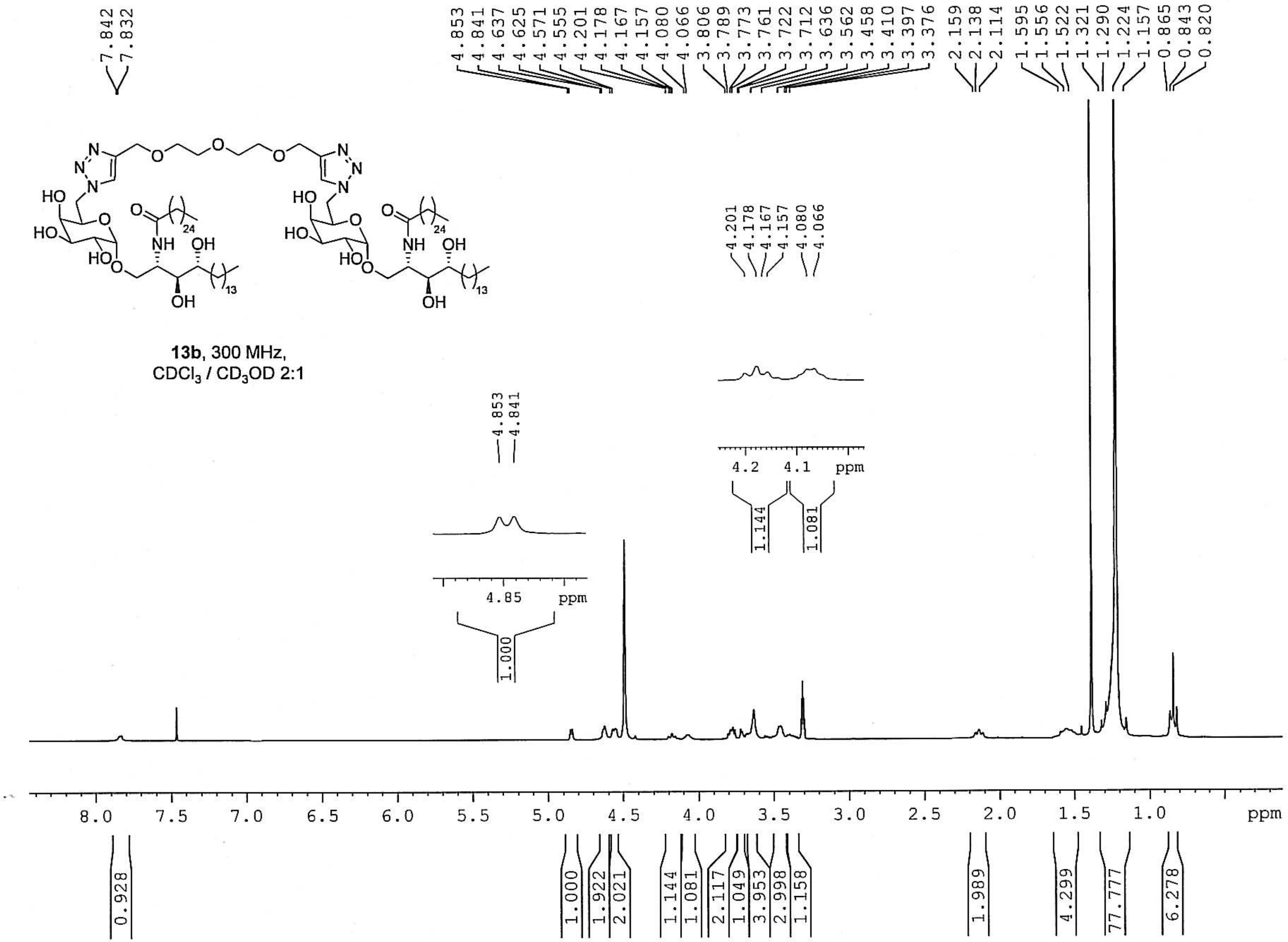
13b, 300 MHz,
CDCl3 / CD3OD 2:1
7.842
7.832
4.853
4.841
4.637
4.625
4.571
4.555
4.201
4.178
4.167
4.157
4.080
4.066
3.806
3.789
3.773
3.761
3.722
3.712
3.636
3.562
3.458
3.410
3.397
3.376
2.159
2.138
2.114
1.595
1.556
1.522
1.321
1.290
1.224
1.157
0.865
0.843
0.820
4.85
ppm
1.000
4.2
4.1
ppm
1.144
1.081
8.0
7.5
7.0
6.5
6.0
5.5
5.0
4.5
4.0
3.5
3.0
2.5
2.0
1.5
1.0
ppm
0.928
1.000
1.922
2.021
1.144
1.081
2.117
1.049
3.953
2.998
1.158
1.989
4.299
77.777
6.278

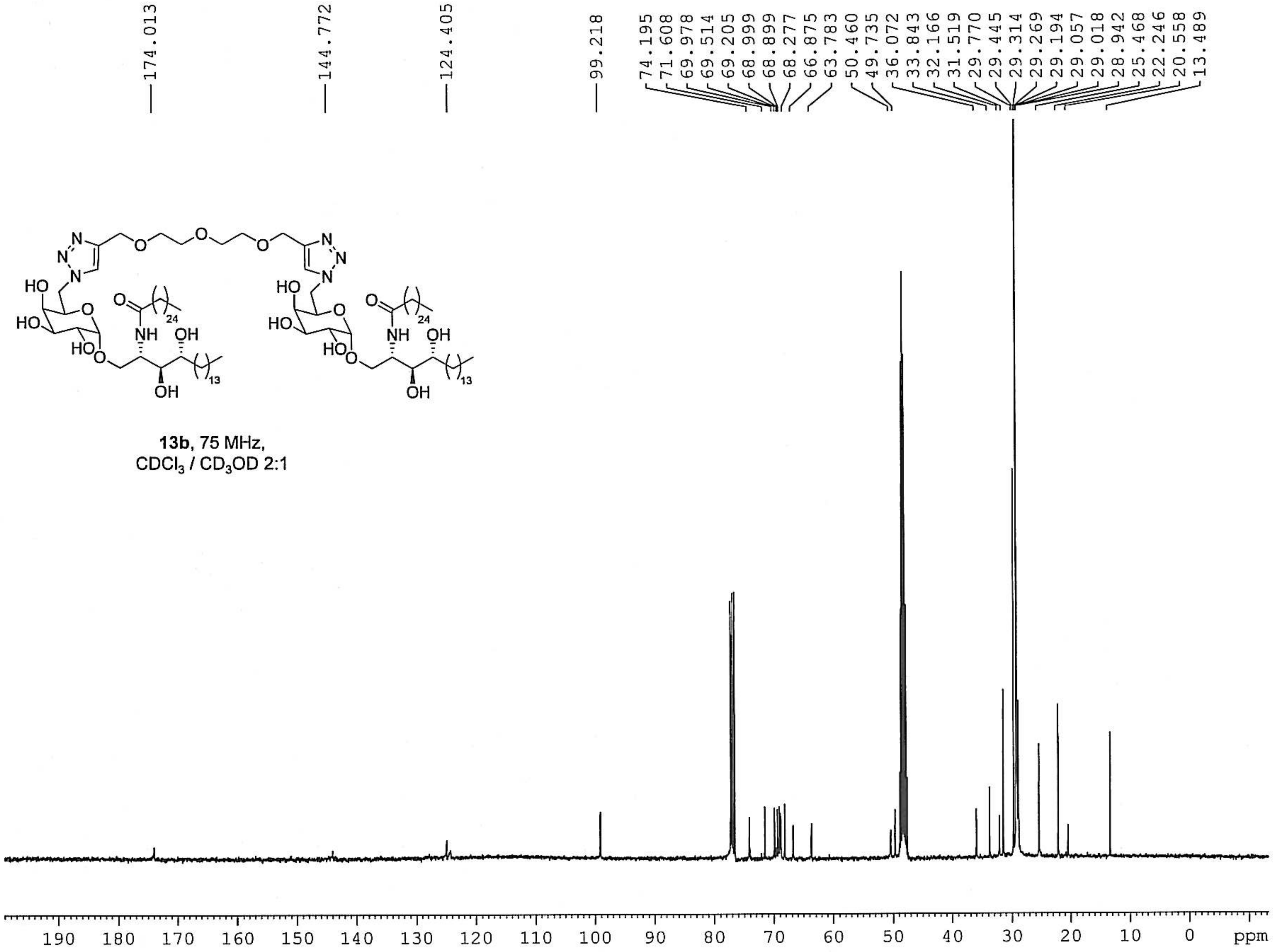

13b, 75 MHz,
$CDCl_3$ / CD_3OD 2:1

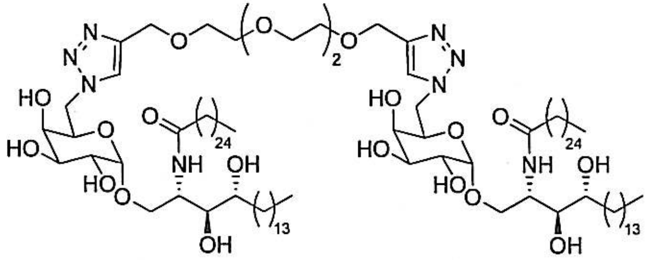

13c, 400 MHz, $CDCl_3$ / CD_3OD 2:1

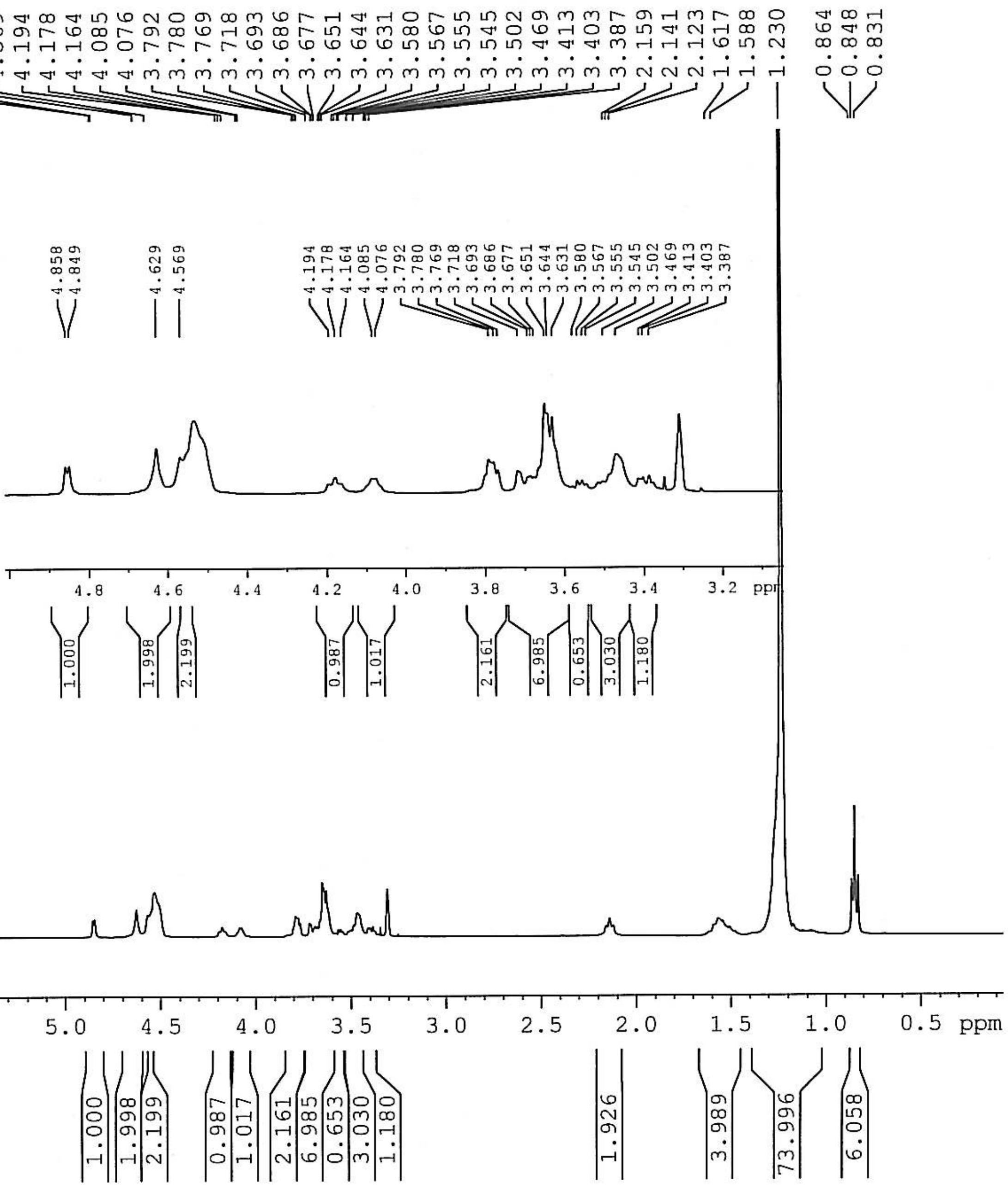

13c, 100 MHz,
$CDCl_3$ / CD_3OD 2:1

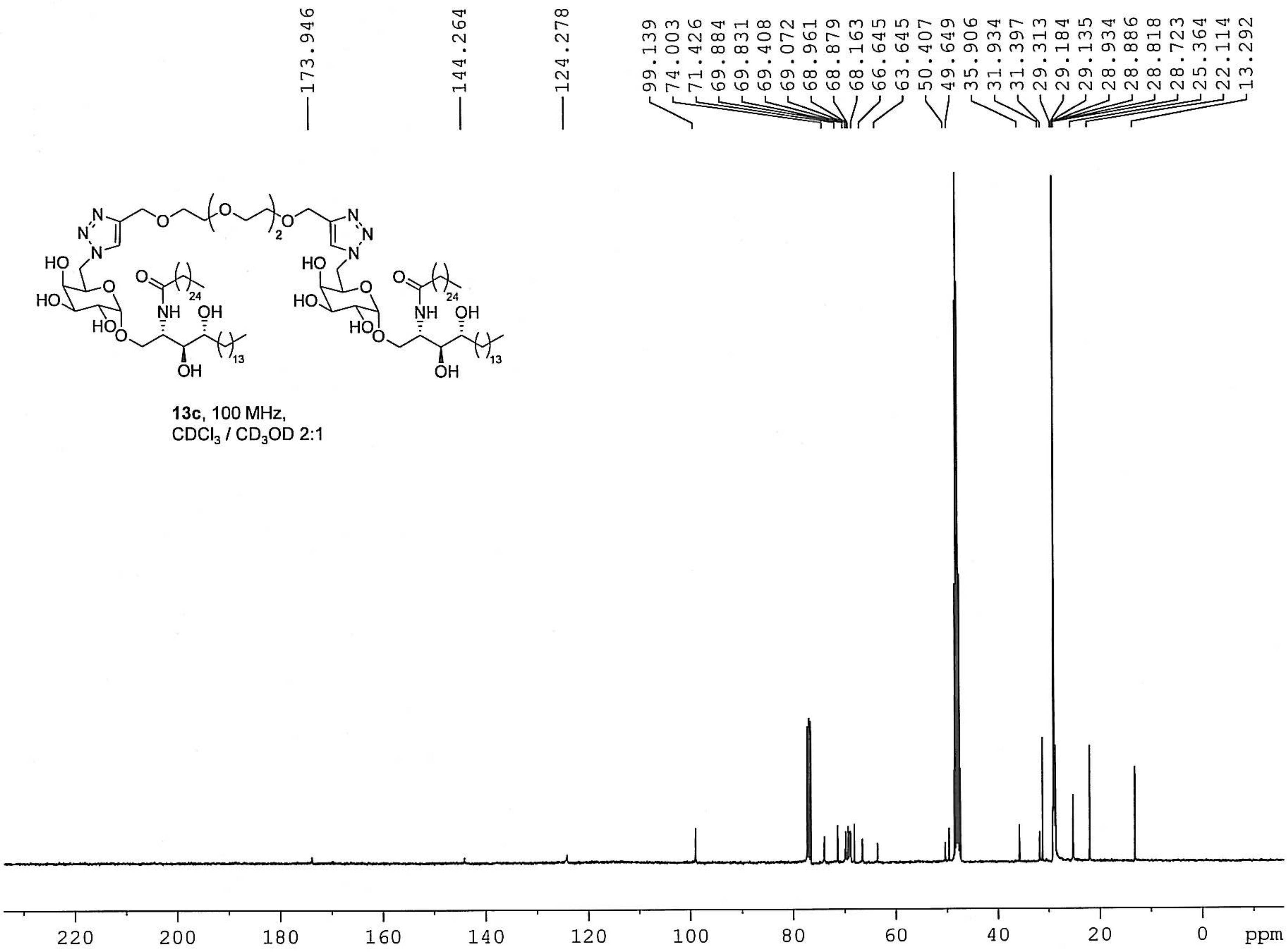

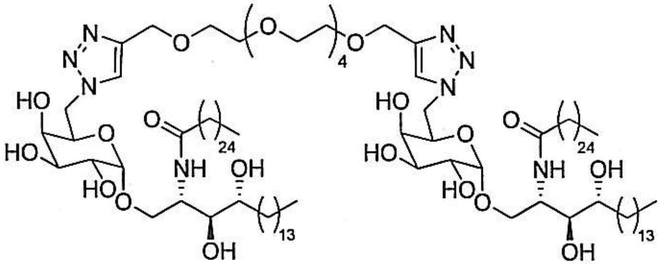

13d, 300 MHz,
$CDCl_3$ / CD_3OD 2:1

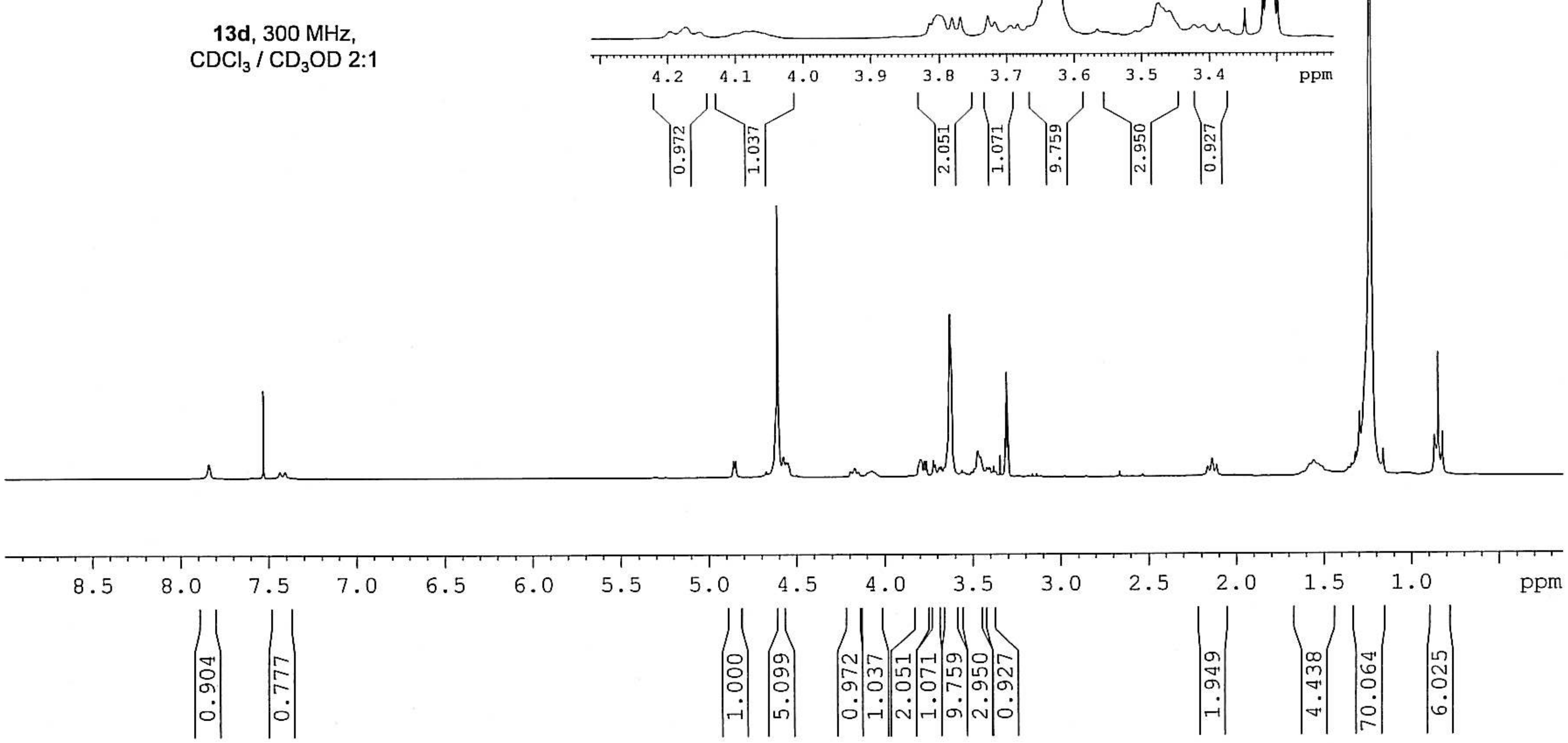

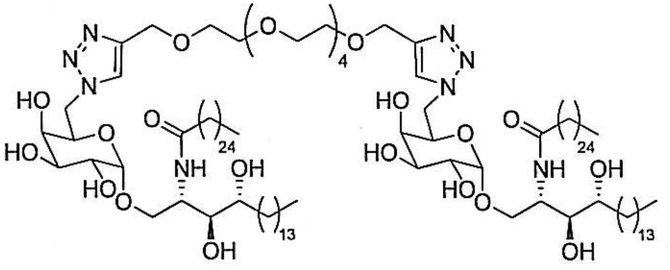

13d, 100 MHz,
$CDCl_3$ / CD_3OD 2:1

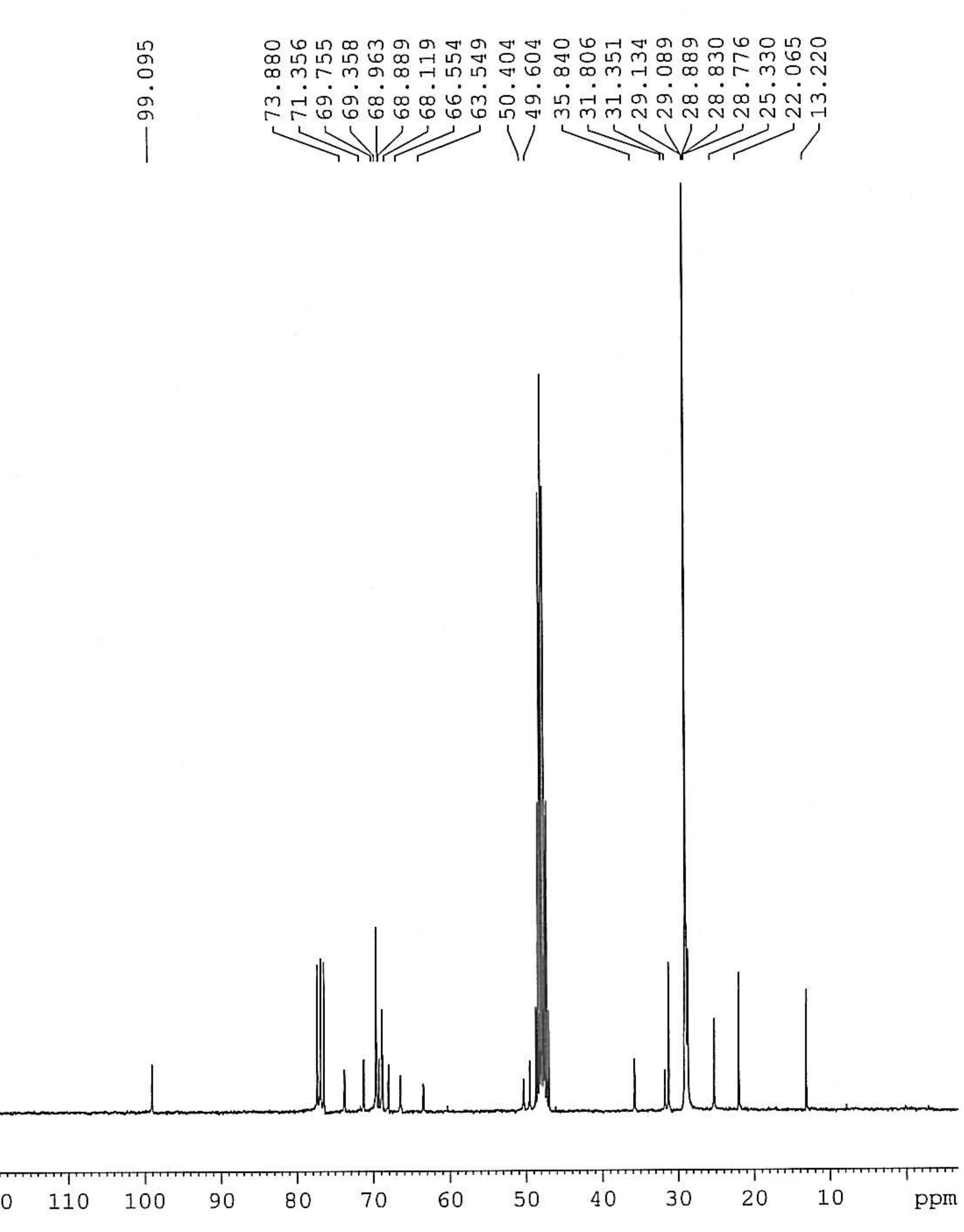

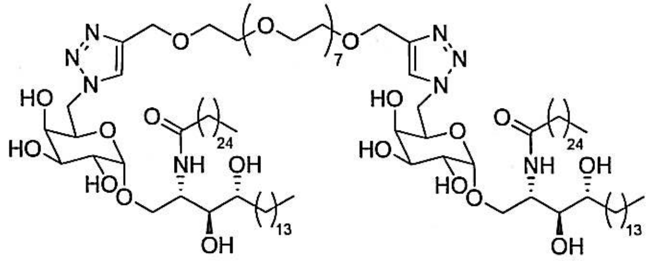

13e, 300 MHz,
$CDCl_3$ / CD_3OD 2:1

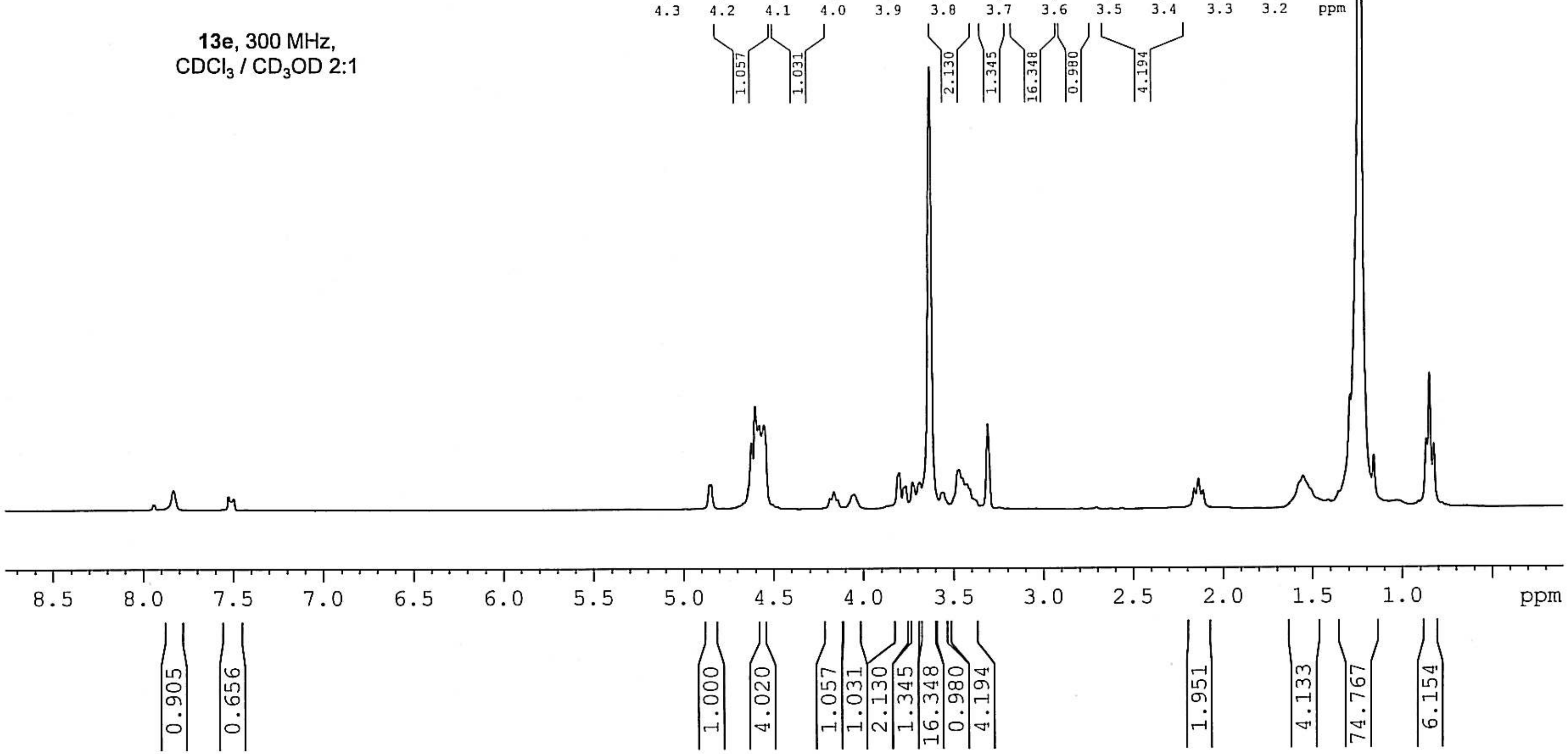

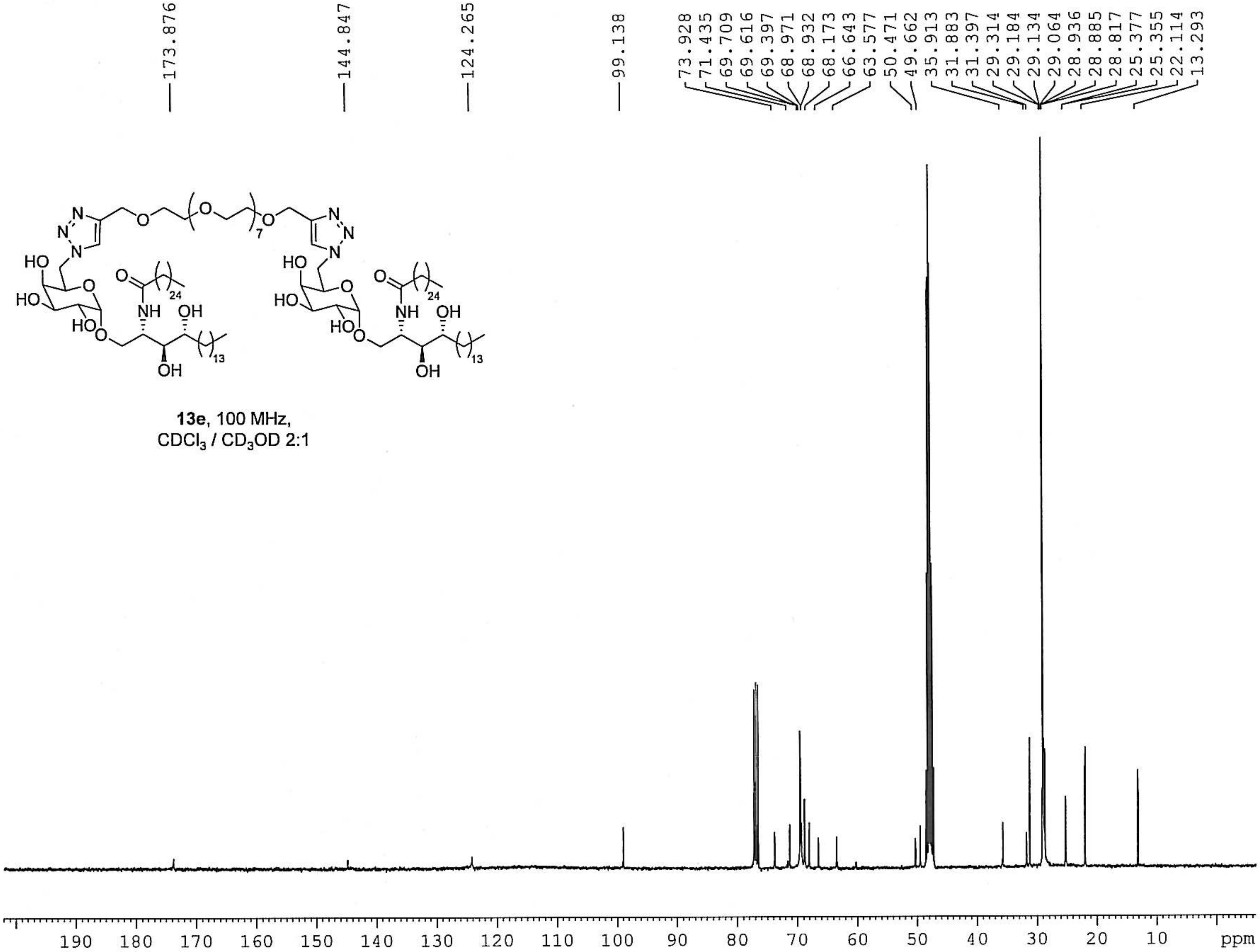

13e, 100 MHz,
$CDCl_3$ / CD_3OD 2:1

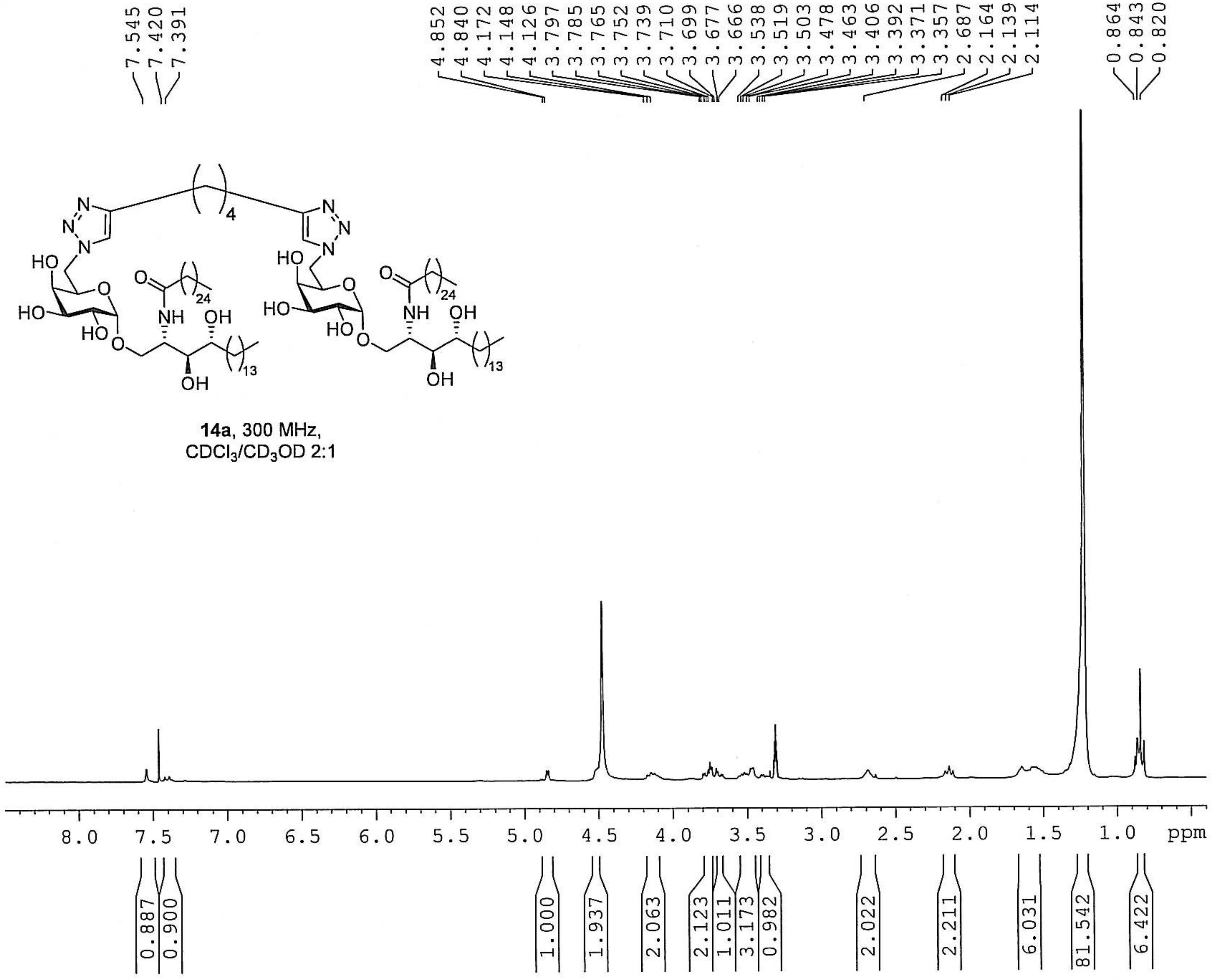

14a, 300 MHz,
$CDCl_3/CD_3OD$ 2:1

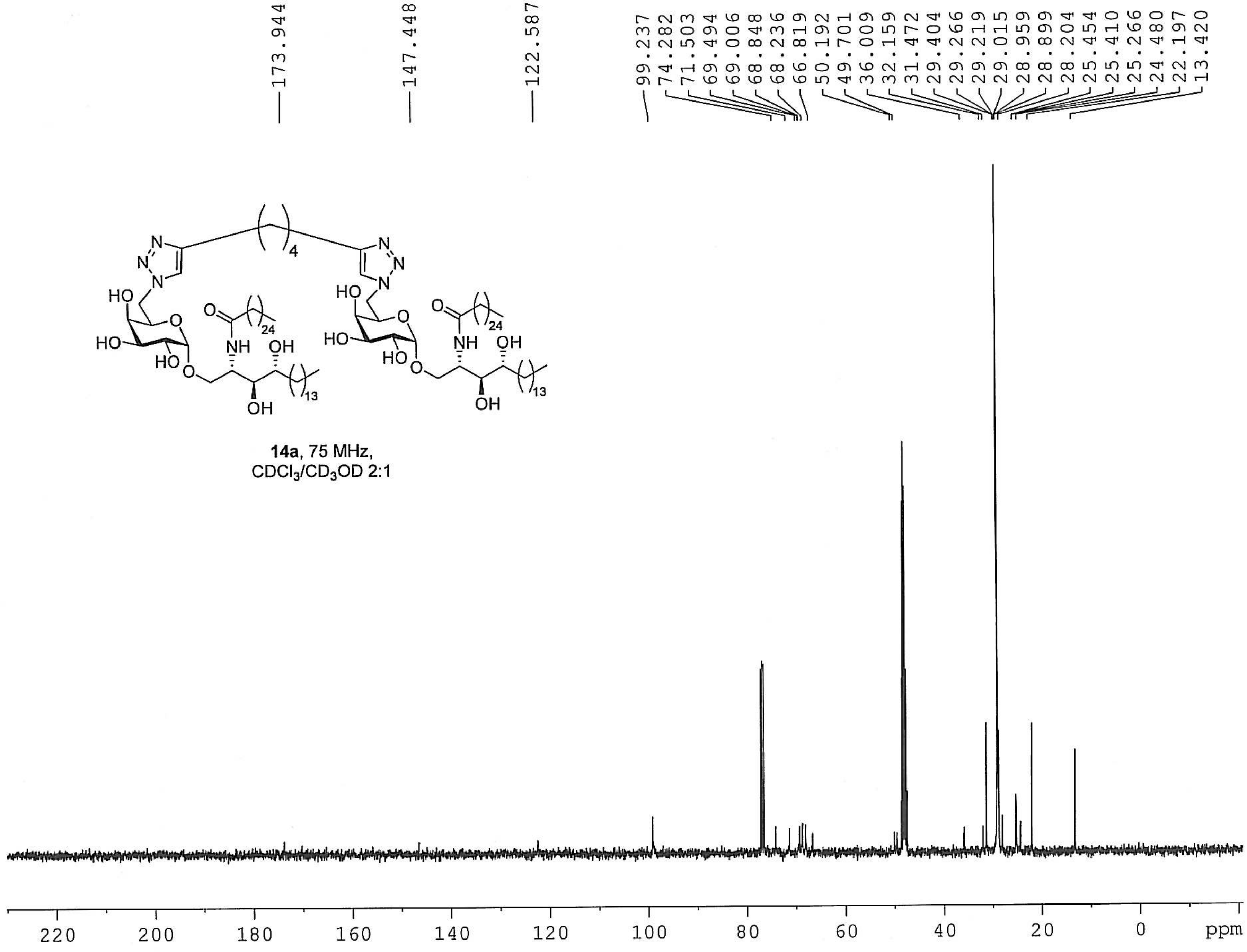

14a, 75 MHz, $CDCl_3/CD_3OD$ 2:1

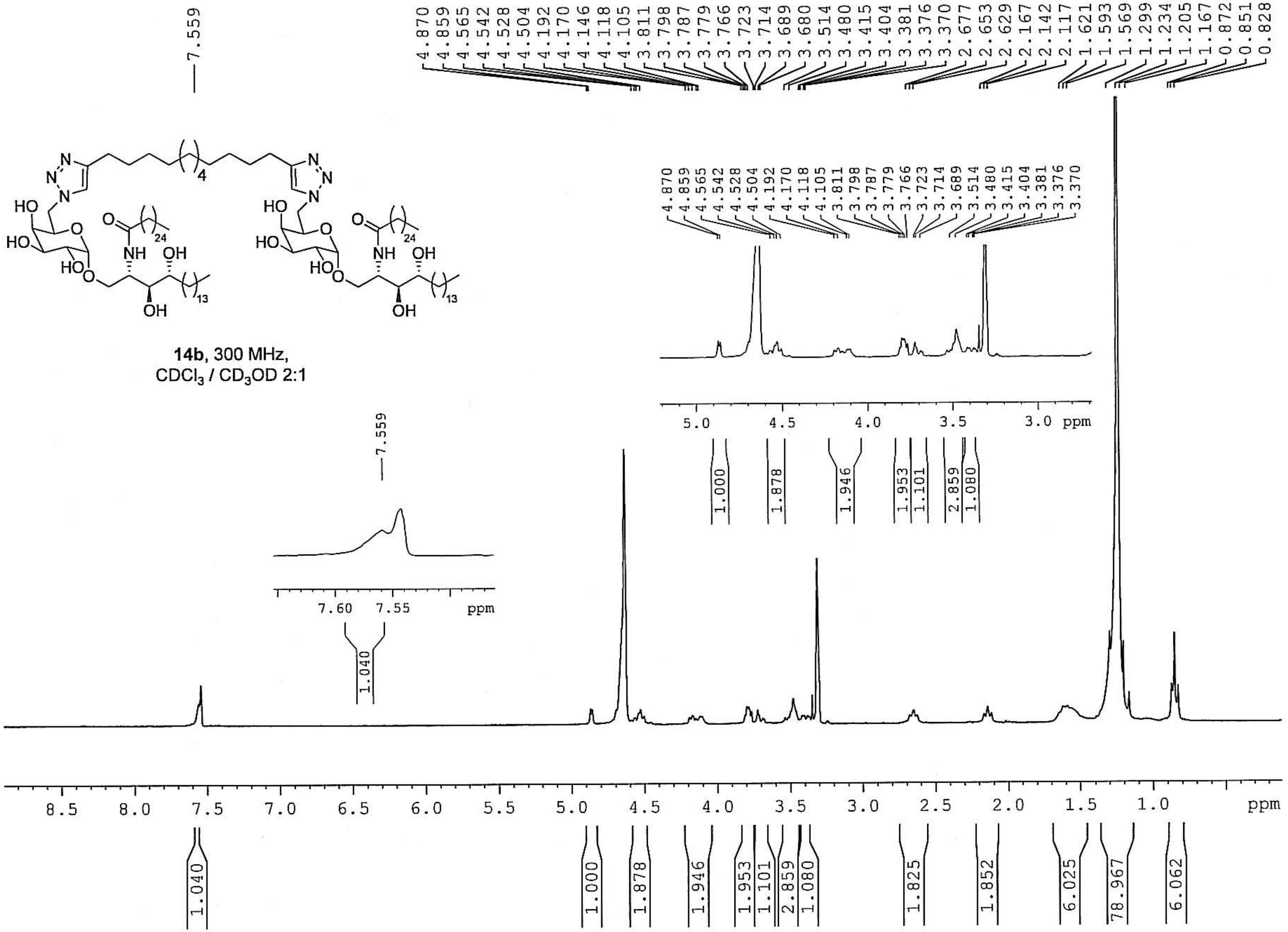

14b, 300 MHz,
$CDCl_3$ / CD_3OD 2:1

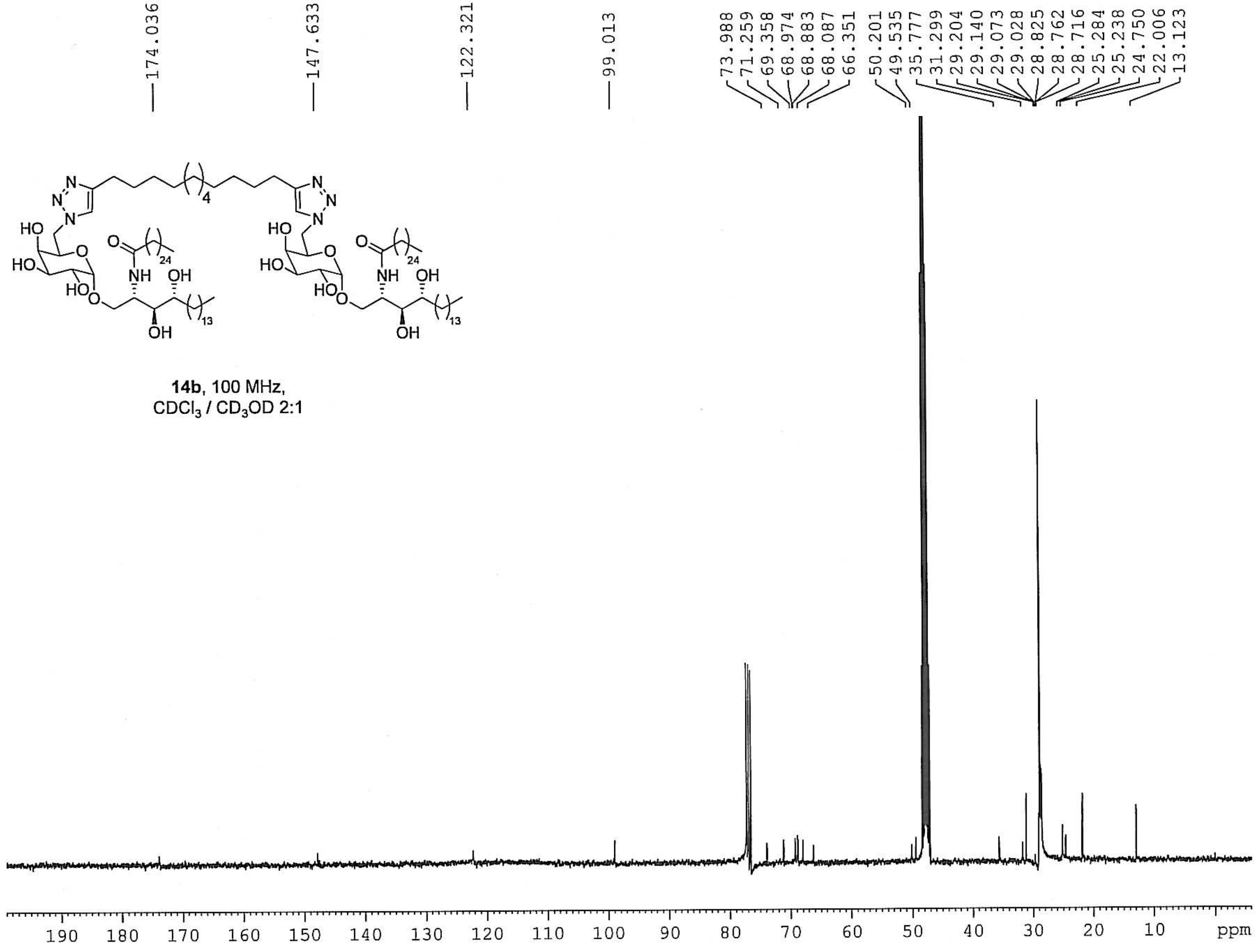

14b, 100 MHz,
$CDCl_3$ / CD_3OD 2:1

14c, 300 MHz,
$CDCl_3$ / CD_3OD 2:1

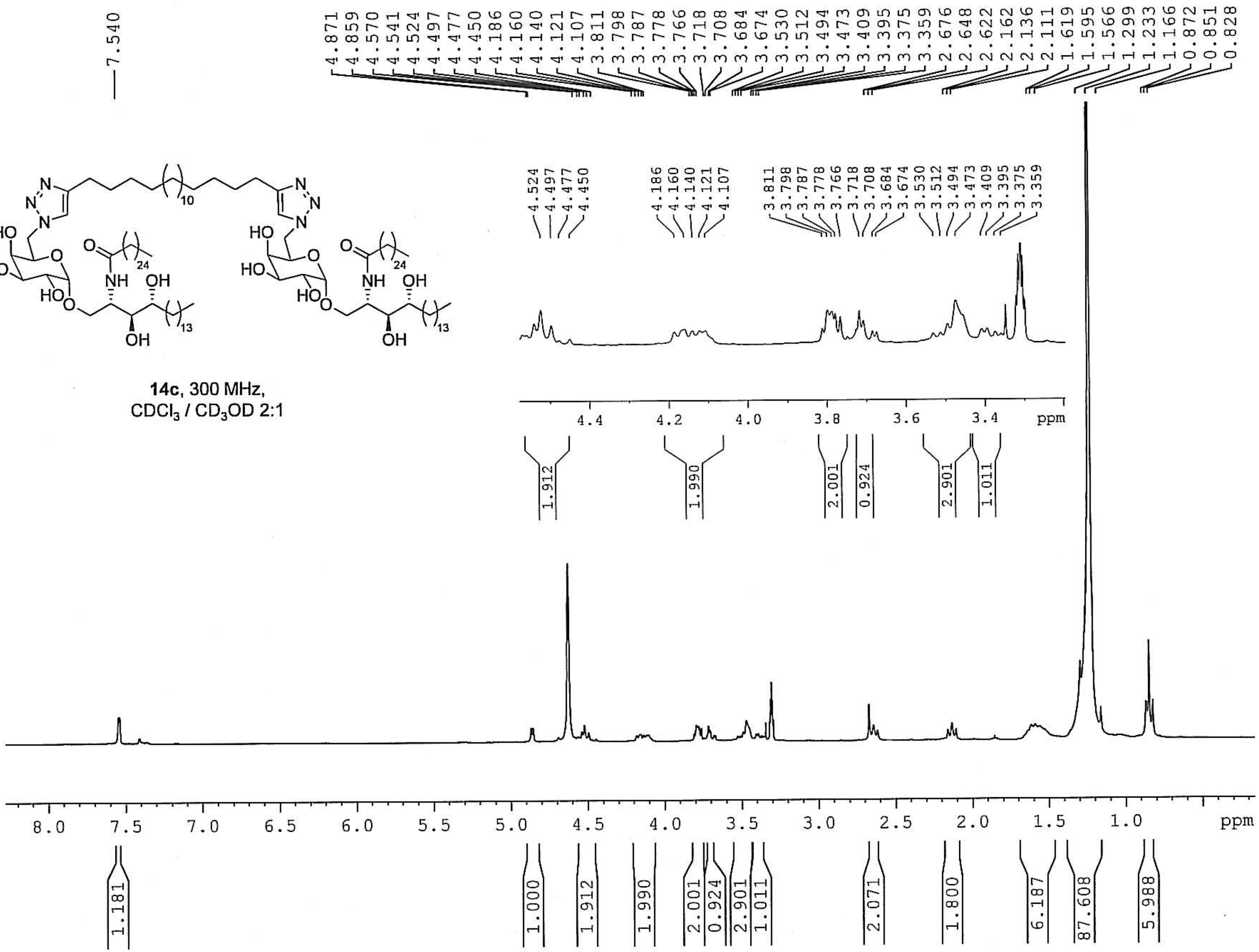

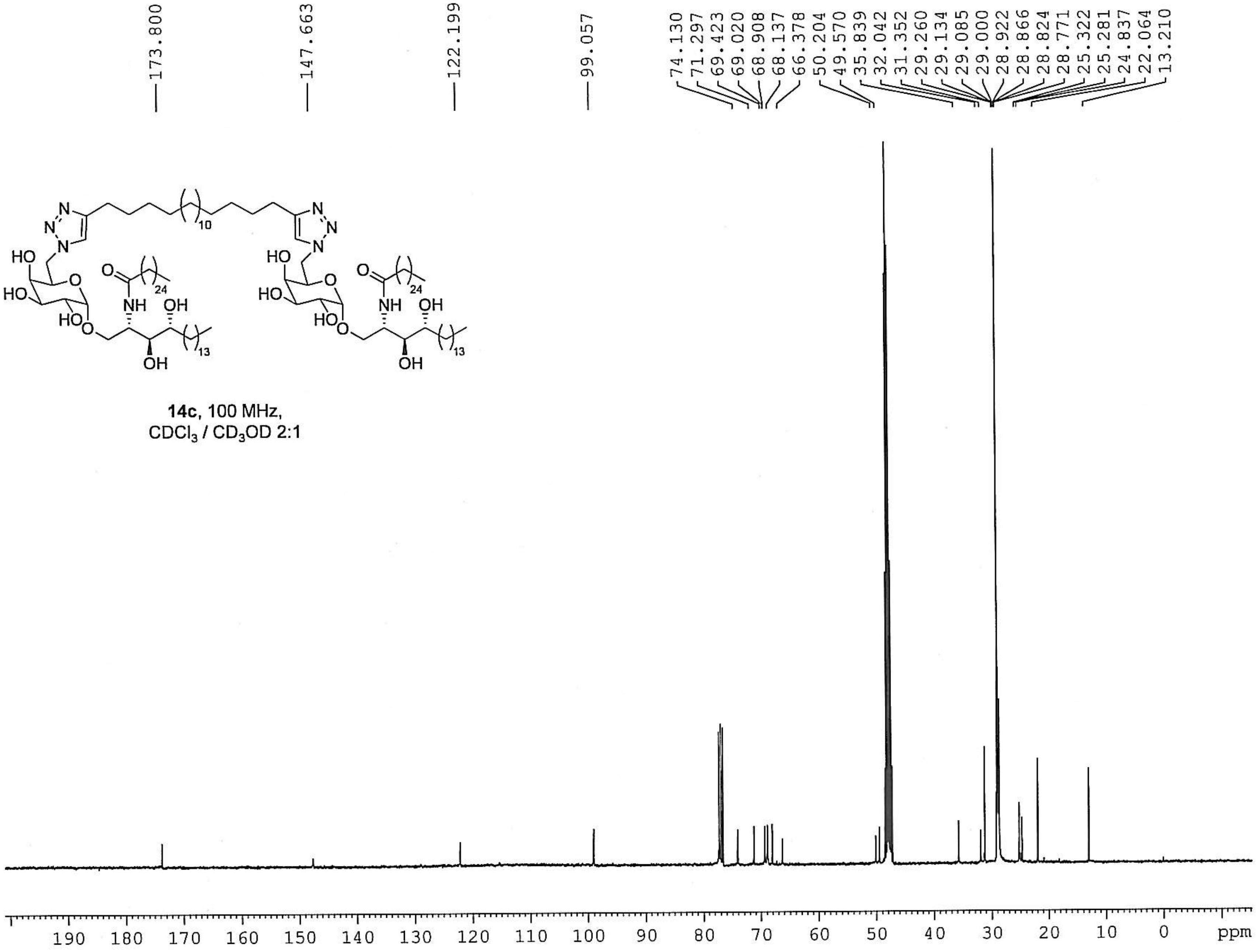

14c, 100 MHz,
$CDCl_3$ / CD_3OD 2:1
